# Comparative transcriptome profiling of upland (VS16) and lowland (AP13) ecotypes of switchgrass

**DOI:** 10.1007/s00299-016-2065-0

**Published:** 2016-11-03

**Authors:** Vasudevan Ayyappan, Malay C. Saha, Jyothi Thimmapuram, Venkateswara R. Sripathi, Ketaki P. Bhide, Elizabeth Fiedler, Rita K. Hayford, Venu (Kal) Kalavacharla

**Affiliations:** 1 Molecular Genetics and Epigenomics Laboratory, College of Agriculture and Related Sciences, Delaware State University, Dover, DE USA; 2 Forage Improvement Division, The Samuel Roberts Noble Foundation, Ardmore, OK USA; 3 Bioinformatics Core, Purdue University, West Lafayette, IN USA; 4 Plant Molecular Biology and Bioinformatics Laboratory, College of Agricultural, Life and Natural Sciences, Alabama A&M University, Normal, AL USA; 5 Center for Integrated Biological and Environmental Research, Delaware State University, Dover, DE USA

**Keywords:** Bioenergy, Lowland, Upland, Transcriptome, Switchgrass

## Abstract

**Key message:**

**Transcriptomes of two switchgrass genotypes representing the upland and lowland ecotypes will be key tools in switchgrass genome annotation and biotic and abiotic stress functional genomics.**

**Abstract:**

Switchgrass (*Panicum* *virgatum* L.) is an important bioenergy feedstock for cellulosic ethanol production. We report genome-wide transcriptome profiling of two contrasting tetraploid switchgrass genotypes, VS16 and AP13, representing the upland and lowland ecotypes, respectively. A total of 268 million Illumina short reads (50 nt) were generated, of which, 133 million were obtained in AP13 and the rest 135 million in VS16. More than 90% of these reads were mapped to the switchgrass reference genome (V1.1). We identified 6619 and 5369 differentially expressed genes in VS16 and AP13, respectively. Gene ontology and KEGG pathway analysis identified key genes that regulate important pathways including C4 photosynthesis, photorespiration and phenylpropanoid metabolism. A series of genes (33) involved in photosynthetic pathway were up-regulated in AP13 but only two genes showed higher expression in VS16. We identified three dicarboxylate transporter homologs that were highly expressed in AP13. Additionally, genes that mediate drought, heat, and salinity tolerance were also identified. Vesicular transport proteins, syntaxin and signal recognition particles were seen to be up-regulated in VS16. Analyses of selected genes involved in biosynthesis of secondary metabolites, plant–pathogen interaction, membrane transporters, heat, drought and salinity stress responses confirmed significant variation in the relative expression reflected in RNA-Seq data between VS16 and AP13 genotypes. The phenylpropanoid pathway genes identified here are potential targets for biofuel conversion.

**Electronic supplementary material:**

The online version of this article (doi:10.1007/s00299-016-2065-0) contains supplementary material, which is available to authorized users.

## Introduction

Switchgrass (*Panicum* *virgatum* L.) is an important, warm season, C4 perennial grass. Switchgrass was selected as a dedicated feedstock for the production of biofuels by the US Department of Energy (DOE). Although it is native to North America, it is grown in South America, Europe and Asia (Parrish et al. 2012). Based on plant morphology and adaptation area, switchgrass has been classified into two distinct ecotypes, lowland and upland (Moser and Vogel 1995; Porter 1966). The lowland ecotypes are mainly adapted to flood plains of the southern USA and characterized by tall and coarse stems, long-wide leaves, high biomass potential, and relatively tolerant to pests and disease (Sanderson et al. 1996). The upland ecotypes are mainly adapted in the dry and cool habitats in the northern USA. Plants of this ecotype have short and narrow stems and leaves, usually less productive, and are more susceptible to damage by pests and disease (Sanderson et al. 1996). Conversely, upland ecotypes are more drought and cold tolerant than lowland ecotypes. Improvement of both ecotypes is very important to meet the one billion dry ton biomass production target of the DOE by 2030 (US DOE 2011). Identification of ecotype-specific genes associated with inherent biotic and abiotic tolerance, and understanding their role and expression pattern can greatly aid in switchgrass crop improvement.

Earlier gene expression studies in switchgrass mostly relied on expressed sequence tag (EST)-based sequencing (Tobias et al. 2008) and microarrays (Sharma et al. 2012). RNA sequencing (RNA-Seq) has been widely used in providing genome-wide transcript profiles in model and non-model organisms including complex polyploid plant species such as switchgrass. So far limited reports are available on switchgrass transcriptome analysis. The first transcriptomic study using the Roche 454 sequencing platform generated ~40,000 transcripts from 980,000 reads and 243,600 contigs in switchgrass (Wang et al. 2012). Transcriptomic analysis of nodes and buds from high and low tillering switchgrass identified several transcription factors involved in the regulation of genes that determine axillary bud initiation and development (Wang et al. 2013a). Comparative transcriptome analysis of an upland cultivar (“Summer”) and a lowland cultivar (“Kanlow”) revealed early on-set of dormancy in crowns and rhizomes of Summer plants when compared to Kanlow (Palmer et al. 2014). Screening of switchgrass flag leaf transcriptomes helped in identification of molecular patterns in leaf development, senescence, and mineral utilization (Palmer et al. 2015).

RNA-Seq data has been used to understand genes associated with biomass production in switchgrass (Meyer et al. 2014) and *P. hallii* (Hall’s Panicgrass) (Meyer et al. 2012). Recently, role of plant transcription factors (TFs) in the development of lignocellulosic feedstocks for biofuels has also been suggested (Wuddineh et al. 2015). In switchgrass, transcriptome profiling for rust resistance (biotic stress) identified 84,209 transcripts from 98,007 gene loci from eight samples (Serba et al. 2015). Two abiotic stress-(drought- and heat-) responsive transcriptomes have been developed in switchgrass (Meyer et al. 2014; Li et al. 2013). Using comparative transcriptome analyses of four monocot species, 16 common genes have been identified as heat-responsive that were implicated in protein refolding (Li et al. 2013). The role of microRNAs in drought and salinity stress in switchgrass has also been reported (Xie et al. 2014). AP13 is the lowland switchgrass genotype used of genome sequencing. VS16 is the upland genotype deeply sequenced at the JGI to aid in genome assembly. These two genotypes are the parents of a mapping population for which genetic linkage maps are available (Serba et al. 2013), used for comparative mapping, and QTL analyses. These two genotypes are very important to the switchgrass research community. Moreover, a comprehensive comparative transcriptome profiling of both upland and lowland ecotypes targeting genes associated with biomass production, biotic and abiotic tolerance in unstressed plants is not available. Identification of ecotype-specific genes associated with inherent biotic and abiotic stress tolerance, and understanding their role and expression pattern can greatly aid in switchgrass crop improvement.

This study specifically aimed at: (1) developing reference transcriptomes of VS16 and AP13 genotypes in switchgrass; and (2) conducting comparative and targeted transcriptome analysis of VS16 and AP13 genotypes to identify differentially expressed biomass-related (including phenylpropanoid synthesis genes), biotic and abiotic tolerant genes that are inherent to upland and lowland ecotypes of unstressed switchgrass plants.

## Materials and methods

### Plant materials and sample collection

Two contrasting genotypes from two ecotypes of switchgrass, AP13 and VS16 were utilized in this study. AP13 is derived from the lowland cultivar, Alamo, and VS16 is derived from the upland cultivar, Summer. Both genotypes are tetraploids with a chromosome number of 2*n* = 4*x* = 36. Both ecotypes are actively grown during summer, hence the conditions considered in the study were not in favor of any specific ecotype. The genotypes were grown at 29/22 °C day/night temperatures and a 16-h photoperiod in the greenhouse at The Samuel Roberts Noble Foundation (NF) Ardmore, OK. Leaves were collected from one-month-old plants, frozen in liquid nitrogen, and stored at −80 °C until they were used.

### RNA Isolation and cDNA Synthesis

Total RNA was extracted from frozen VS16 and AP13 leaf samples using RNeasy plant mini kit (#74904, Qiagen Inc., Valencia, CA) according to the manufacturer’s instruction. Total RNA was treated with DNase I (#AM1904, Ambion, Grand Island, NY) to remove genomic DNA. The Nanodrop 2000 spectrophotometer (Thermo Scientific, Wilmington, DE) was used to determine reagent and protein contamination (indicated by the A260/A280 nm and A260/A230 nm ratios), in the RNA. The A260/A280 nm ratios for all samples ranged between 1.8 and 2.1 (majority of samples had 2.1). The quality of the RNA was determined on 1% agarose gel electrophoresis and Bioanalyzer 2100 (Agilent Technologies, Santa Clara, CA) for 28S/18S rRNA band intensity (2:1) and RNA integrity number (RIN) >8. Samples used for cDNA synthesis, Illumina sequencing, and downstream validation were of high quality (RIN > 8.0). MRNA from VS16 and AP13 was reverse transcribed to first-stand complementary DNA (cDNA) with Oligo dT using Protoscript II First Strand cDNA Synthesis kit (#E6560S, New England Biolabs, Ipswich, MA) according to the manufacturer’s instruction. RNA (1 µg) was denatured with Oligo dT at 65 °C for 5 min; subsequently, Protoscript II reaction mix and Protoscript II enzyme mix were added and incubated at 42 °C for 1 h. The enzyme was denatured at 80 °C for 5 min, and cDNA was quantified using Nanodrop 2000 spectrophotometer.

### Library construction and sequencing

RNA quality and purity was checked with a Fragment Analyzer (Advanced Analytical, Ames, IA), then RNA-Seq libraries were prepared with Illumina TruSeq Stranded mRNA Sample Preparation Kit (#RS-122-2101, Illumina Inc., San Diego, CA) as per the manufacturer’s instruction at the Delaware Biotechnology Institute, Newark, DE, USA. The experiment included three replicates and two genotypes, which resulted in six RNA-Seq libraries. The libraries generated in this study were labelled as AP13Rep1, AP13Rep2, AP13Rep3, VS16Rep1, VS16Rep2, and VS16Rep3 (Table 1). These libraries were sequenced on Illumina HiSeq 2500 platform with 50 nucleotide single-end reads. Sequences were submitted to the SRA section of NCBI with a bio-project number PRJNA297037.

**Table 1:** Summary statistics of RNA-Seq reads (Illumina/HiSeq 2500) collected from three technical replicates of lowland AP13 and upland VS16 genotypes of switchgrass

Sample ID	Total reads	Total trimmed reads	Reads passing QC (%)	Reads mapped	Reads mapped (%)	Reads with gene counts	Reads with gene counts (%)
AP13Rep1	43196737	43106501	99.79	39131924	90.8	18879959	48.24
AP13Rep2	50085105	49972031	99.77	45549852	91.2	17859109	39.20
AP13Rep3	40114835	40031015	99.79	36632734	91.5	16913617	46.17
VS16Rep1	46487777	46393034	99.80	42595217	91.8	25849058	60.68
VS16Rep2	46895932	46808693	99.81	42878187	91.6	24777723	57.78
VS16Rep3	42174644	42074699	99.76	37274103	88.6	18723830	50.23

### Transcriptome analysis

FASTQC was used to evaluate sequence quality. Raw reads obtained from RNA-Seq were trimmed for adapters and low-quality reads were filtered out (Phred score < 30) using FASTX Toolkit (v 0.0.13). High quality reads with 30 bases or more were retained and mapped against the reference switchgrass genomic sequences available at Phytozome v 1.0 (Goodstein et al. 2012) using TopHat v 2.0.11 (Trapnell et al. 2009) with default parameters. Cufflinks programs suite (Trapnell et al. 2012) was then used to analyze the data and to identify differential gene expression.

### Gene ontology (GO) and pathway analysis

Functional annotation of differential expression and gene enrichment was carried out by AgriGO analysis tool (Zhou et al. 2010). The hypergeometric test with multiple adjustments was used for GO analysis and was categorized into different classes (Falcon and Gentleman 2008). Further, pathways were assigned to GO classes using Kyoto Encyclopedia of Genes and Genomes (KEGG; http://www.genome.jp/kegg/kegg2.html).

### Reverse transcriptase-PCR (RT-PCR) and quantitative real-time (qRT-PCR) analysis

The synthesized cDNA was used for both conventional PCR and quantitative real-time PCR (qRT-PCR). The primers for RT-PCR were designed based on highly expressed transcripts (log2FC > 2) from RNA-Seq data sets. For RT-PCR, three genes each were selected from pathways representing heat-, drought-, and salinity-responsive genes. The specifics of genes and their respective primers for RT-PCR are given (Table S1). We used *cons7* as an internal control for normalization. Primers were designed by using Primer 3 (v0.4.0) online tool and synthesized by GenScript USA Inc. (Piscataway, NJ). For qRT-PCR, we selected five genes (other than those used for RT-PCR), that were differentially expressed based on our RNA-Seq analysis (Meyer et al. 2014), to verify that the RNA-Seq data reflects true quantification of gene expression. Switchgrass ecotypes have distinct geographic niches, thus their morphology is largely influenced by environmental conditions. Lowland ecotypes are relatively more tolerant to pests and disease (biotic stress) than upland ecotypes. Upland ecotypes are adapted in north (cold tolerant, grown well in semi-arid climate) but the lowland ecotypes are adapted in the south (cold susceptible, well adapted in flood plains). Sodium levels were significantly higher in lowland than in upland ecotypes (Yang et al. 2009). Validation of some differentially expressed biotic and abiotic stress-responsive genes in these two genotypes (AP13 and VS16) obtained from two distinct ecotypes (lowland and upland) would be interesting, which influenced us to initiate this validation experiment. Primer sequences of the five selected genes and a reference (*cons7*) are given (Table S2). Primers were designed using the online tool for real-time PCR (TaqMan) primer design (GenScript USA Inc., Piscataway, NJ) and utilized for quantitative determination of gene expression. To test validity of primers for qRT-PCR, they were first tested with conventional PCR using cDNA as the template. To do this, 10 ng of cDNA was utilized for the reactions under standard PCR conditions (98 °C for 30 s, 55 °C for 30 s and 72 °C for 1 min) for 35 cycles. The amplified products were separated in a 1% agarose gel and visualized by using ethidium bromide staining. After primer validation, qRT-PCR was performed in 25 µl reactions that contained 10 ng of cDNA, 10 µM of primer pairs (FW and REV) and 12.5 µl of SYBR Green PCR Master Mix (#4309155, ThermoFisher Scientific Inc., Grand Island, NY). PCR conditions used for qRT-PCR were as follows: 95 °C for 10 min, followed by 40 cycles of 95 °C for 15 s and 65 °C for 1 min. In this study, we used three replicates for the qRT-PCR analysis. The internal control gene *cons7* was used to normalize the results in all tissue samples. The efficiency of primers was tested and the 2-ΔΔCT method (Livak and Schmittgen 2001) was used to analyze the results where ΔΔCT = (CT of gene—CT of *cons7*) sample to be observed—(CT of genex—CT of *cons7*). Minitab-17 software (State College, PA) was used to analyze the normalized CT values (ΔΔCT) that were collected from qRT-PCR analysis and the expression results were presented as mean ± SE. For multiple comparisons, one-way ANOVA was performed on qRT-PCR experiments between the mean of samples.

## Results

To understand differential expression of genes between the two distinct switchgrass genotypes, AP13 and VS16, we conducted RNA-Seq analyses and compared their expression profiles. We comprehensively cataloged and described not only the highly expressed transcripts identified in these genotypes, but also identified significant differences between them and relate such differences to the genetic and physiological variability of the switchgrass ecotypes. We have first outlined the highly expressed genes (log2FC > 2) in the main manuscript, while remaining transcripts are reported in the supplementary files.

### Sequence data

Deep sequencing of six RNA-Seq libraries yielded ~268 million-50 nt Illumina reads that were uniformly distributed between two genotypes with ~133 million reads from AP13, and ~135 million reads from VS16 (Table 1). More than 90% of the RNA-Seq reads mapped to the reference genome and only uniquely mapped reads with ≤2 mis-matches were further used in the analysis. Reads with gene counts was much higher in VS16 (56.23%) than in AP13 (44.54%) (Table 1).

### Transcriptome analyses

**Fig. 1:**
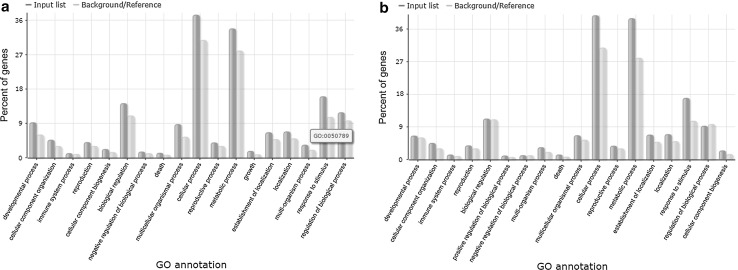
AgriGo analysis of significantly enriched genes from RNA-Seq analysis of two switchgrass ecotypes: **a** AP13 and **b** VS16

Genome-wide transcriptome analyses identified approximately 11,988 transcripts that were differentially expressed between VS16 and AP13. Among these, 6619 transcripts were found to be expressed at higher levels in VS16, while 5369 genes were significantly expressed in AP13. Functional analysis of differentially expressed genes using AgriGO indicated that the majority of genes were found to be associated with cellular and metabolic processes followed by responses to stimulus and stress (Fig. 1). The AgriGO annotation pattern is similar in both genotypes. The differentially expressed genes were further annotated, and their representative pathways assigned and categorized into different classes based on their functional roles.

### Cataloging of diverse pathways

KEGG pathway analysis of differentially expressed transcripts identified more than 300 pathways that were common to both the genotypes. Top eight pathways identified in both genotypes along with important genes in each category are presented below (number of genes in parentheses). In VS16, these included metabolic processes (352), secondary metabolite biosynthesis (178), biosynthesis of antibiotics (100), ribosome (78), microbial metabolism in diverse environments (62), biosynthesis of amino acids (59), purine metabolism (43), and carbon metabolism (40) (Table 2; Supplementary Table S3). In AP13 these pathways included metabolic processes (344), secondary metabolite biosynthesis (183), biosynthesis of antibiotics (75), microbial metabolism in diverse environments (62), biosynthesis of amino acids (47), carbon metabolism (40), photosynthesis (33), and oxidative phosphorylation (32) (Table 2; Supplementary Table S3). Though several significant pathways were identified in this study, we have focused more on bioenergy affiliated genes such as those associated with photosynthesis pathways, C4 photosynthesis, photorespiration, and phenylpropanoid pathways that contribute to biomass production.

**Table 2:** Number of genes identified from respective KEGG pathways in lowland AP13 and upland VS16 genotypes of switchgrass

KEGG ID	Description	Number of Genes	
		VS16	AP13
ko01100	Metabolic pathways	352	344
ko01110	Biosynthesis of secondary metabolites	178	183
ko01130	Biosynthesis of antibiotics	100	75
ko03010	Ribosome	78	27
ko01120	Microbial metabolism in diverse environments	62	62
ko01230	Biosynthesis of amino acids	59	47
ko00230	Purine metabolism	43	23
ko01200	Carbon metabolism	40	40
ko04141	Protein processing in endoplasmic reticulum	38	12
ko00240	Pyrimidine metabolism	38	19
ko00195	Photosynthesis	2	33
ko03040	Spliceosome	24	34
ko00190	Oxidative phosphorylation	37	32

### Photosynthesis pathway

**Fig. 2:**
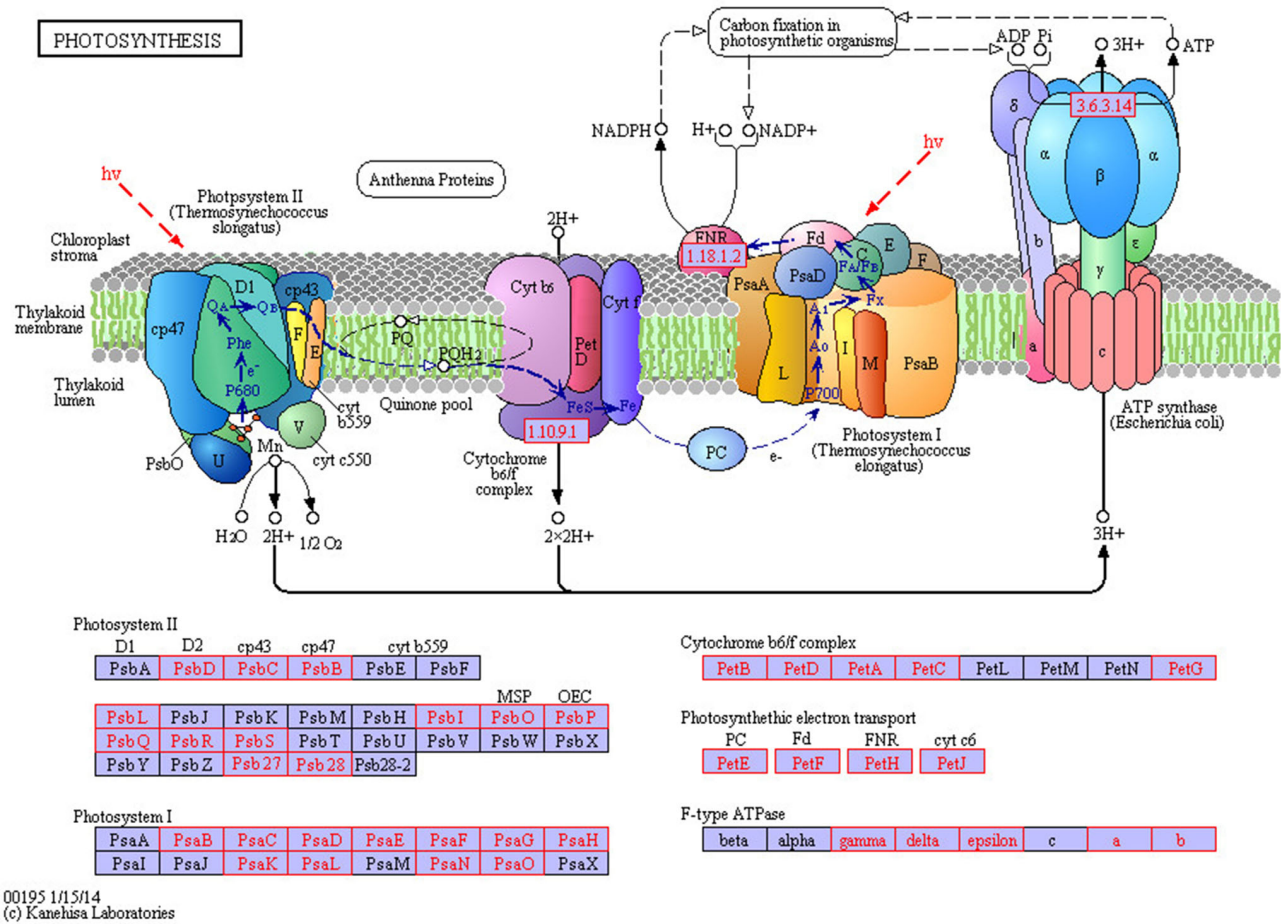
Putative photosynthesis-related KEGG pathway (ID: Ko00195) identified in switchgrass ecotype, AP13. The *boxes* highlighted in *red* are enzymes identified in this study (color figure online)

To identify differential expression of key genes between lowland and upland ecotypes, KEGG pathways were analyzed specifically for photosystem-related transcripts (Fig. 2). It was observed that 33 genes were up-regulated in AP13 whereas only two genes were up-regulated in VS16 (log2FC > 2). The photosystem II-related genes, PsbD, PsbC, PsbB, PsbI, PsbO, PsbP, PsbQ, PsbR, PsbS, Psb27, Psb28; photosystem I-related genes, PsaB, PsaC, PsaD, PsaE, PsaG, PsaH, PsaK, PsaL, PsaO; cytochrome b6/f complex-related genes, PetB, PetD, PetC, PetG; photosynthetic electron transport genes PetE, PetF, PetH, PetJ, and genes responsible for F-type ATPase, gamma-, delta-, epsilon-chl a and b were significantly expressed in AP13. In VS16, only PetF and PetH involved in photosynthetic electron transport were seen to be up-regulated.

### C4 photosynthesis-related enzymes

Among the enzymes associated with C4 photosynthesis, eight key enzymes; i.e., phosphoenolpyruvate (PEP, EC 3.4.11), phosphoenolpyruvate carboxylase (PEPC, EC 4.1.1.31), phosphoenolpyruvate carboxykinase (PEP-CK, EC 4.1.1.49), phosphoenolpyruvate/phosphate translocator (PPT), mitochondrial NAD-dependent malic enzyme (m-NAD-ME, EC 1.1.1.39), glucose 6-phosphate/phosphate translocator (GPT), carbonic anhydrase (CA, EC 4.2.1.1), and bile acid sodium symporter/pyruvate transporter (BASS) were significantly expressed in both VS16 and AP13 (Table 3). The photosynthetic pathway transcripts nicotinamide adenine dinucleotide phosphate-malic enzyme (NADP-ME, EC 1.1.1.40), NADP-dependent oxidoreductase (EC 1.6.1.1), phosphate translocator, and cytosolic triosephosphate isomerase were uniquely up-regulated in VS16 while pyruvate orthophosphate dikinase response protein (PDRP), sodium proton antiporter/H+/Na+ exchanger (NHD), chloroplastic triosephosphate isomerase (EC 5.3.1.1), dicarboxylate transporter (DIT), and aspartate aminotransferase (AspAT, EC 2.6.1.1) were overrepresented in AP13.

**Table 3:** Up- and down-regulated genes involved in C4-related photosynthesis, photorespiration pathway, and phenylpropanoid pathway in VS16 compared to AP13

Genes detected	>Twofold up-regulation	
	VS16	AP13
C4-related photosynthetic Genes		
Dicarboxylate transporter (DIT)	0	10
Phosphoenolpyruvate (PEP)	6	7
Glucose 6-phosphate/phosphate translocator (GPT)	4	7
Phosphoenolpyruvate carboxylase (PEPC)	6	6
Bile acid sodium symporter/pyruvate transporter (BASS)	2	4
Carbonic anhydrase (CA)	2	4
NADP-dependent oxidoreductase, putative, expressed	4	2
kinase (PEPC-K)	1	4
Phosphoenolpyruvate carboxykinase (PEP-CK)	1	4
Phosphoenolpyruvate/phosphate translocator (PPT)	1	3
Triosephosphate isomerase, cytosolic	3	0
Photorespiration-related Genes		
2-Oxoglutarate (2-OG)	22	23
Glutamate	17	4
3-Phosphoglycerate (3-PGA)	12	12
Ribulose-1,5-bisphosphate carboxylase/oxygenase (RuBisCO)	0	11
Glutamine (GLN)	8	6
Dicarboxylate transporter (DIT)	0	8
Catalase (CAT)	5	2
Glutamate synthase (GS)	1	4
Phenylpropanoid-related Genes		
Tyrosine	12	18
Phenylalanine ammonia-lyase (PAL)	8	4
Cinnamoyl CoA reductases (CCR)	8	3
4-Coumarate:CoA ligase (4CL)	5	1
Cinnamoyl-CoA reductase (CCR)	5	1
Hydroxycinnamoyl CoA:shikimate hydroxycinnamoyl transferase (HCT)	5	1
Cinnamyl Shikimate 3-Hydroxylase (CAD)	4	4
Caffeoyl-CoA 3-O-methyltransferase (CCoAMOT)	4	0

### Photorespiration pathway-related genes

Six key photorespiratory enzymes; 2-oxoglutarate (2-OG, EC 1.2.4.2), 3-phosphoglycerate (3-PGA, EC 1.1.1.95), glutamine (GLN, EC 1.4.1.13), phosphoglycolate phosphatase (PGLP, EC 3.1.3.18), serine glyoxylate aminotransferase (AGT, EC 2.6.1.45), and serine hydroxymethyltransferase (SHM, EC 2.1.2.1); were expressed in both the genotypes (Table 3). However, glutamate synthase (GS, EC 1.4.1.13), ribulose-1, 5-bisphosphate carboxylase/oxygenase (RuBisCO, EC 4.1.1.39), hydroxypyruvate reductase (HPR, EC 1.1.1.81), and dicarboxylate transporter (DIT) were highly expressed in AP13 while glutamate, catalase (CAT, EC 1.11.1.6), and glutamate-ammonia ligase (GAL, EC 2.7.7.42) were significantly expressed in VS16.

### Phenylpropanoid pathway-related genes

Genes involved in phenylpropanoid pathways play a key role in feedstock quality. Genes related to the phenylpropaniod pathways were identified and those abundantly expressed included phenylalanine ammonia-lyase (PAL, EC 4.3.1.24), cinnamate 4-hydroxylase (C4H, EC 1.14.13.11), 4-coumarate: CoA ligase (4CL, EC 6.2.1.12), hydroxycinnamoyl CoA: shikimate hydroxycinnamoyl transferase (HCT, EC 2.3.1.133), caffeoyl-CoA 3-O-methyltransferase (CCoAMOT, EC 2.1.1.104), cinnamoyl-CoA reductase (CCR, EC 1.2.1.44), cinnamyl alcohol dehydrogenase (CAD, EC 1.1.1.195), and ferulic acid. Among these genes, CAD was most dominantly expressed in both genotypes. Specifically, ferulic acid, CCR, PAL, 4CL, CCoAMOT, HCT, and C4H genes were uniquely expressed in VS16 (Table 3).

### Transcription factors

There were 625 differentially expressed transcripts (Log2FC > 2) classified as transcription factors (TFs) (Table 4; Supplementary Table S4). Of these, 428 and 197 transcripts displayed higher levels of expression in VS16 and AP13, respectively. Among these; No apical meristem, Arabidopsis transcription activation factor, and Cup-shaped cotyledon (NAC; 44); W-box containing transcription factor (WRKY; 28); myeloblastosis (MYB; 97); GIBBERELLIC-ACID INSENSITIVE (GAI), REPRESSOR of GAI (RGA) and SCARECROW (SCR) (GRAS; 26); basic leucine zipper domain (bZip; 33); basic helix-loop-helix (bHLH; 12); C2H2 (38); GCN5-related N-acetyltransferase (GNAT; 86) family; GATA (12); and dehydration-responsive element binding (DREB; 6) TFs were significantly enriched (Log2FC > 2) in both the genotypes. This is by comparing VS16 transcript profile with that of AP13. Normalized expression values were taken into consideration for analysis. In VS16, the most abundantly expressed TFs which were identified included: Myb domain protein 46 that control secondary cell wall biosynthesis; BTB-POZ and MATH domain 1 that mediate transcriptional repression and interacts with the components of histone deacetylase co-repressor; nuclear factor Y that regulates transcription; xylem NAC domain that is involved in xylem development; bZIP transcription factor that has a role in pathogen defense, light and stress signaling; GATA transcription factor 4 that binds selectively and non-covalently to DNA sequence GATA and growth regulating factor (GRF) zinc finger family proteins with the help of zinc ions. In AP13, the frequently expressed TFs identified included: Lin11, Isl-1 and Mec-3 (LIM) domain-containing proteins play a role in protein–protein interactions that are critical in cellular processes, zinc finger C2H2-type or integrase DNA-binding superfamily of proteins that modulate gene expression by binding to DNA and RNA, and Mothers against decapentaplegic (MAD) homolog/Forkhead-associated (SMAD/FHA) domain-containing protein that regulates transcription by participating in sequence-specific DNA binding.

**Table 4:** Representative up- and down-regulated transcription factors in VS16 compared to AP13

Gene ID	log2FC	Description
Pavir.Ca02370	8.11458	Myb domain protein 46; myb-like DNA-binding domain-containing protein, putative, expressed
Pavir.J19884	8.03805	BTB-POZ and MATH domain 1; MBTB32—Bric-a-Brac, Tramtrack, Broad Complex BTB domain with Meprin and TRAF Homology MATH domain, expressed
Pavir.Aa00318	7.61192	Nuclear factor Y, subunit A5; nuclear transcription factor Y subunit, putative, expressed
Pavir.Ga01785	7.56188	Xylem NAC domain 1; no apical meristem protein, putative, expressed
Pavir.J26815	7.50209	G-box binding factor 6; bZIP transcription factor domain-containing protein, expressed
Pavir.Fa00854	7.48631	General regulatory factor 7; 14-3-3 protein, putative, expressed
Pavir.Fa01426	6.98614	Basic leucine zipper 7; bZIP transcription factor domain-containing protein, expressed
Pavir.Ia02808	6.84614	GATA transcription factor 4; GATA zinc finger domain-containing protein, expressed
Pavir.J37973	6.79487	GRF zinc finger family protein
Pavir.J18139	6.69668	Myb domain protein 86; MYB family transcription factor, putative, expressed
Pavir.J36164	−4.62238	Zinc finger protein 2; ZOS9-12—C2H2 zinc finger protein, expressed
Pavir.Ia01299	−4.64532	LOB domain-containing protein 37; DUF260 domain-containing protein, putative, expressed
Pavir.Db01250	−4.65699	GATA type zinc finger transcription factor family protein; GATA zinc finger domain-containing protein, expressed
Pavir.Bb02239	−4.92765	Zinc finger protein 2; ZOS9-12—C2H2 zinc finger protein, expressed
Pavir.Ea02402	−5.01494	SMAD/FHA domain-containing protein; FHA domain-containing protein, putative, expressed
Pavir.Ba02999	−5.47064	Integrase-type DNA-binding superfamily protein; AP2 domain-containing protein, expressed
Pavir.Hb01240	−5.71164	Putative endonuclease or glycosyl hydrolase with C2H2-type zinc finger domain; ZOS9-06—C2H2 zinc finger protein, expressed
Pavir.Db01231	−5.93954	Basic leucine zipper (bZIP) transcription factor family protein; bZIP transcription factor domain-containing protein, expressed
Pavir.Ib01420	−6.95906	Myb domain protein 12; MYB family transcription factor, putative, expressed
Pavir.J24912	−7.05979	LIM domain-containing protein; LIM domain-containing protein, putative, expressed

### Understanding stress tolerance pathways

This study identified native gene expression in both AP13 and VS16 as a prelude to studying stress tolerance. Even though we did not impart any stress to derive the reference transcriptomes in this study, it was interesting that many biotic and abiotic stress tolerance genes were differentially expressed between AP13 and VS16. Therefore, changes noticed in gene expression represent inherent differences between the upland and lowland switchgrass ecotypes and should be taken into consideration while developing cultivars for specific switchgrass growing regions. It is inferred that ecotype-specific genes identified here influence biomass and biofuel production directly or indirectly.

### Abiotic stress tolerance genes

#### Drought tolerance genes

At least 24 drought tolerance related transcripts were identified between VS16 and AP13 (Table 5; Supplementary Table S5). VS16 has more drought tolerant genes compared to AP13. Of the 24 drought tolerant genes identified, transcripts representing 19 and five differentially expressed genes have been implicated in membrane and vacuolar transport, respectively (Table 5; Supplementary Table S5). In VS16, we identified six transcripts of NOD26-like intrinsic proteins (NIP), five tonoplast intrinsic proteins (TIP), two delta tonoplast integral proteins, and six plasma membrane intrinsic (PIP) proteins. In AP13, two transcripts that encoded for NOD26-like intrinsic proteins and three plasma membrane intrinsic proteins were identified.

**Table 5:** Significant up- and down-regulated drought- and heat-responsive genes in VS16 compared to AP13

Gene ID	Log2FC	Description
Drought-responsive genes		
Pavir.Ea00003	7.97093	Tonoplast intrinsic protein 4;1; aquaporin protein, putative, expressed
Pavir.Cb01832	6.85293	Tonoplast intrinsic protein 4;1; aquaporin protein, putative, expressed
Pavir.Ca00461	6.57124	Tonoplast intrinsic protein 4;1; aquaporin protein, putative, expressed
Pavir.Aa02820	5.95073	NOD26-like intrinsic protein 4;2; aquaporin protein, putative, expressed
Pavir.Ab01231	5.57162	NOD26-like intrinsic protein 1;2; aquaporin protein, putative, expressed
Pavir.Bb01841	−5.23059	Plasma membrane intrinsic protein 2; aquaporin protein, putative, expressed
Pavir.Ba01199	−5.25504	Plasma membrane intrinsic protein 3; aquaporin protein, putative, expressed
Heat-responsive genes		
Pavir.Ib01427	5.5287	Heat shock protein DnaJ with tetratricopeptide repeat; DNAJ heat shock N-terminal domain-containing protein, putative, expressed
Pavir.J24721	5.48338	Heat shock cognate protein 70-1; DnaK family protein, putative, expressed
Pavir.J41002	4.43179	DNAJ heat shock family protein; dnaJ domain-containing protein, expressed
Pavir.J40688	4.16865	DNAJ heat shock N-terminal domain-containing protein; dehydrin family protein, expressed
Pavir.Bb00967	4.03172	DNAJ heat shock N-terminal domain-containing protein; dnaJ domain-containing protein, expressed
Pavir.Ia03540	3.92154	Tetratricopeptide repeat (TPR)-like superfamily protein; DNAJ heat shock N-terminal domain-containing protein, putative, expressed
Pavir.Ab01923	3.62978	DNAJ heat shock N-terminal domain-containing protein; chaperone protein dnaJ 10, putative, expressed
Pavir.J23559	3.13939	Heat shock protein DnaJ with tetratricopeptide repeat; DNAJ heat shock N-terminal domain-containing protein, putative, expressed
Pavir.Ha01274	−3.60451	Chaperone DnaJ-domain superfamily protein; heat shock protein DnaJ, putative, expressed
Pavir.Ea02970	−3.64637	DNAJ heat shock N-terminal domain-containing protein; heat shock protein DnaJ, putative, expressed
Pavir.J12343	−3.65432	Heat shock protein 70; DnaK family protein, putative, expressed
Pavir.Cb01655	−3.8408	Chaperone DnaJ-domain superfamily protein; heat shock protein DnaJ, putative, expressed
Pavir.J35718	−3.84138	DNAJ heat shock N-terminal domain-containing protein; heat shock protein DnaJ, putative, expressed
Pavir.J16614	−3.85727	Chaperone DnaJ-domain superfamily protein; heat shock protein DnaJ, putative, expressed
Pavir.J13048	−3.87888	Chaperone DnaJ-domain superfamily protein; heat shock protein DnaJ, putative, expressed
Pavir.Cb00021	−3.97194	Chaperone DnaJ-domain superfamily protein; heat shock protein DnaJ, putative, expressed
Pavir.Cb00837	−4.09747	Chaperone DnaJ-domain superfamily protein; heat shock protein DnaJ, putative, expressed
Pavir.Ca02433	−4.1177	Chaperone DnaJ-domain superfamily protein; heat shock protein DnaJ, putative, expressed

#### Heat tolerance genes

This study identified 65 genes involved in heat tolerance by comparing VS16 against AP13 (Table S6). Of these, 21 and 44 genes were expressed at higher levels in VS16 and AP13, respectively (Table 5; Supplementary Table S6). Heat tolerance genes such as heat shock proteins (HSP) and chaperones were significantly expressed in both ecotypes. In VS16, 19 DnaJ-, and two DnaK-related HSPs participated in protein folding and post-transcriptional gene silencing were identified while in AP13, 36 DnaJ-related HSPs were expressed (Supplementary Table S6).

#### Flooding tolerance genes

Transcriptome analysis of VS16 and AP13 identified 183 differentially expressed transcripts related to flooding tolerance (Supplementary Table S7). Flooding tolerance genes were abundant in VS16 compared to AP13. Among these, 143 and 40 genes were abundantly expressed in VS16 and AP13, respectively (Table 6; Supplementary Table S7). The flooding tolerance genes identified in VS16 along with their function include: 31 expansin B2 precursor transcripts that are essential for plasticity in cell walls; seven adenine nucleotide alpha hydrolases that mediate nucleotide metabolism and transport; 13 leucine-rich repeat transmembrane protein kinases that regulate a wide variety of development and defense-related processes; five transducin family proteins that participate in phototransduction; and 17 xyloglucan endotransglucosylases that promote cell expansion. Flooding tolerance-related genes identified in AP13 with their function include: four adenine nucleotide alpha hydrolases that are implicated in leaf senescence; three leucine-rich repeat transmembrane protein kinases that are involved in developmental processes including cell proliferation and hormone perception; three RPA70-kDa subunit B that play a role in DNA replication and repair; and 4 carotenoid cleavage dioxygenases that participate in biosynthesis of apocarotenoids.

**Table 6:** List of up- and down-regulated flood and salinity-responsive genes in VS16 compared to AP13

Gene ID	Log2FC	Description
Flood-responsive genes		
Pavir.Da00154	8.93114	Xyloglucan endotransglucosylase/hydrolase 25; glycosyl hydrolases family 16, putative, expressed
Pavir.Ga00388	7.66341	Xyloglucan endotransglucosylase/hydrolase 9; glycosyl hydrolases family 16 protein, protein, expressed
Pavir.Ab02402	7.53719	Expansin B3; expansin precursor, putative, expressed
Pavir.Ca01085	7.03993	Leucine-rich repeat transmembrane protein kinase; leucine-rich repeat receptor protein kinase EXS precursor, putative, expressed
Pavir.J05963	7.0287	Leucine-rich repeat transmembrane protein kinase; leucine-rich repeat receptor protein kinase EXS precursor, putative, expressed
Pavir.J37973	6.79487	GRF zinc finger family protein
Pavir.Ea03672	6.52076	Adenine nucleotide alpha hydrolases-like superfamily protein; universal stress protein domain-containing protein, putative, expressed
Pavir.Gb00304	6.37009	Xyloglucan endotransglucosylase/hydrolase 9; glycosyl hydrolases family 16 protein, protein, expressed
Pavir.Ba00925	6.33641	Leucine-rich repeat transmembrane protein kinase; leucine-rich repeat receptor protein kinase EXS precursor, putative, expressed
Pavir.Bb02784	6.22624	Leucine-rich repeat transmembrane protein kinase; leucine-rich repeat receptor protein kinase EXS precursor, putative, expressed
Pavir.Ga02401	−3.71698	*O*-Acetylserine (thiol) lyase B; cysteine synthase, chloroplast/chromoplast precursor, putative, expressed
Pavir.Ab02771	−3.7392	Nine-cis-epoxycarotenoid dioxygenase 4; 9-cis-epoxycarotenoid dioxygenase 1, chloroplast precursor, putative, expressed
Pavir.Aa01044	−3.7416	Nine-cis-epoxycarotenoid dioxygenase 4; 9-cis-epoxycarotenoid dioxygenase 1, chloroplast precursor, putative, expressed
Pavir.J30088	−3.75305	Carotenoid cleavage dioxygenase 1; carotenoid cleavage dioxygenase, putative, expressed
Pavir.Ba03436	−3.85182	Leucine-rich repeat transmembrane protein kinase; SHR5-receptor-like kinase, putative, expressed
Pavir.Eb00451	−4.11109	Adenine nucleotide alpha hydrolases-like superfamily protein; universal stress protein domain-containing protein, putative, expressed
Pavir.J36211	−4.13895	Beta-amylase 5; beta-amylase, putative, expressed
Pavir.J32180	−4.96437	Universal stress protein domain-containing protein, putative, expressed
Pavir.J08037	−6.74544	RPA70-kDa subunit B; expressed protein
Pavir.J10011	−7.2897	Leucine-rich repeat transmembrane protein kinase; SHR5-receptor-like kinase, putative, expressed
Salinity-responsive genes		
Pavir.Ea00003	7.97093	Tonoplast intrinsic protein 4;1; aquaporin protein, putative, expressed
Pavir.Cb01832	6.85293	Tonoplast intrinsic protein 4;1; aquaporin protein, putative, expressed
Pavir.Ca00461	6.57124	Tonoplast intrinsic protein 4;1; aquaporin protein, putative, expressed
Pavir.Aa02820	5.95073	NOD26-like intrinsic protein 4;2; aquaporin protein, putative, expressed
Pavir.Ab01231	5.57162	NOD26-like intrinsic protein 1;2; aquaporin protein, putative, expressed
Pavir.J09715	5.01203	NOD26-like intrinsic protein 1;2; aquaporin protein, putative, expressed
Pavir.Ab02135	4.98671	Phenylalanine ammonia-lyase 4; phenylalanine ammonia-lyase, putative, expressed
Pavir.Ab02345	4.98358	PHE ammonia-lyase 1; phenylalanine ammonia-lyase, putative, expressed
Pavir.Ia02110	4.89046	NOD26-like intrinsic protein 5;1; aquaporin protein, putative, expressed
Pavir.Aa01274	4.82093	PHE ammonia-lyase 1; phenylalanine ammonia-lyase, putative, expressed
Pavir.Ha00406	−3.67129	General control non-repressible 5; ABC transporter, ATP-binding protein, putative, expressed
Pavir.Ab03307	−3.72565	ABC transporter family protein; white–brown complex homolog protein, putative, expressed
Pavir.J06912	−3.81225	General control non-repressible 5; ABC transporter, ATP-binding protein, putative, expressed
Pavir.Ab01904	−4.03494	ABC transporter family protein; multidrug resistance protein, putative, expressed
Pavir.Ga02654	−4.18423	Multidrug resistance-associated protein 14; ABC transporter family protein, putative, expressed
Pavir.Ga02259	−4.22223	Multidrug resistance-associated protein 14; ABC transporter, ATP-binding protein, putative, expressed
Pavir.J31685	−5.09275	General control non-repressible 5; ABC transporter, ATP-binding protein, putative, expressed
Pavir.Bb01841	−5.23059	Plasma membrane intrinsic protein 2; aquaporin protein, putative, expressed
Pavir.Ba01199	−5.25504	Plasma membrane intrinsic protein 3; aquaporin protein, putative, expressed
Pavir.Gb00641	−5.30131	PHE ammonia-lyase 1; phenylalanine ammonia-lyase, putative, expressed

#### Salinity tolerance genes

We identified 114 transcripts that affect salinity tolerance in VS16 and AP13 (Supplementary Table S8). VS16 has more salt tolerant genes as compared to AP13. Here we considered number of genes expressed at a set threshold of Log2FC > 2. Based on relative expression, VS16 has more number (76) of salt tolerant genes when compared to AP13 (38) (Table 6; Supplementary Table S8). The salinity tolerance genes identified in VS16 with their respective functions include: 18 ATP-binding cassette (ABC) transporter family proteins that are responsible for membrane transport; four transcripts for PAL that play a role in biosynthesis of polyphenol compounds such as flavonoids, phenylpropanoids, and lignin; five nuclear shuttle protein (NSP)-interacting kinases that are involved in defense against geminivirus; six tonoplast intrinsic proteins associated with vacuolar function; and six transcript homologs for plasma membrane intrinsic proteins that are involved in water transport. Similarly, the salinity tolerance genes identified in AP13 include 24 ABC transporter family proteins, two aldehyde dehydrogenases that catalyze oxidation, four PAL, two NOD26-like intrinsic proteins (NIPs) that facilitate exchange of metabolites, three NSP-interacting kinases, and three tonoplast intrinsic proteins.

### Biotic stress tolerance genes

#### Disease resistance genes

Of the 39 disease resistance genes identified in our analysis, four were up-regulated with at least a twofold change between VS16 and AP13 (Table 9; Supplementary Table S10). Different families of up-regulated disease-resistance genes identified included nucleotide-binding-APAF-1, R proteins, and CED (NB-ARC; 196), nucleotide-binding site–leucine-rich repeat (NBS-LRR; 82), leucine-rich repeat–nucleotide binding-APAF-1, R proteins, and CED (LRR-NB-ARC; 32), coiled-coil–nucleotide-binding site–leucine-rich repeat (CC-NBS-LRR; 21), leucine-rich repeat (LRR; 125), toll interleukin receptor–nucleotide-binding site–leucine-rich repeat (TIR-NBS-LRR class; 18), disease resistance-responsive (dirigent-like protein; 14), and ENHANCED DISEASE RESISTANCE 2 (3) (Table S10). Additionally, 43 other disease resistance-related genes that were differentially expressed between VS16 and AP13 switchgrass genotypes were identified. Of these, four genes were expressed at significantly higher levels in AP13 and their associated functions include: a protease inhibitor that prevents the breakdown of specific proteins, two bi-functional inhibitor proteins associated with protein coding, and a RING/U-box superfamily protein which participates in ubiquitin transfer of substrate proteins (Table S10). The remaining 39 genes were expressed at higher levels in VS16, among which 28 significantly expressed transcripts were bi-functional inhibitor proteins, two were soluble N-ethylmaleimide sensitive factor (NSF) attachment protein Receptor (SNARE) proteins associated with membranous vesicle fusion, five were lipid transfer-related proteins, and four were plant syntaxin proteins involved in bacterial resistance.

#### Transporters in stress

We identified 229 transporters involved in stress by comparing VS16 against AP13 (Table 7; Supplementary Table S9). AP13 has more transporters involved in stress than VS16. Out of these, 103 and 126 transcripts were significantly expressed in VS16 and AP13, respectively (Table 7). The transporters identified in VS16 with their implicated functions include: 18 ATPases associated with diverse cellular activities (AAA)-type family proteins that are involved in cell-cycle regulation, 12 pleiotropic drug resistance proteins that are associated with the hydrolysis of ATP to transfer various substrates across cellular membranes, and six terpenoid cyclases that are responsible for the synthesis of molecules such as antibacterial and antifungal agents. Similarly, the transporters identified in AP13 with their associated functions include: 29 ABC-type transporter family proteins that play an important role in homeostasis of small ions and macromolecules, eight photosystem II reaction center proteins that are involved in capturing energy by direct absorption of light by chlorophyll, four photosystem II stability/assembly factors involved in photosynthesis, six pleiotropic drug resistance proteins involved in membrane transport, and four protein kinase superfamily proteins that are associated with the protein phosphorylation. In this study, we identified ABC-specific transporter family proteins in VS16 and AP13 (Table 8). Peptide transporter (PTR; 22), cation/proton exchanger (CAX; 11), potassium transporter (KUP; eight), oligo peptide transporter (OPT; seven), sulfate transporter (SULTR; four), and magnesium transporter (MRS; three) are highly expressed in AP13. On the other hand, phosphate transporter (PHO; 14), potassium channel (AKT/KAT; six), Fe (2+) transporter protein (IRT; four), ammonium transporter (AMT; four), K (+) efflux antiporter (KEA; four), sodium transporter (HKT; four), cation/H (+) antiporter (CHX; three), magnesium/proton exchanger (MHX; three) are significantly enriched in VS16.

**Table 7:** Significant up- and down-regulated transporters in stress in VS16 compared to AP13

Gene ID	Log2FC	Description
Pavir.Ia03597	8.75484	Glutamine-dependent asparagine synthase 1; asparagine synthetase, putative, expressed
Pavir.J25709	7.52548	ATP-binding cassette subfamily B19; multidrug resistance protein, putative, expressed
Pavir.Ea00336	7.2087	Pleiotropic drug resistance 4; pleiotropic drug resistance protein, putative, expressed
Pavir.Ib03577	6.85242	White–brown complex homolog protein 11; white–brown complex homolog protein 11, putative, expressed
Pavir.J25945	6.57426	Pleiotropic drug resistance 4; pleiotropic drug resistance protein, putative, expressed
Pavir.Eb00416	6.50819	Pleiotropic drug resistance 4; pleiotropic drug resistance protein, putative, expressed
Pavir.Ga01466	6.32578	ATP-binding cassette subfamily B19; multidrug resistance protein, putative, expressed
Pavir.J34236	6.2879	ATP-binding cassette subfamily B19; multidrug resistance protein, putative, expressed
Pavir.Fb02214	6.03411	ATP-binding cassette subfamily B1; multidrug resistance protein, putative, expressed
Pavir.J19576	5.83792	ABC-2 type transporter family protein; white–brown complex homolog protein, putative, expressed
Pavir.Ib00360	−4.94843	ABC-2 type transporter family protein; ABC-2 type transporter domain-containing protein, expressed
Pavir.J31685	−5.09275	General control non-repressible 5; ABC transporter, ATP-binding protein, putative, expressed
Pavir.J13221	−5.24601	Pleiotropic drug resistance 12; pleiotropic drug resistance protein, putative, expressed
Pavir.J13221	−5.24601	Pleiotropic drug resistance 12; pleiotropic drug resistance protein, putative, expressed
Pavir.J06421	−5.57434	ABC-2 type transporter family protein; ABC-2 type transporter domain-containing protein, expressed
Pavir.Fb01115	−5.66159	ABC-2 type transporter family protein; ABC-2 type transporter domain-containing protein, expressed
Pavir.Ib00245	−5.74326	ABC-2 type transporter family protein; ABC-2 type transporter domain-containing protein, expressed
Pavir.Aa00159	−6.37424	Non-intrinsic ABC protein 12; white–brown complex homolog protein, putative, expressed
Pavir.Bb02969	−6.86662	Pleiotropic drug resistance 11; pleiotropic drug resistance protein, putative, expressed
Pavir.J09830	−6.87181	Photosystem II reaction center PSB28 protein; photosystem II reaction center PSB28 protein, chloroplast precursor, putative, expressed

**Table 8:** Important up-regulated metal transporter family proteins in VS16 and AP13 genotypes

Identified transporters from genome mining genes	>Twofold up-regulation	
	VS16	AP13
Peptide transporter (PTR)	12	22
Phosphate transporter (PHO)	14	5
Cation/proton exchanger (CAX)	3	11
Potassium transporter (KUP)	6	8
Oligopeptide transporter (OPT)	0	7
Nitrate transporter (NRT)	5	6
Putative peptide/nitrate transporters (PNT)	5	6
Potassium channel (AKT/KAT)	6	4
Sulfate transporter (SULTR)	2	4
Fe(2 +) transporter protein (IRT)	4	1
Ammonium transporter (AMT)	4	0
K(+) efflux antiporter (KEA)	4	0
Sodium transporter (HKT)	4	0
Magnesium transporter (MRS)	1	3
Cation/H(+) antiporter (CHX)	3	0
Magnesium/proton exchanger (MHX)	3	0
Ca-activated outward-rectifying K channel (TPK)	0	3

#### Other important genes

Another set of interesting genes identified in both VS16 and AP13 genotypes were RNA-binding proteins implicated in post-transcriptional gene regulation that include Mei2-like, pumilio, and RNA recognition motif (RRM) domain-containing proteins, while the decamping enzyme was only identified in VS16 (Supplementary Table S11).

### RT-PCR validation of RNA-Seq analysis

**Fig. 3:**
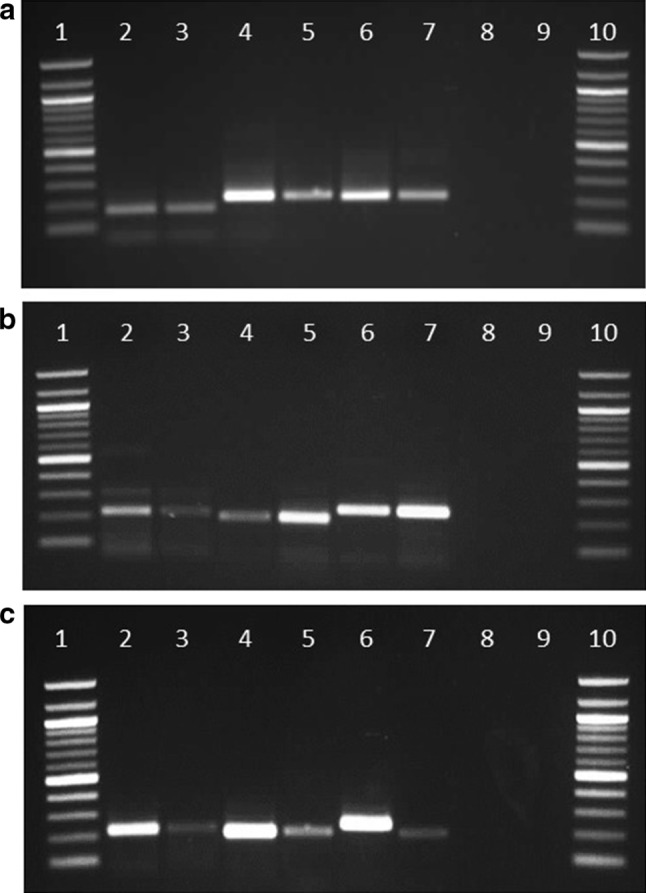
Reverse transcriptase-PCR assays of heat-, drought-, and salinity-responsive genes in switchgrass ecotypes, AP13 and VS16. *Lane 1* and *10* 100 bp ladder; *Lanes 2, 4, 6* AP13 and *3, 5, 7* VS16; *Lane 8* No reverse transcriptase (RT); *Lane* 9 H_2_O. **a** Heat stress-responsive genes: Heat shock protein 20 (Hsp20) (*lanes 2–3*), Heat shock protein 90 (Hsp90) (*lanes 4–5*), and Chaperone DnaJ-domain superfamily protein (*lanes 6–7*). **b** Drought stress-responsive genes: Delta tonoplast integral protein (*2–3*), Plasma membrane intrinsic protein 3 (*4–5*), and NOD-26 intrinsic protein 5;1 (*6–7*). **c** Salinity stress-responsive genes: Vacuolar H+-ATPase Subunit E isoform 3 (*2–3*), Leucine-rice repeat protein kinase family protein BRASSINOSTEROID INSENSITIVE 1 precursor (*4–5*), and Aldehyde dehydrogenase 2C4 (*6–7*). We used *cons7* as an internal control for normalization. Note: Unusual posttranscriptional processing such as deletion of 5′ exon material or insertion of exogenous gene sequences was seen to result in differential transcriptional products in in cereal crops (Niu et al. 2007). We suggest that the distinct genetic differences between the upland VS16 genotype and lowland AP13 genotype used in this study could be a reason for differential product sizes of the aldehyde dehydrogenase gene

The heat-responsive genes selected in this study include heat shock protein 20 (hsp20), hsp90, and chaperone Dan-domain superfamily protein and the expression of these genes were validated by amplifying them using cDNA. The superfamily protein was highly expressed in AP13 compared to VS16. However, there was no change in hsp20 expression between ecotypes (Fig. 3a). The drought stress-responsive genes validated in this study include delta tonoplast integral protein, plasma intrinsic protein (PIP) 3, and NOD-26 intrinsic protein (NIP) 5;1. The PIP and NIP were highly expressed in VS16 when compared to AP13. The expression of delta tonoplast integral protein was slightly higher in AP13 than VS16 (Fig. 3b). The salinity stress-responsive genes, vacuolar H^+^ ATPase subunit E isoform 3, leucine-rich repeat protein kinase family protein BRASSINOSTEROID INSENSITIVE 1 precursor, and aldehyde dehydrogenase 2C4, all showed higher expression in AP13 when compared to VS16 (Fig. 3c). Differential banding pattern of aldehyde dehydrogenase between the two genotypes is seen in our study. This has been seen in other crops such as cereals (Niu et al. 2007) and was suggested to be due to unusual posttranscriptional processing such as deletion of 5′ exon material or insertion of exogenous gene sequences resulting in differential transcriptional products and in response to stress conditions. Given that the two switchgrass genotypes are from two distinct ecotypes, upland and lowland, it is possible that there are inherent genetic differences between these plants.

### Quantitative PCR validation of RNA-Seq analysis

**Fig. 4:**
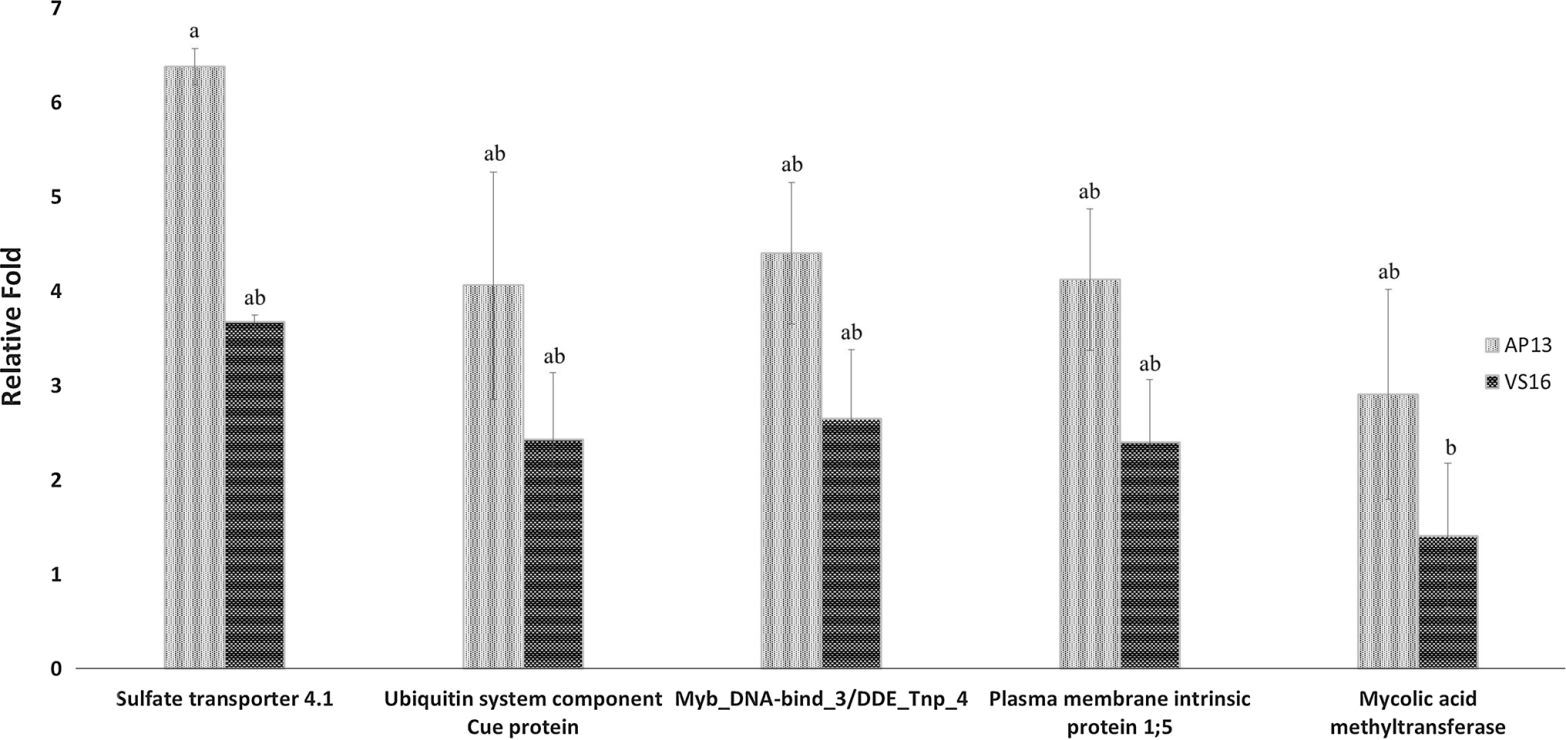
Differential expression of genes between AP13 and VS16. The *x*-axis shows different genes: Sulfate transporter 4.1, Ubiquitin system component Cue protein, Myb/SANT-like DNA-binding domain (Myb_DNA-bind_3)//DDE superfamily endonuclease (DDE_Tnp_4), Plasma membrane intrinsic protein 1;5, Mycolic acid methyl transferase and *y*-axis shows relative fold change value (Log2FC). Cons7 was used for the normalization of gene expression. *Letters* (*a, b*, and *c*) indicate statistically significant (*P* < 0.05) groups showing differential expression between AP13 and VS16 ecotypes for selected genes. The qPCR results from three technical replicates were subjected to ANOVA by using Minitab 17 software

To quantitatively validate differentially expressed transcripts from RNA-Seq analysis, we performed qRT-PCR on five genes from both AP13 and VS16. Genes selected for qRT-PCR were those involved in bio-synthesis of secondary metabolites, plant–pathogen interaction, a plant transposon protein, an aquaporin transporter protein (Pavir.J37677), and a putative methyl transferase. These genes were selected based on their significance in differential expression between two genotypes using RNA-Seq analysis. The significant (*P* < 0.05) variation in the relative expression of all five genes indicated that each of these genes were highly expressed in AP13 than in VS16 (Fig. 4).

## Discussion

RNA sequencing followed by bioinformatics analysis and experimental validation as presented here provide comprehensive transcriptome profiles for two contrasting genotypes, AP13 and VS16, belonging to two switchgrass ecotypes, lowland and upland, respectively. The transcriptome data generated in this experiment can also be used as references in future genomics and transcriptomic studies. The majority of the transcripts annotated here belonged to cellular and metabolic processes based on Agrigo analysis (Fig. 1). Data mining and assigning gene ontology terms to different gene classes helped in the identification of several important transcript homologs that belonged to pathway-related genes, transcription factors, transporters and genes involved in both biotic and abiotic stresses.

### Genes associated with photosynthesis

Though several pathways with differentially expressed genes were identified, we highlight the pathways that are directly or indirectly linked with photosynthetic efficiency. Our analysis focused on genes associated with C4 photosynthesis. The majority of the genes in this pathway were abundantly expressed in AP13 when compared to VS16 supporting the fact that specifically AP13 is photosynthetically more active and could have impact on higher biomass yield than VS16 (Serba et al. 2013). Our finding is in accordance with previous reports that lowland ecotypes of switchgrass are more productive especially in biomass yield (Alexopoulou et al. 2008). Interestingly, carbonic anhydrase involved in carbon-fixing metabolism in C4 plants was abundantly expressed in AP13 (twofold increase) when compared to VS16 supporting reports on potential use of lowland ecotypes for biofuel production. The stomatal conductance was seen to be reduced in carbonic anhydrase-deficient mutants that were treated with carbonyl sulfide (COS) in grasses (Stimler et al. 2012). The second important enzyme in the C4 pathway is PEPC, which was also highly expressed in AP13 (fourfold higher when compared to VS16). Another important enzyme identified here was pyruvate orthophosphate dikinase (PPDK) and it was only expressed significantly in VS16 but not in AP13. An increase in PPDK has been reported in cold tolerant species of sugar cane indicating the ability of PPDK content as a factor for cold tolerance (Halther et al. 2015). VS16 is able to tolerate cold temperatures better than AP13 and this may be the reason for the significant increase of PPDK in VS16 compared to AP13. In our study, Pyruvate orthophosphate dikinase regulatory protein (PDRP) was significantly expressed only in AP13, supporting its role in higher biomass production (Chastain et al. 2011). PDRP activates PPDK by reversible phosphorylation of an active threonine (Thr) residue.

### Genes associated with photorespiration

Photorespiration is a physiological process, which has significant influence in biomass accumulation. In plants, eight key enzymes regulate photorespiration and they are primarily located in chloroplast, mitochondrion, and peroxisome (Chen et al. 2014). All eight of these enzymes were identified in this study. The role of dicarboxylate transporters in photorespiration was elaborately discussed in several plant species in ammonia assimilation (Buchner et al. 2015). Here we identified three dicarboxylate transporter homologs that were highly expressed in AP13. This finding may imply that the process of nitrogen recycling occurs more in AP13 than VS16 (Rao et al. 2016). Further, we found that AP13 has more glutamate synthase (GS) compared to VS16. The number of significantly enriched GS genes in VS16 and AP13 was one and four, respectively. GS are the enzymes responsible for conversion of inorganic nitrogen to glutamine and glutamate during ammonium assimilation and they donate nitrogen during biosynthesis of amino acids and to compounds including chlorophyll, hormones and secondary metabolite products. GS is also involved in the transport of toxic metabolites and these attributes of AP13, may indicate that it undergoes more plant metabolic processes compared to VS16 (Oliveira et al. 2001). Another important enzyme involved in photorespiration is hydroxypyruvate reductase (HPR). In a recent study, using over expression and RNAi lines of HPR1, it was found that HPR1 gene activity is important for photorespiratory metabolite flux in rice (Bauwe et al. 2010). In this study, we identified one significantly enriched HPR gene in AP13 which means it can assimilate more CO_2_ and thus produce more biomass. Further, RuBisCO is a predominant CO_2_ fixing enzyme in plants. This study identified 11 significantly enriched RuBisCO genes in AP13. Furthermore, serine hydroxyl methyltransferases (SHM) not only play an important role in carbon metabolism and photorespiration but also in controlling the cell damage/death induced by biotic and abiotic stresses (Dellero et al. 2015). The level of expression of SHM was two folds more in VS16 when compared to AP13.

### Genes associated with phenylpropanoid pathway

Previous studies showed relationship between lignin biosynthesis and saccharification/ethanol yield (Chen and Dixon 2007; Gressel 2008; Leplé et al. 2007; Dien et al. 2006). We identified phenylpropanoid pathway genes that play a role in lignin biosynthesis. Though there are more than 25 genes associated with phenylpropanoid metabolism, tyrosine is assumed to be the starting point in plants such as grasses (Moreno et al. 2005). More upstream genes in phenylpropanoid pathway are expressed in VS16 than in AP13 but the last gene (CAD) are equally expressed in both. Down-regulation of upstream enzymes, HCT, C3H and COMT in the lignin biosynthesis pathway resulted in lower lignin content in switchgrass (Escamilla-Trevino et al. 2014; Rao et al. 2013). In transgenic switchgrass cv. Alamo, suppression of 4CL and CAD genes showed reduction in lignin content and increased saccharification efficiency (Xu et al. 2011; Fu et al. 2011). Previous report suggested that phenylpropanoid pathway genes, PAL, 4-CL, CAD, COMT and CCoAOMT were abundantly expressed in lowland cultivar Kanlow than upland cultivar Summer (Palmer et al. 2014). Contrarily, this study identified ferulic acid, CCR, PAL, 4CL, CCoAMOT, HCT, and C4H genes uniquely expressed in the upland cultivar, VS16. This supports that VS16 (upland) can produce more lignin than the lowland ecotypes which affects downstream processes such as digestion of cellulose and hemicellulose and thus reducing the use of upland ecotypes as biofuel stock compared to lowland ecotypes (Bhandari et al. 2014).

### Transcription factors in switchgrass

We evaluated both the ecotypes for differentially expressed transcription factors that regulate various physiological processes including plant growth and development (Ramachandran et al. 1994). However, their level of expression varied between the ecotypes. The differentially expressed transcription factors were more in VS16 (428) when compared with AP13 (197), suggesting ecotype-specific variation in gene expression. The TFs that were commonly identified in the two ecotypes of switchgrass include DREB, AP2 domain-containing, C2H2, MYB, NAC, NAM, Integument, zip, and MADS box families of TFs. Several TFs identified here were in common with the TFs reported in other grasses such as *Oryza sativa*, *Zeal mays*, *Sorghum bicolor*, *Saccharin ophidiarium* and *Brachypodium distachyon* (Yilmaz et al. 2009). The significant TFs identified here overlapped with recent studies (Li et al. 2013; Bhatia and Bosch 2014) that include: AUX/IAA transcriptional regulator family (8), FAR1-related sequence (6), beta-8 tubulin (5), beta-6 tubulin (5), and TRAF-like family protein (4). In addition to these, we also identified TFs, homeodomain-like superfamily protein (22), basic leucine zipper (33) transcription factor family protein (19), C2H2-type zinc finger family protein (17), mitochondrial transcription termination factor family protein (15), integrase-type DNA-binding superfamily protein (13), RING/FYVE/PHD zinc finger superfamily protein (13), GRAS family transcription factor (12), and BTB-POZ and MATH domain (12).

Previous reports suggested that several TFs play a vital role in regulating the gene expression in plant stress responses and some are discussed here. The down-regulation of WRKY family transcription factors resulted in increased lignin content in cell walls that enhanced the biomass content in *Medicago sativa* (Gallego-Giraldo et al. 2015). WRKY-mediated transcriptional regulation in flooding tolerance has been reported in switchgrass (Barney et al. 2009). Transgenic switchgrass lines that overexpress the MYB4 TF showed higher lignocellulosic content (Shen et al. 2013). Here, we identified bHLH, MYB, WRKY, NAM, AP2 domain-containing, BWDNA-binding domain-containing, heat shock factor (HSF), NAC, MADS box, and Aintegumenta (Ant) families of TFs that had suggested roles in regulating lingocellulosic content in lateral meristems of switchgrass (Li et al. 2013). Recently, higher expression of WRKY and NAC genes independently involved in pathogen responses, and senescing of flag leaves of switchgrass have been reported (Serba et al. 2015). Majority of the TFs identified in this study overlapped with the abiotic stress-responsive TFs that have been reported in rice (Todaka et al. 2012).

### Genes associated with biotic and abiotic stress responses

We identified a suit of biotic and abiotic stress-responsive gene transcripts in the two switchgrass genotypes. Interestingly, none of the drought-responsive transcripts identified here overlapped with previous studies in switchgrass but overlapped with the transcripts from other grasses such as rice and sorghum (Pandey and Shukla 2015; Sharma et al. 2006). Drought-responsive genes uniquely identified here include plasma membrane intrinsic protein (9), NOD26-like intrinsic protein (8), tonoplast intrinsic protein (3), and delta tonoplast integral protein (2). The majority of the drought-responsive genes identified here were classified as aquaporins and NOD26-like intrinsic proteins (Fetter et al. 2004). Plant aquaporins play an important role in drought tolerance by facilitating water and small solute transport across the cell membrane and thus regulate plant growth and development. The expression of aquaporins varies with spatial and temporal expression and with environmental conditions (Gomez et al. 2009). An increase in expression of aquaporin-dependent plasma membrane intrinsic proteins (PIP) was evident in abiotic stress responses including drought, high-salt, low-temperature, and heavy-metal stress (Jang et al. 2004).

Heat stress adversely affects membrane and cytoskeleton structures by modulating gene expression; to overcome this, plants develop a considerable amount of tolerance by reprograming their transcriptomes. However, relative heat tolerance varies between the upland (VS16) and lowland (AP13) switchgrass ecotypes and their transcriptomes were analyzed for heat tolerant genes. Upland ecotypes are more adapted to colder climate and lowland ecotypes in hot and humid regions. In our study, we identified more than 20 DnaJ and DnaK transcripts that were associated with heat tolerance by directly or indirectly binding to hsp70, which is in concurrence with a previous study (Mayer and Bukau 2005). The majority of the heat tolerant genes such as DnaJ-domain superfamily proteins (20), DnaJ heat shock N-terminal domain-containing proteins (10), DnaJ heat shock family protein (9), heat shock cognate protein 70-1 (3), and hsp70 (3) identified here overlapped with a previous study in switchgrass (Li et al. 2013). Conversely, three heat tolerant genes uniquely identified in this dataset include DnaJ/Hsp40 cysteine-rich domain superfamily proteins (9), chloroplast hsp70-1 (2), and DnaJ domain-containing protein (1). Comparative transcriptome profiling showed differential gene expression related to heat shock proteins, including hsp90 in *Pyropia* *yezoensis* (Sun et al. 2015). In wheat and barley, the genome-wide identification revealed 27 and 13 newly identified hsps (Pandey et al. 2015).

The majority of the flooding related genes identified here belonged to cell division and cell wall loosening that include cyclin, expansin, replicon protein A2, xyloglucan endotransglucosylase family protein, and growth-regulatory factor 5. In an earlier report, flooding induced shoot elongation by regulating apoplastic acidification, cell wall loosening, cell division, and starch breakdown by employing at least three hormones viz., ethylene, ABA and GA in *Rumex* *palustris* (Voesenek et al. 2003). AP13 (lowland) can grow well in flood plains. Three days of flooding did not impact switchgrass survival in Oklahoma (Personal communication, M.C.Saha).

The relative salinity tolerance varies among the ecotypes; lowland has 26X and 15X more sodium than upland at maturity and after senescence, respectively (Yang et al. 2009) and here we compared the transcript profiles of VS16 and AP13 to determine the relative salinity tolerance between the two genotypes of switchgrass. Salinity stress-responsive genes identified here were in concurrence with an earlier study in switchgrass (Liu et al. 2014) and included: ABC transporter family proteins, multidrug resistance-associated proteins, general control non-repressible, non-intrinsic ABC proteins, NSP-interacting kinases, and P-glycoproteins. Importantly, the unique salt stress-responsive genes identified in this study were aldehyde dehydrogenase, PHE ammonia-lyase, plasma membrane intrinsic protein, NOD26-like intrinsic protein, and tonoplast intrinsic proteins, which have been reported in rice and sorghum (Liu et al. 2009). In this study, we identified 17 xyloglucan endotransglucosylase/hydrolase genes. Here we validated expression of three salinity-responsive genes using RT-PCR. The five most important plant transporter families that have been implicated in biotic and abiotic stress responses included: ATP-binding cassette (ABC), multidrug and toxic compound exporters (MATE), major facilitator superfamily (MFS), small multidrug resistance (SMR), and resistance-nodulation-division proteins (RND) (Peng et al. 2011). However, ABC is the largest transporter family protein found in all living organisms. Here, we identified 33 and 27 ABC transporter family proteins in VS16 and AP13, respectively (Table 8; Supplementary Table S9). We found two subfamilies of ABC transporters, ABCG (19) and ABCC (1) in AP13. Whole transcriptome analysis in rice revealed several differentially expressed genes involved in drought signaling pathways under Cd stress (Oono et al. 2014). We identified 18 AAA-type ATPase family proteins in AP13. There were significantly expressed transporters identified uniquely in VS16 which are; ammonium transporter (AMT), cation/H (+) antiporter (CHX), copper transporter (COPT), sodium transporter (HKT), K (+) efflux antiporter (KEA), magnesium/proton exchanger (MHX), molybdate transporter (MOT), metal tolerance protein (MTP), nicotianamine synthase 1 (NAS), nitrate excretion transporter (NAXT), sodium/hydrogen exchanger (NHX), metal transporter (NRAMP), and zinc transporter (ZIP).

### Genes associated with disease resistance

Several NBS domain-containing R proteins were identified in AP13 and VS16. Additionally, disease resistance genes, bi-functional inhibitor/lipid transfer protein/seed storage 2S albumin superfamily proteins and azelaic acid-induced transcripts were also identified (Serba et al. 2015). However, plant syntaxin proteins were abundantly and uniquely identified in this study between the two ecotypes. The lipid transfer proteins (LTPs) primarily transfer lipids between the monolayers, and their role in biotic and abiotic stresses has been reported in Arabidopsis (Safi et al. 2015). In this study, 33 significantly enriched LTPs were identified and overlapped with the LTPs that have been reported in the high-tillering genotype (VS16) of switchgrass (Li et al. 2013).

Vesicular transport proteins, such as syntaxins and signal recognition particles, play an important role in auxin signaling, cytokinesis, and disease resistance (Wang et al. 2013b). Vesicular transport proteins, syntaxin and signal recognition particles have been up-regulated in high-tillering genotype, VS16 (Li et al. 2013). Similarly, we identified 6 syntaxin proteins and 2 signal recognition particles in VS16.

Pathogenesis-related genes including chitinase have been shown to be important for defense against plant pathogens. We identified 10 chitinase genes, among which, chitinase 16, chitinase A, and basic chitinase were highly expressed (Log2FC > 2) in both AP13 and VS16 (Supplementary Table S11). About fourfold higher expression of these chitinases were observed in VS16 than in AP13.

In switchgrass, cytochrome P450 has been implicated in heat stress and eleven different variants of cytochrome P450 have been reported (Li et al. 2013). Here we identified 85 and 46 cytochrome P450 transcript homologs in VS16 and AP13, respectively (Table 9; Supplementary Table S11) and found that 43 thioredoxins were induced in heat stress in AP13. Both genotypes showed elevated levels of thioredoxin in response to oxidative stress that resulted from heat stress (Li et al. 2013). We also identified two calcineurin b genes in the two ecotypes that were up-regulated in heat stress. In *Arabidopsis*, calcineurin B-like gene was preferentially expressed in stems and roots, and its expression was up-regulated in response to drought, cold and wounding stresses (Kudla et al. 1999). At least 75 up-regulated heat stress-responsive calmodulin superfamily proteins were expressed in VS16 (Table 9; Supplementary Table S11). In a recent study, the genome-wide transcriptome analysis of soybean identified various Hsfs in drought, low temperature, and ABA stress responses (Li et al. 2014). The genotype AP13 exhibited 11 up-regulated Hsfs and also identified two calcium Atpase. Genes encoding various protective proteins such as late embryogenesis abundant proteins, GSTs, Mn SOD, glutathione gamma-glutamyl cysteinyl transferase important for GSH biosynthesis, peroxiredoxin, thioredoxin, and PMSR were also identified in this study.

**Table 9:** Significant up- and down-regulated genes involved in disease resistance as well as other stress-related genes in VS16 compared to AP13

Gene ID	Log2FC	Description
Genes in disease resistance		
Pavir.Ga01060	8.02562	Extensin-like protein; LTPL121—Protease inhibitor/seed storage/LTP family protein precursor, putative, expressed
Pavir.Ga01059	7.94306	Bifunctional inhibitor/lipid transfer protein/seed storage 2S albumin superfamily protein; LTPL122 Protease inhibitor/seed storage/LTP family protein precursor, expressed
Pavir.J14567	7.88782	Bifunctional inhibitor/lipid transfer protein/seed storage 2S albumin superfamily protein; LTPL128 Protease inhibitor/seed storage/LTP family protein precursor, expressed
Pavir.Ba03729	7.46549	Bifunctional inhibitor/lipid transfer protein/seed storage 2S albumin superfamily protein; LTPL100 Protease inhibitor/seed storage/LTP family protein precursor, expressed
Pavir.Aa01606	−3.28972	Azelaic acid-induced 1; LTPL114—Protease inhibitor/seed storage/LTP family protein precursor, expressed
Pavir.J12674	−3.95316	RING/U-box superfamily protein; LTPL9—Protease inhibitor/seed storage/LTP family protein precursor, expressed
Other important genes		
Pavir.J06588	8.42896	Cytochrome P450, family 71, subfamily B, polypeptide 37; cytochrome P450, putative, expressed
Pavir.Ib03890	7.81	Cytochrome P450, family 71, subfamily B, polypeptide 2; cytochrome P450, putative, expressed
Pavir.Db00359	7.77268	Cytochrome P450, family 77, subfamily B, polypeptide 1; cytochrome P450, putative, expressed
Pavir.Ea02184	7.01089	Tetratricopeptide-repeat thioredoxin-like 1; TTL1, putative, expressed
Pavir.Ga01037	6.96059	Cytochrome P450, family 86, subfamily A, polypeptide 4; cytochrome P450, putative, expressed
Pavir.Eb01893	6.4813	Tetratricopeptide-repeat thioredoxin-like 1; TTL1, putative, expressed
Pavir.J23416	6.38279	Tetratricopeptide-repeat thioredoxin-like 1; TTL1, putative, expressed
Pavir.Ab01476	6.34185	Cytochrome P450, family 76, subfamily C, polypeptide 4; cytochrome P450, putative, expressed
Pavir.J09753	6.33701	Cytochrome P450, family 72, subfamily A, polypeptide 15; cytochrome P450 72A1, putative, expressed
Pavir.Ga01931	6.1496	Cytochrome P450, family 77, subfamily A, polypeptide 4; cytochrome P450, putative, expressed
Pavir.Ib03534	6.07324	Cytochrome P450, family 86, subfamily B, polypeptide 1; cytochrome P450, putative, expressed
Pavir.Ab01784	6.07018	Plant calmodulin-binding protein-related; expressed protein
Pavir.Bb03711	6.04474	CBL-interacting protein kinase 3; CAMK_KIN1/SNF1/Nim1_like.32—CAMK includes calcium/calmodulin dependent protein kinases, expressed
Pavir.J30784	4.62782	Calcium-binding EF-hand family protein; calcineurin B, putative, expressed
Pavir.Eb00202	4.60844	Tetratricopeptide-repeat thioredoxin-like 3; TTL1, putative, expressed
Pavir.Ea00158	4.5998	Tetratricopeptide-repeat thioredoxin-like 3; TTL1, putative, expressed
Pavir.Ea03756	4.422	Chitinase A
Pavir.Ib00193	4.36714	Chitinase 16
Pavir.J34564	4.25235	Basic Chitinase
Pavir.Ea02316	−3.4228	Chitinase A
Pavir.Ab00798	−4.0587	Chitinase 4
Pavir.Ab00798	−4.0587	Basic Chitinase
Pavir.J24864	−4.16364	Chitinase 2
Pavir.Ib00871	−4.30914	Cytochrome P450, family 76, subfamily C, polypeptide 1; cytochrome P450, putative, expressed
Pavir.J10796	−4.59569	Cytochrome P450, family 709, subfamily B, polypeptide 2; cytochrome P450 72A1, putative, expressed
Pavir.Ia00714	−4.69108	Thioredoxin superfamily protein; expressed protein
Pavir.Ha00715	−4.81133	Cytochrome P450, family 716, subfamily A, polypeptide 1; cytochrome P450, putative, expressed
Pavir.Da01689	−4.90659	Cytochrome P450 superfamily protein; cytochrome P450, putative, expressed
Pavir.Ia00715	−5.18689	Thioredoxin superfamily protein; expressed protein
Pavir.Hb00116	−5.18783	Calmodulin-binding protein; calmodulin-binding protein, putative, expressed
Pavir.Aa02817	−5.32122	NADPH-dependent thioredoxin reductase C; bifunctional thioredoxin reductase/thioredoxin, putative, expressed
Pavir.Ib03354	−5.35319	Cytochrome P450 superfamily protein; cytochrome P450, putative, expressed
Pavir.Ia03284	−5.41344	Cytochrome P450, family 709, subfamily B, polypeptide 3; cytochrome P450 72A1, putative, expressed
Pavir.Cb01800	−5.42569	Thioredoxin superfamily protein; thioredoxin, putative, expressed
Pavir.J01458	−5.81263	Ataurora3; CAMK_CAMK_like_Aur_like.2—CAMK includes calcium/calmodulin dependent protein kinases, expressed
Pavir.J40555	−7.06753	Cytochrome P450, family 71, subfamily B, polypeptide 23; cytochrome P450, putative, expressed
Pavir.Ia01888	−7.24124	Cytochrome P450 superfamily protein; cytochrome P450, putative, expressed
Pavir.J36245	−9.84065	Thioredoxin superfamily protein; thioredoxin, putative, expressed

### RT-PCR and qRT-PCR validation of RNA-Seq

The reasons for selecting stress-responsive genes were: (1) Switchgrass ecotypes have distinct geographic niches, thus their morphology is largely influenced by environmental conditions; (2) lowland ecotypes are relatively more tolerant to pests and disease (biotic stress) than upland ecotypes; (3) upland ecotypes are comparatively more cold tolerant (abiotic stress) than lowland ecotypes (Sanderson et al. 1996). Lowland genotypes have high sodium content than their upland counterparts (Yang et al. 2009). Three highly and significantly enriched heat-responsive genes including hsp20, hsp90, and chaperone DnaJ-domain superfamily proteins were qualitatively validated their expression by amplifying cDNA from both genotypes. RNA-Seq analysis revealed that hsp20 was uniformly expressed between AP13 and VS16 as confirmed by using RT-PCR. We observed changes in hsp90 and chaperone DnaJ-domain superfamily protein expression between AP13 and VS16. Both genes were highly expressed in AP13 compared to VS16 (Fig. 3a). Concurrently, we validated three drought-responsive proteins including delta tonoplast integral protein (TIP), plasma membrane intrinsic protein 3 (PIP3), and NOD-26 intrinsic protein 5 (NIP). PIP 3 and NOD-26 intrinsic protein 5 were highly expressed in VS16 when compared to AP13 (Fig. 3b). The expression of delta tonoplast integral protein was slightly higher in AP13 than in VS16. Also, we validated three salinity-responsive genes that include: vacuolar H^+^-ATPase subunit E isoform, leucine-rich repeat protein kinase family protein BRASSINOSTEROID INSENSITIVE 1 precursor, and aldehyde dehydrogenase 2C4. All three genes showed higher expression in AP13 when compared to VS16 (Fig. 3c).

Furthermore, five differentially expressed genes from RNA-Seq analyses, secondary metabolite biosynthesis, plant pathogen interaction, plant transposon protein, membrane transporter protein and putative methyltransferase were selected and validated by qRT-PCR. The expression of monolignol biosynthesis enzymes that produce secondary metabolites which have diverse functional roles upon modification have been validated through qRT-PCR in *Populus* *trichocarpa* (Shi et al. 2009). Similarly, we validated the expression of cytochrome P450 monooxygenease homolog in switchgrass (pavir.Ia03311.1). The expression of this gene was higher in AP13 when compared to VS16 as predicted from RNA-Seq analyses. The expression of pathogenesis-related gene (Pavir.Ia00685.1) was higher in AP13 (lowland ecotype) when compared to VS16 (upland ecotype) as identified from RNA-Seq analyses. In another study, the relative expression of four transposable proteins was evaluated in leaf and sheath samples of rice (Zheng et al. 2013). Here, we compared the relative expression of transposable proteins between AP13 and VS16 using qRT-PCR and identified similar trend in expression. We validated the expression of an aquaporin transporter protein (Pavir.J37677) and its expression was higher in AP13 when compared to VS16. The qRT-PCR analysis revealed that the expression of putative methyltransferase was higher in AP13 than VS16 but its function was still unclear. However, the single nucleotide resolution genome-wide methylation maps specific for AP13 and VS16 may aid in understanding the epigenetic landscape of these two contrasting ecotypes.

## Conclusion

Comprehensive transcriptome profiling in both lowland and upland switchgrass ecotypes (AP13 and VS16), as analyzed here provided the identification of differentially expressed genes and transcription factors that are associated with biomass yield, disease resistance, and abiotic stresses such as, heat, drought, flood, and salinity. The lowland genotype, AP13, showed higher expression of biomass-related genes while the upland genotype, VS16, showed upregulation of some stress-related genes. This study also identified biomass production and quality associated key enzymes in phenylpropanoid, C4-photosynthesis, and photorespiratory pathways. The other major group of genes identified here belonged to plant stress and disease resistance. We validated selected genes from transcriptome analysis using RT-PCR and qRT-PCR and identified similar trends in expressions. Further studies taking into account a broader array of ecotypes and different plant tissues sampled at vegetative and reproductive stages of plant development will be useful, as these will broaden the datasets of this current work to match phenotypic variations that reflect this prolific native species of North America.

### **Author contribution statement**

VA helped design and plan the study, conducted the experiments, and wrote the manuscript; MCS helped with design of the study, and helped edit and write the manuscript; JT helped analyze the data and edited the manuscript, VRS helped analyze the data, and write the manuscript, KPB helped analyze the data, and edited the manuscript, EF helped analyze the data, and write the manuscript, RKH organized and edited the manuscript, and VK designed and planned the study, and helped edit and write the manuscript.

## Electronic supplementary material

Below is the link to the electronic supplementary material.

Supplementary material 1 (DOCX 133 kb)
