# Supplementary material for: Comparative transcriptome profiling of upland (VS16) and lowland (AP13) ecotypes of switchgrass
Source: Plant Cell Rep. 2016 Nov 3;36(1):129–50. doi: 10.1007/s00299-016-2065-0 (PMC5206262; doi:10.1007/s00299-016-2065-0)
Supplement: Supplementary file 1 — Supplementary material 1 (DOCX 133 kb) [file 299_2016_2065_MOESM1_ESM.docx]

**Supplementary Table 1: Primers utilized for Reverse-Transcriptase PCR analysis in switchgrass**

| Gene ID | Description | Primer Sequences | | | | | Product Size | | | Highly expressed in RNA-Seq | |  |  |  |  |  |  |  |  |  |  |  |  |
| --- | --- | --- | --- | --- | --- | --- | --- | --- | --- | --- | --- | --- | --- | --- | --- | --- | --- | --- | --- | --- | --- | --- | --- |
|  |  |  | | | | |  | | | AP13 | VS16 |  |  |  |  |  |  |  |  |  |  |  |  |
| Heat-responsive genes | | |  | | |  | | | | | | |  |  | | |  | | |  |  |  |  |
| Pavir.J08073 | Heat-shock protein 20 | 5’-GACGAGGTGAAGGTGATGGT-3’ 3’-ACCTTGCTCTTGTCGCACTC-5’ | | | | | 164 | | | Yes |  |  |  |  |  |  |  |  |  |  |  |  |  |
| Pavir.Fa00597 | Heat-shock protein 90 | 5’-GCTGTGGCGGATAGTAGCTC-3’ 3’-TGGGGAATGACACAAACTGA-5’ | | | | | 175 | | | Yes |  |  |  |  |  |  |  |  |  |  |  |  |  |
| Pavir.Ca02433 | Chaperone DnaJ-domain superfamily protein | 5’-TATGGGAGGCTTGGAGTCAC-3’ 3’-GCCAGACATATCGCTCAGGT-5’ | | | | | 223 | | |  | Yes |  |  |  |  |  |  |  |  |  |  |  |  |
| Drought-responsive genes | | | | |  | | | |  | | | | | | |  | | |  | | |  | |
| Pavir.Db01217 | Delta tonoplast integral protein | 5’-GGCGTCGTGATGGAGATAAT-3’ 3’-TCCAGATGTTGGTGAAGTCG-5’ | | | | | 223 | | | Yes |  |  |  |  |  |  |  |  |  |  |  |  |  |
| Pavir.Ba01199 | Plasma mem instrinsic protein 3 | 5’-AGGAGGATGTGAGCATCGAG-3’ 3’-GTCTGCAGGTTGTTGGACTG-5’ | | | | | 205 | | |  | Yes |  |  |  |  |  |  |  |  |  |  |  |  |
| Pavir.Ab02995 | NOD26-like intrinsic protein 5 | 5’-AAGAAGGTCGTCTCGGAGGT-3’ 3’-AGAAGGGGACCTGAATCCAT-5’ | | | | | 241 | | |  | Yes |  |  |  |  |  |  |  |  |  |  |  |  |
| Salinity-responsive genes | | | |  | | | |  | | | | | | |  | | |  | | |  | |  |
| Pavir.Eb00174 | vacuolar H+-ATPase subunit E isoform 3 | 5’-ATCAGGCAGGAATTTGATCG-3’ 3’-GTTTGGTGATCTCGGCTGAT-5’ | | | | | 176 | | | Yes |  |  |  |  |  |  |  |  |  |  |  |  |  |
| Pavir.Db00464 | Leucine-rich repeat protein kinase family protein BRASSINOSTEROID INSENSITIVE 1 precursor | 5’-ATCTCACTGTTTGCGCCTCT-3’ 3’-CACAGGCAACGTATCACACC-5’ | | | | | 239 | | | Yes |  |  |  |  |  |  |  |  |  |  |  |  |  |
| Pavir.Ea01601 | aldehyde dehydrogenase 2C4 | 5’-ATTGAGCCCACCATCTTCAC-3’ 3’-CAGGGTCGAAGGCAAAGTAG-5’ | | | | | 244 | | |  | Yes |  |  |  |  |  |  |  |  |  |  |  |  |

**Supplementary Table 2: Primers utilized for quantitative Real-Time PCR validation in switchgrass**

| Gene ID | Description | Primer sequences |
| --- | --- | --- |
| Pavir.J01761 | Sulfate transporter 4.1 | F-GGACGTGTCATCGTCAGACA |
|  |  | R-TTGACCAAGCTCACCACAA |
| Pavir.Ea02144 | Ubiquitin system component Cue protein | F-GCAATAGGGGTGGCAACAATG |
|  |  | R-GAAAACCGCACAGAAGGCAA |
| Pavir.Da02128 | Myb/SANT-like DNA-binding domain//DDE superfamily endonuclease | F-CACGATTGTGGAAGCGCAAG |
|  |  | R-GTCAACATGGATGGTCGCCT |
| Pavir.J37677 | Plasma membrane intrinsic protein 1;5 | F-CCATGATCCTGGCCTACGG |
|  |  | R-GTTCATGACGTAGGGGTTGC |
| Pavir.J13507 | Mycolic acid methyl transferase | F-ACGCTCTTGACCTTCACCAG |
|  |  | R-GGATTCGGTGTTCACCCTGT |
| Pavir.Ia01092 | *Cons7* (Reference) | F-AACTTCGTCTACGGCCAGTC |
|  |  | R-GTGGCATACTTGGAACCCTT |

**Supplementary Table 3: List of KEGG pathways with number genes identified for each pathway in VS16 and AP13**

| KEGG ID | Description | Annotation | |
| --- | --- | --- | --- |
|  |  | VS16 | AP13 |
| ko01100 | Metabolic pathways | 344 | 352 |
| ko01110 | Biosynthesis of secondary metabolites | 183 | 178 |
| ko01130 | Biosynthesis of antibiotics | 75 | 100 |
| ko03010 | Ribosome | 27 | 78 |
| ko01120 | Microbial metabolism in diverse environments | 62 | 62 |
| ko01230 | Biosynthesis of amino acids | 47 | 59 |
| ko00230 | Purine metabolism | 23 | 43 |
| ko01200 | Carbon metabolism | 40 | 40 |
| ko00240 | Pyrimidine metabolism | 19 | 38 |
| ko04141 | Protein processing in endoplasmic reticulum | 12 | 38 |
| ko00190 | Oxidative phosphorylation | 32 | 37 |
| ko05016 | Huntington's disease | 20 | 37 |
| ko00195 | Photosynthesis | 37 | 2 |
| ko03040 | Spliceosome | 34 | 24 |
| ko03008 | Ribosome biogenesis in eukaryotes | 13 | 32 |
| ko03013 | RNA transport | 20 | 31 |
| ko05010 | Alzheimer's disease | 16 | 30 |
| ko04075 | Plant hormone signal transduction | 22 | 28 |
| ko05169 | Epstein-Barr virus infection | 17 | 28 |
| ko05012 | Parkinson's disease | 22 | 27 |
| ko04110 | Cell cycle | 8 | 27 |
| ko04111 | Cell cycle-yeast | 6 | 26 |
| ko00520 | Amino sugar and nucleotide sugar metabolism | 15 | 25 |
| ko00860 | Porphyrin and chlorophyll metabolism | 22 | 4 |
| ko04120 | Ubiquitin mediated proteolysis | 19 | 21 |
| ko04932 | Non-alcoholic fatty liver disease (NAFLD) | 13 | 21 |
| ko03030 | DNA replication | 5 | 21 |
| ko04113 | Meiosis-yeast | 6 | 20 |
| ko00510 | N-Glycan biosynthesis | 4 | 20 |
| ko00330 | Arginine and proline metabolism | 18 | 19 |
| ko00970 | Aminoacyl-tRNA biosynthesis | 19 | 11 |
| ko04142 | Lysosome | 7 | 19 |
| ko04145 | Phagosome | 5 | 19 |
| ko03050 | Proteasome | 3 | 19 |
| ko03018 | RNA degredation | 18 | 18 |
| ko00010 | Glycolysis/Gluconeogenesis | 17 | 18 |
| ko00564 | Glycerophospholipid metabolism | 18 | 13 |
| ko05166 | HTLV-I infection | 11 | 18 |
| ko00270 | Cysteine and methionine metabolism | 17 | 16 |
| ko01210 | 2-Oxocarboxylic acid metabolism | 13 | 17 |
| ko03015 | mRNA surveillance pathway | 12 | 17 |
| ko01212 | Fatty acid metabolism | 11 | 17 |
| ko00710 | Carbon fixation in photosynthetic organisms | 17 | 6 |
| ko05203 | Viral carcinogenesis | 16 | 13 |
| ko03420 | Nucleotide excision repair | 12 | 16 |
| ko00260 | Glycine, serine and threonine metabolism | 11 | 16 |
| ko00561 | Glycerolipid metabolism | 16 | 7 |
| ko04114 | Oocyte meiosis | 11 | 15 |
| ko04721 | Synaptic vesicle cycle | 3 | 15 |
| ko00513 | Various types of N-glycan biosynthesis | 2 | 15 |
| ko04146 | Peroxisome | 13 | 14 |
| ko00500 | Starch and sucrose metabolism | 14 | 12 |
| ko00900 | Terpenoid backbone biosynthesis | 14 | 12 |
| ko05200 | Pathways in cancer | 8 | 14 |
| ko03440 | Homologous recombination | 6 | 14 |
| ko00130 | Ubiquinone and other terpenoid-quinone biosynthesis | 14 | 5 |
| ko00630 | Glyoxylate and dicarboxylate metabolism | 13 | 11 |
| ko00250 | Alanine, aspartate and glutamate metabolism | 9 | 13 |
| ko00480 | Glutathione metabolism | 9 | 13 |
| ko03410 | Base excision repair | 8 | 13 |
| ko00410 | beta-Alanine metabolism | 6 | 13 |
| ko00100 | Steroid biosynthesis | 5 | 13 |
| ko00770 | Pantothenate and CoA biosynthesis | 3 | 13 |
| ko05110 | Vibrio cholerae infection | 3 | 13 |
| ko00906 | Carotenoid biosynthesis | 13 | 2 |
| ko00400 | Phenylalanine, tyrosine and tryptophan biosynthesis | 12 | 11 |
| ko04626 | Plant -pathogen interaction | 12 | 11 |
| ko04144 | Endocytosis | 11 | 12 |
| ko00620 | Pyruvate metabolism | 11 | 12 |
| ko00020 | Citrate cycle (TCA cycle) | 8 | 12 |
| ko00051 | Fructose and mannose metabolism | 7 | 12 |
| ko04151 | PI3K-Akt signaling pathway | 7 | 12 |
| ko03060 | Protein export | 7 | 12 |
| ko03460 | Fanconi anemia pathway | 3 | 12 |
| ko00196 | Photosynthesis-antenna proteins | 12 | 1 |
| ko00680 | Methane metabolism | 11 | 11 |
| ko00280 | Valine, leucine and isoleucine degradation | 11 | 11 |
| ko03020 | RNA polymerase | 7 | 11 |
| ko03430 | Mismatch repair | 6 | 11 |
| ko04071 | Sphingolipid signaling pathway | 5 | 11 |
| ko04130 | SNARE interactions in vesicular transport | 3 | 11 |
| ko00030 | Pentose phophate pathway | 10 | 8 |
| ko00940 | Phenylpropanoid biosynthesis | 7 | 10 |
| ko04914 | Progesterone-mediated oocyte maturation | 7 | 10 |
| ko00071 | Fatty acid degredation | 5 | 10 |
| ko04712 | Circadian rhythm-plant | 10 | 4 |
| ko00562 | Inositol phosphate metabolism | 8 | 9 |
| ko03022 | Basal transcription factors | 9 | 7 |
| ko00910 | Nitrogen metabolism | 9 | 6 |
| ko05230 | Central carbon metabolism in cancer | 6 | 9 |
| ko00920 | Sulfur metabolism | 9 | 5 |
| ko00053 | Ascorbate and aldarate metabolism | 5 | 9 |
| ko04068 | FoxO signaling pathway | 4 | 9 |
| ko04810 | Regulation of actin cytoskeleton | 4 | 9 |
| ko05134 | Legionellosis | 3 | 9 |
| ko05120 | Epithelial cell signaling in Helicobacter pylori infection | 1 | 9 |
| ko05323 | Rheumatoid arthritis | 1 | 9 |
| ko00052 | Galactose metabolism | 8 | 8 |
| ko00360 | Phenylalanine metabolism | 8 | 8 |
| ko04070 | Phosphatidylinositol signaling system | 8 | 7 |
| ko00941 | Flavonoid biosynthesis | 7 | 8 |
| ko04910 | Insulin signaling pathway | 7 | 8 |
| ko05152 | Tuberculosis | 7 | 8 |
| ko04066 | HIF-1 signaling pathway | 6 | 8 |
| ko04922 | Glucagon signaling pathway | 8 | 5 |
| ko05034 | Alcoholism | 5 | 8 |
| ko00061 | Fatty acid biosynthesis | 5 | 8 |
| ko04728 | Dopaminergic synapse | 4 | 8 |
| ko00300 | Lysine biosynthesis | 4 | 8 |
| ko04115 | p53 signaling pathway | 4 | 8 |
| ko00983 | Drug metabolism-other enzymes | 3 | 8 |
| ko00640 | Propanoate metabolism | 1 | 8 |
| ko04966 | Collecting duct acid secretion | 0 | 8 |
| ko04152 | AMPK signaling pathway | 7 | 7 |
| ko04261 | Adrenergic signaling in cardiomyocytes | 5 | 7 |
| ko05168 | Herpes simplex infection | 5 | 7 |
| ko00760 | Nicotinate and nicotinamide metabolism | 7 | 4 |
| ko04623 | Cytosolic DNA-sensing pathway | 4 | 7 |
| ko00450 | Selenocompound metabolism | 4 | 7 |
| ko00290 | Valine, leucine and isoleucine biosynthesis | 4 | 7 |
| ko00650 | Butanoate metabolism | 3 | 7 |
| ko04666 | Fc gamma R-mediated phagocytosis | 3 | 7 |
| ko04390 | Hippo signaling pathway | 2 | 7 |
| ko00720 | Carbon fixation pathways in prokaryotes | 0 | 7 |
| ko04260 | Cardiac muscle contraction | 6 | 6 |
| ko01040 | Biosynthesis of unsaturated fatty acids | 6 | 5 |
| ko00460 | Cyanoamino acid metabolism | 6 | 5 |
| ko04919 | Thyroid hormone signaling pathway | 5 | 6 |
| ko04915 | Estrogen signaling pathway | 6 | 4 |
| ko00350 | Tyrosine metabolism | 6 | 4 |
| ko04310 | Wnt signaling pathway | 6 | 4 |
| ko00340 | Histidine metabolism | 4 | 6 |
| ko00261 | Monobactam biosynthesis | 4 | 6 |
| ko00380 | Tryptophan metabolism | 4 | 6 |
| ko02020 | Two-component system | 4 | 6 |
| ko04122 | Sulfur relay system | 6 | 3 |
| ko00563 | Glycosylphosphatidylinositol (GPI)-anchor biosynthesis | 3 | 6 |
| ko04722 | Neurotrophin signaling pathway | 3 | 6 |
| ko04612 | Antigen processing and presentation | 2 | 6 |
| ko05160 | Hepatitis C | 2 | 6 |
| ko00310 | Lysine degradation | 1 | 6 |
| ko05322 | Systemic lupus erythematosus | 1 | 6 |
| ko05130 | Pathogenic Escherichia coli infection | 0 | 6 |
| ko04024 | cAMP signaling pathway | 5 | 5 |
| ko05231 | Choline metabolism in cancer | 5 | 5 |
| ko05161 | Hepatitis B | 5 | 5 |
| ko05164 | Influenza A | 5 | 5 |
| ko00565 | Ether lipid metabolism | 5 | 4 |
| ko04014 | Ras signaling pathway | 5 | 4 |
| ko05206 | MicroRNAs in cancer | 4 | 5 |
| ko00600 | Sphingolipid metabolism | 4 | 5 |
| ko05145 | Toxoplasmosis | 4 | 5 |
| ko00790 | Folate biosynthesis | 5 | 3 |
| ko04720 | Long-term potentiation | 5 | 3 |
| ko00780 | Biotin metabolism | 3 | 5 |
| ko00040 | Pentose and glucuronate interconversions | 3 | 5 |
| ko05215 | Prostate cancer | 3 | 5 |
| ko05205 | Proteoglycans in cancer | 3 | 5 |
| ko04010 | MAPK signaling pathway | 5 | 2 |
| ko04510 | Focal adhesion | 2 | 5 |
| ko04391 | Hippo signaling pathway-fly | 2 | 5 |
| ko00945 | Stilbenoid, diarylheptanoid and gingerol biosynthesis | 2 | 5 |
| ko05210 | Colorectal cancer | 1 | 5 |
| ko05213 | Endometrial cancer | 1 | 5 |
| ko05132 | Salmonella infection | 1 | 5 |
| ko00592 | alpha-Linolenic acid metabolism | 5 | 0 |
| ko03070 | Bacterial secretion system | 5 | 0 |
| ko04724 | Glutamatergic synapse | 5 | 0 |
| ko00905 | Brassinosteroid biosynthesis | 0 | 5 |
| ko04022 | cGMP-PKG signaling pathway | 4 | 4 |
| ko04020 | Calcium signaling pathway | 4 | 3 |
| ko00982 | Drug metabolism-cytochrome P450 | 4 | 3 |
| ko04912 | GnRH signaling pathway | 4 | 3 |
| ko04916 | Melanogenesis | 4 | 3 |
| ko04921 | Oxytocin signaling pathway | 4 | 3 |
| ko04530 | Tight junction | 4 | 3 |
| ko00062 | Fatty acid elongation | 3 | 4 |
| ko04727 | GABAergic synapse | 3 | 4 |
| ko04540 | Gap junction | 3 | 4 |
| ko00670 | One carbon pool by folate | 3 | 4 |
| ko03320 | PPAR signaling pathway | 3 | 4 |
| ko00960 | Tropane, piperidine and pyridine alkaloid biosynthesis | 4 | 2 |
| ko05142 | Chagas disease (American trypanosomiasis) | 2 | 4 |
| ko04961 | Endocrine and other factor-regulated calcium reabsorption | 2 | 4 |
| ko05162 | Measles | 2 | 4 |
| ko04150 | mTOR signaling pathway | 2 | 4 |
| ko05131 | Shigellosis | 2 | 4 |
| ko00730 | Thiamine metabolism | 4 | 1 |
| ko05100 | Bacterial invasion of epithelial cells | 1 | 4 |
| ko04340 | Hedgehog signaling pathway | 1 | 4 |
| ko05222 | Small cell lung cancer | 1 | 4 |
| ko04724 | Gultamatergic synapse | 0 | 4 |
| ko00625 | Chloroalkane and chloroalkene degradation | 3 | 3 |
| ko04713 | Circadian entrainment | 3 | 3 |
| ko01220 | Degradation of aromatic compounds | 3 | 3 |
| ko00980 | Metabolism of xenobiotics by cytochrome P450 | 3 | 3 |
| ko00830 | Retinol metabolism | 3 | 3 |
| ko04350 | TGF-beta signaling pathway | 3 | 3 |
| ko04918 | Thyroid hormone synthesis | 3 | 3 |
| ko04270 | Vascular smooth mucle contraction | 3 | 3 |
| ko05031 | Amphetamine addiction | 3 | 2 |
| ko00660 | C5-Branched dibasic acid metabolism | 3 | 2 |
| ko04621 | NOD-like receptor signaling pathway | 3 | 2 |
| ko00511 | Other glycan degradation | 3 | 2 |
| ko05020 | Prion diseases | 3 | 2 |
| ko05211 | Renal cell carcinoma | 3 | 2 |
| ko00750 | Vitamin B6 metabolism | 3 | 2 |
| ko04360 | Axon guidance | 2 | 3 |
| ko04112 | Cell cycle-Caulobacter | 2 | 3 |
| ko04062 | Chemokine signaling pathway | 2 | 3 |
| ko05214 | Glioma | 2 | 3 |
| ko00903 | Limonene and pinene degradation | 2 | 3 |
| ko05212 | Pancreatic cancer | 2 | 3 |
| ko05133 | Pertussis | 2 | 3 |
| ko04660 | T cell receptor signaling pathway | 2 | 3 |
| ko04930 | Type II diabetes mellitus | 2 | 3 |
| ko04520 | Adherens junction | 3 | 1 |
| ko05014 | Amyotrophic lateral sclerosis (ALS) | 3 | 1 |
| ko00950 | Isoquinoline alkaloid biosynthesis | 3 | 1 |
| ko04330 | Notch signaling pathway | 3 | 1 |
| ko00531 | Glycosaminoglycan degradation | 1 | 3 |
| ko03450 | Non-homologous end-joining | 1 | 3 |
| ko05223 | Non-small cell lung cancer | 1 | 3 |
| ko00430 | Taurine and hypotaurine metabolism | 1 | 3 |
| ko04962 | Vasopressin-regulated water reabsorption | 1 | 3 |
| ko04975 | Fat digestion and absorption | 3 | 0 |
| ko00440 | Phosphonate and phosphinate metabolism | 3 | 0 |
| ko00362 | Benzoate degradation | 0 | 3 |

**Supplementary Table 4: List of transcription factors identified in VS16 compared to AP13**

| Gene ID | log2FC | Description |
| --- | --- | --- |
| Pavir.Ca02370 | 8.11458 | Myb domain protein 46; myb-like DNA-binding domain containing protein, putative, expressed |
| Pavir.J19884 | 8.03805 | BTB-POZ and MATH domain 1; MBTB32 - Bric-a-Brac, Tramtrack, Broad Complex BTB domain with Meprin and TRAF Homology MATH domain, expressed |
| Pavir.Aa00318 | 7.61192 | Nuclear factor Y, subunit A5; nuclear transcription factor Y subunit, putative, expressed |
| Pavir.Ga01785 | 7.56188 | Xylem NAC domain 1; no apical meristem protein, putative, expressed |
| Pavir.J26815 | 7.50209 | G-box binding factor 6; bZIP transcription factor domain containing protein, expressed |
| Pavir.Fa00854 | 7.48631 | General regulatory factor 7; 14-3-3 protein, putative, expressed |
| Pavir.Fa01426 | 6.98614 | Basic leucine-zipper 7; bZIP transcription factor domain containing protein, expressed |
| Pavir.Ia02808 | 6.84614 | GATA transcription factor 4; GATA zinc finger domain containing protein, expressed |
| Pavir.J37973 | 6.79487 | GRF zinc finger family protein |
| Pavir.J18139 | 6.69668 | Myb domain protein 86; MYB family transcription factor, putative, expressed |
| Pavir.J20716 | 6.67407 | Myb domain protein 86; MYB family transcription factor, putative, expressed |
| Pavir.Ab02456 | 6.63975 | WRKY family transcription factor; WRKY36, expressed |
| Pavir.Ea03909 | 6.532 | Squamosa promoter-binding protein-like (SBP domain) transcription factor family protein; OsSPL2 - SBP-box gene family member, expressed |
| Pavir.Ib02277 | 6.45395 | GATA transcription factor 4; GATA zinc finger domain containing protein, expressed |
| Pavir.Ib00051 | 6.41665 | NAC domain containing protein 28; no apical meristem protein, putative, expressed |
| Pavir.Ca02736 | 6.30961 | Indoleacetic acid-induced protein 16; OsIAA30 - Auxin-responsive Aux/IAA gene family member, expressed |
| Pavir.Fa00877 | 6.17817 | Myb domain protein 106; MYB family transcription factor, putative, expressed |
| Pavir.Aa00510 | 6.17714 | Myb domain protein 93; MYB family transcription factor, putative, expressed |
| Pavir.J11826 | 6.08826 | Myb domain protein 48; MYB family transcription factor, putative, expressed |
| Pavir.Ea01893 | 6.07724 | C2H2-like zinc finger protein; ZOS1-10 - C2H2 zinc finger protein, expressed |
| Pavir.Bb03711 | 6.04474 | CBL-interacting protein kinase 3; CAMK_KIN1/SNF1/Nim1_like.32 - CAMK includes calcium/calmodulin depedent protein kinases, expressed |
| Pavir.Aa00661 | 6.03556 | Immunoglobulin E-set superfamily protein; rho GDP-dissociation inhibitor 1, putative, expressed |
| Pavir.Ia01234 | 6.00202 | AUX/IAA transcriptional regulator family protein; OsIAA12 - Auxin-responsive Aux/IAA gene family member, expressed |
| Pavir.J06112 | 5.96879 | AUX/IAA transcriptional regulator family protein; OsIAA12 - Auxin-responsive Aux/IAA gene family member, expressed |
| Pavir.Ha00788 | 5.9099 | Basic helix-loop-helix (bHLH) DNA-binding superfamily protein; BHLH transcription factor, putative, expressed |
| Pavir.Ab01026 | 5.83986 | HMG (high mobility group) box protein; HMG1/2, putative, expressed |
| Pavir.Cb01647 | 5.68984 | Protein of unknown function (DUF630 and DUF632); BZIP protein, putative, expressed |
| Pavir.J03230 | 5.58547 | Myb domain protein 61; myb-like DNA-binding domain containing protein, putative, expressed |
| Pavir.J14631 | 5.5302 | Xylem NAC domain 1; no apical meristem protein, putative, expressed |
| Pavir.J29561 | 5.523 | Myb domain protein 103; myb-like DNA-binding domain containing protein, putative, expressed |
| Pavir.Ib00206 | 5.5229 | Beta HLH protein 93; BHLH transcription factor, putative, expressed |
| Pavir.Eb00031 | 5.5179 | AP2/B3-like transcriptional factor family protein; B3 DNA binding domain containing protein, expressed |
| Pavir.J36384 | 5.51401 | Mediator complex, subunit Med7; mediator of RNA polymerase II transcription subunit 7, putative, expressed |
| Pavir.Hb01247 | 5.49333 | Phytochrome-associated protein 1; OsIAA27 - Auxin-responsive Aux/IAA gene family member, expressed |
| Pavir.J24721 | 5.48338 | Heat shock cognate protein 70-1; DnaK family protein, putative, expressed |
| Pavir.J37128 | 5.47838 | BTB-POZ and MATH domain 1; MBTB51 - Bric-a-Brac, Tramtrack, Broad Complex BTB domain with Meprin and TRAF Homology MATH domain, expressed |
| Pavir.J01483 | 5.45789 | LOB domain-containing protein 40; DUF260 domain containing protein, putative, expressed |
| Pavir.J05229 | 5.45684 | SET domain-containing protein; SET domain containing protein, expressed |
| Pavir.Hb00825 | 5.40278 | Disease resistance protein (CC-NBS-LRR class) family; stripe rust resistance protein Yr10, putative, expressed |
| Pavir.J06478 | 5.36402 | SSXT family protein; GRF-interacting factor 1, putative, expressed |
| Pavir.J32047 | 5.36182 | Tubulin beta 8; tubulin/FtsZ domain containing protein, putative, expressed |
| Pavir.Gb00655 | 5.32849 | NAC domain containing protein 74; no apical meristem protein, putative, expressed |
| Pavir.Eb03102 | 5.28624 | GATA transcription factor 9; GATA zinc finger domain containing protein, expressed |
| Pavir.J11763 | 5.2749 | Growth-regulating factor 1; growth-regulating factor, putative, expressed |
| Pavir.Hb00031 | 5.24758 | Zinc-finger protein 10; ZOS7-01 - C2H2 zinc finger protein, expressed |
| Pavir.Gb02388 | 5.18841 | C2H2-like zinc finger protein; ZOS4-01 - C2H2 zinc finger protein, expressed |
| Pavir.J06554 | 5.15616 | Myb domain protein 61; myb-related protein Hv33, putative, expressed |
| Pavir.J15086 | 5.15034 | RING/FYVE/PHD zinc finger superfamily protein; zinc finger, C3HC4 type domain containing protein, expressed |
| Pavir.Eb01168 | 5.14696 | Beta-6 tubulin; tubulin/FtsZ domain containing protein, putative, expressed |
| Pavir.J00491 | 5.13276 | SAP domain containing protein, expressed |
| Pavir.J38363 | 5.1318 | Homeodomain-like superfamily protein; Myb transcription factor, putative, expressed |
| Pavir.J30990 | 5.12462 | Myb domain protein 4; MYB family transcription factor, putative, expressed |
| Pavir.J04979 | 5.1002 | Heat shock transcription factor C1; HSF-type DNA-binding domain containing protein, expressed |
| Pavir.Cb01523 | 5.08805 | RING/FYVE/PHD zinc finger superfamily protein; zinc finger, C3HC4 type domain containing protein, expressed |
| Pavir.J04678 | 5.04912 | Myb domain protein 54; MYB family transcription factor, putative, expressed |
| Pavir.J10145 | 5.03263 | Mini zinc finger 2; ZF-HD protein dimerisation region containing protein, expressed |
| Pavir.J03475 | 4.99625 | TCP family transcription factor 4; TCP family transcription factor, putative, expressed |
| Pavir.J04610 | 4.99383 | Indoleacetic acid-induced protein 16; OsIAA30 - Auxin-responsive Aux/IAA gene family member, expressed |
| Pavir.Eb02718 | 4.96715 | NAC domain containing protein 73; no apical meristem protein, putative, expressed |
| Pavir.Ca02380 | 4.94938 | GATA type zinc finger transcription factor family protein; OsWLIM1 - LIM domain protein, putative actin-binding protein and transcription factor, expressed |
| Pavir.Fb00364 | 4.9337 | Myb domain protein 103; myb-like DNA-binding domain containing protein, putative, expressed |
| Pavir.Ib03976 | 4.84119 | NAC domain containing protein 58; No apical meristem protein, putative, expressed |
| Pavir.J19088 | 4.81688 | Myb domain protein 30; myb-like DNA-binding domain containing protein, putative, expressed |
| Pavir.Ib03247 | 4.80141 | TRAF-like family protein; MATH domain containing protein, expressed |
| Pavir.J33965 | 4.80041 | K-box region and MADS-box transcription factor family protein; OsMADS4 - MADS-box family gene with MIKCc type-box, expressed |
| Pavir.J07596 | 4.75899 | HMG (high mobility group) box protein; HMG1/2, putative, expressed |
| Pavir.Aa00144 | 4.74802 | NAC domain containing protein 74; no apical meristem protein, putative, expressed |
| Pavir.Ga00239 | 4.73259 | OBF binding protein 1; dof zinc finger domain containing protein, putative, expressed |
| Pavir.Aa03359 | 4.7206 | Tubulin beta 8; tubulin/FtsZ domain containing protein, putative, expressed |
| Pavir.J01753 | 4.7174 | GATA type zinc finger transcription factor family protein; OsWLIM1 - LIM domain protein, putative actin-binding protein and transcription factor, expressed |
| Pavir.Ea00087 | 4.71084 | Homeodomain-like protein; MYB family transcription factor, putative, expressed |
| Pavir.Ib03996 | 4.70266 | Indoleacetic acid-induced protein 16; OsIAA30 - Auxin-responsive Aux/IAA gene family member, expressed |
| Pavir.Aa01258 | 4.69663 | TCP family transcription factor; TCP family transcription factor, putative, expressed |
| Pavir.Eb01219 | 4.69221 | Myb domain protein 108; MYB family transcription factor, putative, expressed |
| Pavir.Ga00724 | 4.68585 | Growth-regulating factor 5; growth-regulating factor, putative, expressed |
| Pavir.J07835 | 4.67063 | NAC (No Apical Meristem) domain transcriptional regulator superfamily protein; no apical meristem protein, putative, expressed |
| Pavir.J39511 | 4.64561 | WRKY DNA-binding protein 38; WRKY64, expressed |
| Pavir.Gb01850 | 4.61168 | NAC domain containing protein 100; no apical meristem protein, putative, expressed |
| Pavir.Ba01379 | 4.60447 | High mobility group B3; HMG1/2, putative, expressed |
| Pavir.J04092 | 4.59002 | Myb-like HTH transcriptional regulator family protein; MYB family transcription factor, putative, expressed |
| Pavir.Ea03187 | 4.54813 | Beta-6 tubulin; tubulin/FtsZ domain containing protein, putative, expressed |
| Pavir.Ca01617 | 4.54633 | RING/FYVE/PHD zinc finger superfamily protein; zinc finger, C3HC4 type domain containing protein, expressed |
| Pavir.J20698 | 4.53913 | NAC (No Apical Meristem) domain transcriptional regulator superfamily protein; no apical meristem protein, putative, expressed |
| Pavir.J13418 | 4.51947 | RING/FYVE/PHD zinc finger superfamily protein; zinc finger, C3HC4 type domain containing protein, expressed |
| Pavir.Ea00433 | 4.48429 | AGAMOUS-like 80; OsMADS88 - MADS-box family gene with M-gamma type-box, expressed |
| Pavir.J31344 | 4.40583 | Myb domain protein 61; MYB family transcription factor, putative, expressed |
| Pavir.J14351 | 4.37755 | Mini zinc finger 1; ZF-HD protein dimerisation region containing protein, expressed |
| Pavir.J32835 | 4.37337 | Integrase-type DNA-binding superfamily protein; AP2 domain containing protein, expressed |
| Pavir.Ba00257 | 4.3716 | CBL-interacting protein kinase 3; CAMK_KIN1/SNF1/Nim1_like.32 - CAMK includes calcium/calmodulin depedent protein kinases, expressed |
| Pavir.Eb03208 | 4.35267 | TCP family transcription factor 4; slTCP3, putative, expressed |
| Pavir.Da00789 | 4.30675 | NAC domain containing protein 75; no apical meristem protein, putative, expressed |
| Pavir.Gb00885 | 4.26379 | Integrase-type DNA-binding superfamily protein; AP2 domain containing protein, expressed |
| Pavir.J34139 | 4.25421 | Integrase-type DNA-binding superfamily protein; AP2 domain containing protein, expressed |
| Pavir.J03242 | 4.20589 | BTB-POZ and MATH domain 1; MBTB10 - Bric-a-Brac, Tramtrack, Broad Complex BTB domain with Meprin and TRAF Homology MATH domain, expressed |
| Pavir.J13756 | 4.1933 | Indoleacetic acid-induced protein 16; OsIAA13 - Auxin-responsive Aux/IAA gene family member, expressed |
| Pavir.Ba00112 | 4.18736 | Protein with RING/U-box and TRAF-like domains; seven in absentia protein family domain containing protein, expressed |
| Pavir.Ba00556 | 4.17029 | Brassinosteroid signalling positive regulator (BZR1) family protein; BES1/BZR1 homolog protein, putative, expressed |
| Pavir.Eb03507 | 4.15562 | BZIP transcription factor family protein; transcription factor, putative, expressed |
| Pavir.Ia04450 | 4.15533 | Beta HLH protein 93; BHLH transcription factor, putative, expressed |
| Pavir.J15676 | 4.13633 | Basic helix-loop-helix (bHLH) DNA-binding superfamily protein; BHLH transcription factor, putative, expressed |
| Pavir.Fb00508 | 4.10855 | BED zinc finger ;hAT family dimerisation domain; ZOS9-05 - C2H2 zinc finger protein, expressed |
| Pavir.Ba01001 | 4.10104 | SNF7 family protein; SNF7 domain containing protein, putative, expressed |
| Pavir.Eb00326 | 4.10028 | RING/FYVE/PHD zinc finger superfamily protein; PHD-finger domain containing protein, putative, expressed |
| Pavir.J25463 | 4.09049 | Beta HLH protein 93; BHLH transcription factor, putative, expressed |
| Pavir.J00016 | 4.08087 | K-box region and MADS-box transcription factor family protein; OsMADS4 - MADS-box family gene with MIKCc type-box, expressed |
| Pavir.Fb01563 | 4.07499 | Myb domain protein 20; MYB family transcription factor, putative, expressed |
| Pavir.J11045 | 4.05029 | BSD domain-containing protein; BSD, putative, expressed |
| Pavir.Aa00993 | 4.04399 | Dof-type zinc finger DNA-binding family protein; dof zinc finger domain containing protein, putative, expressed |
| Pavir.J15551 | 4.03635 | Myb domain protein 36; MYB family transcription factor, putative, expressed |
| Pavir.J34679 | 4.03472 | NAC domain containing protein 74; no apical meristem protein, putative, expressed |
| Pavir.Aa01234 | 4.01886 | Myb domain protein 19; MYB family transcription factor, putative, expressed |
| Pavir.Bb01182 | 4.01844 | GRAS family transcription factor; SHR, putative, expressed |
| Pavir.Ba01239 | 4.01798 | Myb domain protein 42; MYB family transcription factor, putative, expressed |
| Pavir.Ia00613 | 4.0081 | Indoleacetic acid-induced protein 16; OsIAA13 - Auxin-responsive Aux/IAA gene family member, expressed |
| Pavir.Ca01235 | 3.97701 | MYB family transcription factor, putative, expressed |
| Pavir.J03639 | 3.96255 | NAC domain containing protein 42; no apical meristem protein, putative, expressed |
| Pavir.J06349 | 3.95776 | AP2/B3-like transcriptional factor family protein; B3 DNA binding domain containing protein, expressed |
| Pavir.Cb00045 | 3.95607 | Zinc-finger protein 1; ZOS12-10 - C2H2 zinc finger protein, expressed |
| Pavir.J05241 | 3.95131 | NAC domain containing protein 73; no apical meristem protein, putative, expressed |
| Pavir.Da02025 | 3.92699 | Inorganic H pyrophosphatase family protein; inorganic H+ pyrophosphatase, putative, expressed |
| Pavir.Ca02742 | 3.9211 | CBL-interacting protein kinase 4; CAMK_KIN1/SNF1/Nim1_like.17 - CAMK includes calcium/calmodulin depedent protein kinases, expressed |
| Pavir.Ba03783 | 3.91746 | Nuclear factor Y, subunit A10; nuclear transcription factor Y subunit, putative, expressed |
| Pavir.Bb01218 | 3.91252 | WRKY DNA-binding protein 70; WRKY115, expressed |
| Pavir.Ia04527 | 3.91063 | K-box region and MADS-box transcription factor family protein; OsMADS47 - MADS-box family gene with MIKCc type-box, expressed |
| Pavir.Aa01294 | 3.90916 | Myb domain protein 305; MYB family transcription factor, putative, expressed |
| Pavir.J03407 | 3.8679 | Inorganic H pyrophosphatase family protein; inorganic H+ pyrophosphatase, putative, expressed |
| Pavir.J38128 | 3.86027 | Transcriptional factor B3 family protein / auxin-responsive factor AUX/IAA-related; auxin response factor 14, putative, expressed |
| Pavir.Ha01753 | 3.85567 | bZIP transcription factor family protein; transcription factor, putative, expressed |
| Pavir.J30793 | 3.84169 | Squamosa promoter-binding protein-like (SBP domain) transcription factor family protein; OsSPL18 - SBP-box gene family member, expressed |
| Pavir.Fb00037 | 3.83644 | Ovate family protein 13; ATOFP18/OFP18, putative, expressed |
| Pavir.J07252 | 3.829 | Basic leucine-zipper 52; bZIP transcription factor family protein, putative, expressed |
| Pavir.Ga00965 | 3.8255 | BTB/POZ domain-containing protein; H-BTB3 - Bric-a-Brac, Tramtrack, Broad Complex BTB domain with H family conserved sequence, expressed |
| Pavir.J22986 | 3.80485 | Zinc finger (C2H2 type) family protein; ZOS4-07 - C2H2 zinc finger protein, expressed |
| Pavir.Eb00149 | 3.79536 | Homeodomain-like protein; MYB family transcription factor, putative, expressed |
| Pavir.Da00466 | 3.78231 | Integrase-type DNA-binding superfamily protein; AP2 domain containing protein, expressed |
| Pavir.J39369 | 3.77828 | C2H2 and C2HC zinc fingers superfamily protein; ZOS7-02 - C2H2 zinc finger protein, expressed |
| Pavir.Aa03015 | 3.76484 | bZIP transcription factor family protein; transcription factor, putative, expressed |
| Pavir.Ia01691 | 3.76264 | TRAF-like family protein; MATH domain containing protein, expressed |
| Pavir.J11789 | 3.74738 | Myb domain protein 36; MYB family transcription factor, putative, expressed |
| Pavir.Fa00291 | 3.7394 | BTB-POZ and MATH domain 1; MBTB32 - Bric-a-Brac, Tramtrack, Broad Complex BTB domain with Meprin and TRAF Homology MATH domain, expressed |
| Pavir.Ga00752 | 3.72952 | Myb domain protein 63; MYB family transcription factor, putative, expressed |
| Pavir.Ia04330 | 3.72093 | Basic helix-loop-helix (bHLH) DNA-binding family protein; bHLH family protein, putative, expressed |
| Pavir.Ab02534 | 3.71768 | Plant-specific transcription factor YABBY family protein; YABBY domain containing protein, putative, expressed |
| Pavir.Eb01178 | 3.71458 | Myb domain protein 61; MYB family transcription factor, putative, expressed |
| Pavir.Fb01879 | 3.69519 | C2H2-type zinc finger family protein; ZOS8-10 - C2H2 zinc finger protein, expressed |
| Pavir.Eb00614 | 3.69082 | RING/FYVE/PHD zinc finger superfamily protein; protein binding protein, putative, expressed |
| Pavir.J40848 | 3.67583 | Myb domain protein 61; MYB family transcription factor, putative, expressed |
| Pavir.Ab00366 | 3.67312 | Transcriptional factor B3 family protein / auxin-responsive factor AUX/IAA-related; auxin response factor 5, putative, expressed |
| Pavir.J02717 | 3.6729 | AUX/IAA transcriptional regulator family protein; OsIAA14 - Auxin-responsive Aux/IAA gene family member, expressed |
| Pavir.Da00929 | 3.6494 | BES1/BZR1 homolog 4; BES1/BZR1 homolog protein, putative, expressed |
| Pavir.J39049 | 3.62423 | Arabidopsis NAC domain containing protein 87; no apical meristem protein, putative, expressed |
| Pavir.J16404 | 3.61271 | FAR1-related sequence 5; FAR1 family protein, expressed |
| Pavir.Fb00532 | 3.60923 | NAC domain containing protein 1; no apical meristem protein, putative, expressed |
| Pavir.Ia02208 | 3.60764 | Integrase-type DNA-binding superfamily protein; AP2 domain containing protein, expressed |
| Pavir.Ib00458 | 3.60637 | Basic helix-loop-helix (bHLH) DNA-binding family protein; bHLH family protein, putative, expressed |
| Pavir.Ga01179 | 3.59251 | Myb domain protein 15; MYB family transcription factor, putative, expressed |
| Pavir.Ia00330 | 3.58191 | Homeodomain-like superfamily protein; MYB family transcription factor, putative, expressed |
| Pavir.Ia03777 | 3.57756 | XB3 ortholog 1 in Arabidopsis thaliana; ankyrin repeat family protein, putative, expressed |
| Pavir.Ba00246 | 3.57664 | Arabidopsis NAC domain containing protein 87; no apical meristem protein, putative, expressed |
| Pavir.Cb01264 | 3.56022 | SOS3-interacting protein 1; CAMK_KIN1/SNF1/Nim1_like.20 - CAMK includes calcium/calmodulin depedent protein kinases, expressed |
| Pavir.Eb02390 | 3.55837 | LOB domain-containing protein 41; DUF260 domain containing protein, putative, expressed |
| Pavir.J18000 | 3.53936 | Beta HLH protein 93; BHLH transcription factor, putative, expressed |
| Pavir.J10483 | 3.53916 | C2H2 and C2HC zinc fingers superfamily protein; ZOS1-03 - C2H2 zinc finger protein, expressed |
| Pavir.Ba00224 | 3.53796 | Basic-leucine zipper (bZIP) transcription factor family protein; bZIP transcription factor domain containing protein, expressed |
| Pavir.Ea04181 | 3.51901 | Homeodomain-like superfamily protein; MYB family transcription factor, putative, expressed |
| Pavir.Ca00085 | 3.51472 | WRKY DNA-binding protein 55; WRKY112, expressed |
| Pavir.Ia01043 | 3.51101 | Growth-regulating factor 1; growth regulating factor protein, putative, expressed |
| Pavir.Fa00896 | 3.50084 | Mini zinc finger 2; ZF-HD protein dimerisation region containing protein, expressed |
| Pavir.J03378 | 3.49369 | NAC domain containing protein 17; no apical meristem protein, putative, expressed |
| Pavir.Ia01690 | 3.47689 | TRAF-like family protein; MATH domain containing protein, expressed |
| Pavir.J23197 | 3.47165 | Myb domain protein 9; MYB family transcription factor, putative, expressed |
| Pavir.J39096 | 3.46628 | Mitochondrial transcription termination factor family protein; mTERF family protein, expressed |
| Pavir.Ea03972 | 3.44381 | C2H2-like zinc finger protein; ZOS1-23 - C2H2 zinc finger protein, expressed |
| Pavir.J36282 | 3.4412 | NAC domain containing protein 90; No apical meristem protein, putative, expressed |
| Pavir.Ea00274 | 3.43599 | WRKY DNA-binding protein 51; WRKY10, expressed |
| Pavir.J03334 | 3.42556 | WRKY family transcription factor; WRKY19, expressed |
| Pavir.J40489 | 3.42001 | High mobility group B3; HMG1/2, putative, expressed |
| Pavir.Ba01071 | 3.3926 | GRAS family transcription factor; SHR, putative, expressed |
| Pavir.Ba02533 | 3.39233 | RING/FYVE/PHD zinc finger superfamily protein; expressed protein |
| Pavir.J19599 | 3.38708 | Mini zinc finger 2; ZF-HD protein dimerisation region containing protein, expressed |
| Pavir.Bb00319 | 3.37853 | Nuclear factor Y, subunit A10; nuclear transcription factor Y subunit, putative, expressed |
| Pavir.J36433 | 3.36491 | TEOSINTE BRANCHED 1, cycloidea and PCF transcription factor 2; TCP family transcription factor, putative, expressed |
| Pavir.Aa01011 | 3.36386 | Homeodomain-like superfamily protein; MYB family transcription factor, putative, expressed |
| Pavir.Bb02514 | 3.34744 | Basic leucine-zipper 58; bZIP transcription factor domain containing protein, expressed |
| Pavir.J40260 | 3.34336 | WRKY DNA-binding protein 13; WRKY102, expressed |
| Pavir.J01728 | 3.34303 | Ovate family protein 7; DUF623 domain containing protein, expressed |
| Pavir.J12030 | 3.33472 | WRKY DNA-binding protein 28; WRKY49, expressed |
| Pavir.Ib00526 | 3.33311 | Related to AP2 2; AP2 domain containing protein, expressed |
| Pavir.J05807 | 3.32964 | Transcriptional factor B3 family protein / auxin-responsive factor AUX/IAA-related; auxin response factor 14, putative, expressed |
| Pavir.Bb03699 | 3.29902 | ABA-responsive element binding protein 3; bZIP transcription factor domain containing protein, expressed |
| Pavir.Ea02508 | 3.29695 | BTB-POZ and MATH domain 1; MBTB11 - Bric-a-Brac, Tramtrack, Broad Complex BTB domain with Meprin and TRAF Homology MATH domain, expressed |
| Pavir.Ca00467 | 3.29608 | Phytochrome-associated protein 2; OsIAA17 - Auxin-responsive Aux/IAA gene family member, expressed |
| Pavir.J19159 | 3.29534 | Basic-leucine zipper (bZIP) transcription factor family protein; bZIP family transcription factor, putative, expressed |
| Pavir.J07446 | 3.29463 | Myb domain protein 4; MYB family transcription factor, putative, expressed |
| Pavir.J05543 | 3.29458 | bZIP transcription factor family protein; transcription factor, putative, expressed |
| Pavir.Ha00323 | 3.28397 | Zinc-finger protein 10; ZOS7-01 - C2H2 zinc finger protein, expressed |
| Pavir.J36055 | 3.27455 | MYB family transcription factor, putative, expressed |
| Pavir.J22708 | 3.25986 | WRKY DNA-binding protein 72; WRKY107, expressed |
| Pavir.J05873 | 3.25786 | Beta-6 tubulin; tubulin/FtsZ domain containing protein, putative, expressed |
| Pavir.Ba01556 | 3.24842 | ARF GTPase-activating protein; ARF GTPase-activating domain-containing protein, putative, expressed |
| Pavir.Fb02226 | 3.22836 | PLATZ transcription factor family protein; zinc-binding protein, putative, expressed |
| Pavir.Ib00527 | 3.22328 | Integrase-type DNA-binding superfamily protein; AP2 domain containing protein, expressed |
| Pavir.Ba01626 | 3.21994 | Basic helix-loop-helix (bHLH) DNA-binding superfamily protein; BHLH transcription factor, putative, expressed |
| Pavir.Ba01665 | 3.21446 | NAC domain containing protein 38; no apical meristem protein, putative, expressed |
| Pavir.Bb02654 | 3.20679 | Myb domain protein 42; MYB family transcription factor, putative, expressed |
| Pavir.Bb02594 | 3.20619 | Zinc finger protein 2; ZOS9-18 - C2H2 zinc finger protein, expressed |
| Pavir.J23199 | 3.204 | NAC domain containing protein 80; no apical meristem protein, putative, expressed |
| Pavir.J33301 | 3.20054 | RING/FYVE/PHD zinc finger superfamily protein; zinc finger, C3HC4 type, putative, expressed |
| Pavir.Eb00092 | 3.19127 | Cytokinin response factor 2; AP2 domain containing protein, expressed |
| Pavir.Eb00254 | 3.18795 | Myb domain protein 36; myb-like DNA-binding domain containing protein, putative, expressed |
| Pavir.Ea03729 | 3.18125 | Dof-type zinc finger DNA-binding family protein; dof zinc finger domain containing protein, putative, expressed |
| Pavir.J19191 | 3.15744 | Myb domain protein 86; myb-like DNA-binding domain containing protein, putative, expressed |
| Pavir.Db00693 | 3.15716 | Myb domain protein r1; MYB transcription factor TaMYB1, putative, expressed |
| Pavir.J17623 | 3.15318 | Transcriptional factor B3 family protein / auxin-responsive factor AUX/IAA-related; auxin response factor 14, putative, expressed |
| Pavir.Ba00374 | 3.1479 | SAP domain-containing protein; SAP domain containing protein, expressed |
| Pavir.Eb01618 | 3.13938 | Heat shock transcription factor C1; HSF-type DNA-binding domain containing protein, expressed |
| Pavir.Ea01062 | 3.11477 | Beta-6 tubulin; tubulin/FtsZ domain containing protein, putative, expressed |
| Pavir.Db01743 | 3.11437 | Myb domain protein 20; ODORANT1, putative, expressed |
| Pavir.Ea00964 | 3.11169 | Myb domain protein 108; MYB family transcription factor, putative, expressed |
| Pavir.Ib04170 | 3.10243 | Lateral organ boundaries (LOB) domain family protein; DUF260 domain containing protein, putative, expressed |
| Pavir.J26531 | 3.10206 | NAC domain containing protein 42; no apical meristem protein, putative, expressed |
| Pavir.Ca01558 | 3.09965 | SOS3-interacting protein 1; CAMK_KIN1/SNF1/Nim1_like.20 - CAMK includes calcium/calmodulin depedent protein kinases, expressed |
| Pavir.Ia01111 | 3.09766 | Homeodomain-like superfamily protein; MYB family transcription factor, putative, expressed |
| Pavir.J00079 | 3.08762 | Nuclear factor Y, subunit C11; histone-like transcription factor and archaeal histone, putative, expressed |
| Pavir.Ea02962 | 3.05786 | Heat shock transcription factor C1; HSF-type DNA-binding domain containing protein, expressed |
| Pavir.Ea03193 | 3.05069 | bZIP transcription factor family protein; transcription factor, putative, expressed |
| Pavir.Bb03208 | 3.05055 | Brassinosteroid signalling positive regulator (BZR1) family protein; BES1/BZR1 homolog protein, putative, expressed |
| Pavir.Ia02979 | 3.04355 | GRAS family transcription factor; SHORT-ROOT, putative, expressed |
| Pavir.Ba00442 | 3.03844 | Nuclear factor Y, subunit A3; nuclear transcription factor Y subunit, putative, expressed |
| Pavir.Fa00850 | 3.03777 | Myb domain protein 20; MYB family transcription factor, putative, expressed |
| Pavir.Hb01807 | 3.03182 | bZIP transcription factor family protein; transcription factor, putative, expressed |
| Pavir.J32718 | 3.02252 | Transcriptional factor B3 family protein / auxin-responsive factor AUX/IAA-related; auxin response factor 5, putative, expressed |
| Pavir.J27104 | 3.02191 | Scarecrow-like 3; GRAS family transcription factor containing protein, expressed |
| Pavir.Eb02324 | 3.01503 | Integrase-type DNA-binding superfamily protein; AP2 domain containing protein, expressed |
| Pavir.Gb02382 | 3.01253 | WRKY family transcription factor; WRKY46, expressed |
| Pavir.Ea00414 | 3.00219 | Integrase-type DNA-binding superfamily protein; AP2 domain containing protein, expressed |
| Pavir.Ha00781 | -3.00481 | NAC domain containing protein 47; No apical meristem protein, putative, expressed |
| Pavir.Aa01709 | -3.0109 | ZOS2-09 - C2H2 zinc finger protein, expressed |
| Pavir.J24672 | -3.01425 | Basic-leucine zipper (bZIP) transcription factor family protein; bZIP transcription factor domain containing protein, expressed |
| Pavir.J12413 | -3.03364 | Plant regulator RWP-RK family protein; NIN, putative, expressed |
| Pavir.Hb00732 | -3.0367 | Myb domain protein 60; MYB family transcription factor, putative, expressed |
| Pavir.Db01602 | -3.05094 | Multiprotein bridging factor 1C; endothelial differentiation-related factor 1, putative, expressed |
| Pavir.J25613 | -3.0965 | Disease resistance protein (CC-NBS-LRR class) family; stripe rust resistance protein Yr10, putative, expressed |
| Pavir.Ia04505 | -3.09752 | CBL-interacting protein kinase 9; CAMK_KIN1/SNF1/Nim1_like.15 - CAMK includes calcium/calmodulin depedent protein kinases, expressed |
| Pavir.Ea04142 | -3.10483 | Telomeric repeat binding protein 1; MYB family transcription factor, putative, expressed |
| Pavir.Ia03213 | -3.12324 | Nuclear factor Y, subunit B3; histone-like transcription factor and archaeal histone, putative, expressed |
| Pavir.Aa00685 | -3.13535 | Rubisco methyltransferase family protein; SET domain-containing protein, putative, expressed |
| Pavir.Ea03575 | -3.16862 | BTB and TAZ domain protein 3; BTBZ1 - Bric-a-Brac, Tramtrack, and Broad Complex BTB domain with TAZ zinc finger and Calmodulin-binding domains, expressed |
| Pavir.Da00760 | -3.17702 | GATA type zinc finger transcription factor family protein; GATA zinc finger domain containing protein, expressed |
| Pavir.Ib01803 | -3.18115 | Znc finger protein 2; ZOS9-11 - C2H2 zinc finger protein, expressed |
| Pavir.Da01536 | -3.24196 | Far-red impaired responsive (FAR1) family protein; expressed protein |
| Pavir.Da02163 | -3.24857 | WRKY family transcription factor; WRKY113, expressed |
| Pavir.Bb03389 | -3.25519 | SAP domain-containing protein; SAP domain containing protein, expressed |
| Pavir.Db01897 | -3.26177 | RAD-like 1; MYB family transcription factor, putative, expressed |
| Pavir.Eb02679 | -3.26789 | RAD-like 1; MYB family transcription factor, putative, expressed |
| Pavir.Ia01199 | -3.27095 | AP2/B3-like transcriptional factor family protein; B3 DNA binding domain containing protein |
| Pavir.J18467 | -3.27424 | VERDANDI; B3 DNA binding domain containing protein, expressed |
| Pavir.Ib00525 | -3.28342 | Related to AP2 12; AP2 domain containing protein, expressed |
| Pavir.Ab00856 | -3.30451 | Basic-leucine zipper (bZIP) transcription factor family protein; bZIP transcription factor domain containing protein, expressed |
| Pavir.J07102 | -3.30585 | Mitochondrial transcription termination factor family protein; mTERF family protein, expressed |
| Pavir.Ia03786 | -3.32459 | Myb domain protein 111; MYB family transcription factor, putative, expressed |
| Pavir.Ca01033 | -3.32995 | High mobility group; SSRP1-like FACT complex subunit, putative, expressed |
| Pavir.Ea01032 | -3.36232 | CBL-interacting protein kinase 1; CAMK_KIN1/SNF1/Nim1_like.9 - CAMK includes calcium/calmodulin depedent protein kinases, expressed |
| Pavir.Ia03096 | -3.38028 | Zinc finger protein 2; ZOS9-11 - C2H2 zinc finger protein, expressed |
| Pavir.Ia03258 | -3.38461 | C2H2 and C2HC zinc fingers superfamily protein; ZOS9-11 - C2H2 zinc finger protein, expressed |
| Pavir.J20023 | -3.40894 | SMAD/FHA domain-containing protein; FHA domain containing protein, putative, expressed |
| Pavir.J01689 | -3.47848 | BTB and TAZ domain protein 3; BTBZ1 - Bric-a-Brac, Tramtrack, and Broad Complex BTB domain with TAZ zinc finger and Calmodulin-binding domains, expressed |
| Pavir.J02276 | -3.49729 | NAC domain containing protein 90; no apical meristem protein, putative, expressed |
| Pavir.Ib02920 | -3.54279 | Related to AP2 4; ethylene-responsive transcription factor, putative, expressed |
| Pavir.Hb00869 | -3.54665 | NAC domain containing protein 47; No apical meristem protein, putative, expressed |
| Pavir.J38980 | -3.59623 | Protein of unknown function (DUF607); E2F-related protein, putative, expressed |
| Pavir.J34811 | -3.60632 | Transcription factor jumonji (jmjC) domain-containing protein; jmjC domain containing protein, expressed |
| Pavir.J17924 | -3.61089 | Mitochondrial transcription termination factor family protein; mTERF domain containing protein, expressed |
| Pavir.J15257 | -3.65662 | SNF7 family protein; SNF7 domain containing protein, putative, expressed |
| Pavir.Ib01924 | -3.6602 | Nuclear factor Y, subunit B3; histone-like transcription factor and archaeal histone, putative, expressed |
| Pavir.Bb02238 | -3.66866 | Zinc finger protein 2; ZOS9-10 - C2H2 zinc finger protein, expressed |
| Pavir.Ia02495 | -3.6841 | Related to AP2 4; ethylene-responsive transcription factor, putative, expressed |
| Pavir.J21984 | -3.75041 | Basic-leucine zipper (bZIP) transcription factor family protein; E2F-related protein, putative, expressed |
| Pavir.Db00028 | -3.78321 | NAC domain containing protein 36; no apical meristem protein, putative, expressed |
| Pavir.J10894 | -3.79526 | Myb domain protein 60; MYB family transcription factor, putative, expressed |
| Pavir.J16587 | -3.81598 | LOB domain-containing protein 37; DUF260 domain containing protein, putative, expressed |
| Pavir.Ga00104 | -3.83422 | SNF2 domain-containing protein / helicase domain-containing protein / zinc finger protein-related; expressed protein |
| Pavir.Ib03017 | -3.84724 | NAC domain containing protein 32; no apical meristem protein, putative, expressed |
| Pavir.J16741 | -3.86193 | Homeobox protein 31; ZF-HD protein dimerisation region containing protein, expressed |
| Pavir.J19009 | -3.8985 | AP2/B3-like transcriptional factor family protein; B3 DNA binding domain containing protein, expressed |
| Pavir.J29604 | -3.9506 | Rubisco methyltransferase family protein; SET domain-containing protein, putative, expressed |
| Pavir.Cb02041 | -4.0719 | RING/FYVE/PHD zinc finger superfamily protein; expressed protein |
| Pavir.J24408 | -4.07716 | FHA domain containing protein, putative, expressed |
| Pavir.Cb00750 | -4.0998 | Disease resistance protein (CC-NBS-LRR class) family; expressed protein |
| Pavir.J18932 | -4.10103 | Acyl-CoA N-acyltransferases (NAT) superfamily protein; acetyltransferase, GNAT family, putative, expressed |
| Pavir.J09678 | -4.13556 | FAR1-related sequence 4; FAR1 family protein, expressed |
| Pavir.Hb01808 | -4.20254 | NAC domain containing protein 61; no apical meristem protein, putative, expressed |
| Pavir.Eb00995 | -4.22795 | BED zinc finger ;hAT family dimerisation domain; ZOS9-05 - C2H2 zinc finger protein, expressed |
| Pavir.J00335 | -4.23895 | FRS (FAR1 Related Sequences) transcription factor family; SWIM zinc finger family protein, putative, expressed |
| Pavir.Ha01884 | -4.27393 | CBL-interacting protein kinase 3; CAMK_KIN1/SNF1/Nim1_like.37 - CAMK includes calcium/calmodulin depedent protein kinases, expressed |
| Pavir.Da00549 | -4.34085 | Basic-leucine zipper (bZIP) transcription factor family protein; bZIP transcription factor domain containing protein, expressed |
| Pavir.Ga02445 | -4.5112 | AP2/B3-like transcriptional factor family protein; B3 DNA binding domain containing protein, expressed |
| Pavir.Ga01717 | -4.52124 | GRAS family transcription factor; SCARECROW, putative, expressed |
| Pavir.Ca00340 | -4.55495 | Homeobox protein 21; ZF-HD protein dimerisation region containing protein, expressed |
| Pavir.J37837 | -4.57371 | WRKY DNA-binding protein 57; WRKY3, expressed |
| Pavir.Fa01616 | -4.58788 | Disease resistance protein (CC-NBS-LRR class) family; NBS-LRR type disease resistance protein, putative, expressed |
| Pavir.J36164 | -4.62238 | Zinc finger protein 2; ZOS9-12 - C2H2 zinc finger protein, expressed |
| Pavir.Ia01299 | -4.64532 | LOB domain-containing protein 37; DUF260 domain containing protein, putative, expressed |
| Pavir.Db01250 | -4.65699 | GATA type zinc finger transcription factor family protein; GATA zinc finger domain containing protein, expressed |
| Pavir.Bb02239 | -4.92765 | Zinc finger protein 2; ZOS9-12 - C2H2 zinc finger protein, expressed |
| Pavir.Ea02402 | -5.01494 | SMAD/FHA domain-containing protein; FHA domain containing protein, putative, expressed |
| Pavir.Ba02999 | -5.47064 | Integrase-type DNA-binding superfamily protein; AP2 domain containing protein, expressed |
| Pavir.Hb01240 | -5.71164 | Putative endonuclease or glycosyl hydrolase with C2H2-type zinc finger domain; ZOS9-06 - C2H2 zinc finger protein, expressed |
| Pavir.Db01231 | -5.93954 | Basic-leucine zipper (bZIP) transcription factor family protein; bZIP transcription factor domain containing protein, expressed |
| Pavir.Ib01420 | -6.95906 | Myb domain protein 12; MYB family transcription factor, putative, expressed |
| Pavir.J24912 | -7.05979 | LIM domain-containing protein; LIM domain-containing protein, putative, expressed |

**Supplementary Table 5: List of drought-responsive genes identified in VS16 compared to AP13**

| Gene ID | Log2FC | Description |
| --- | --- | --- |
| Pavir.Ea00003 | 7.97093 | Tonoplast intrinsic protein 4;1; aquaporin protein, putative, expressed |
| Pavir.Cb01832 | 6.85293 | Tonoplast intrinsic protein 4;1; aquaporin protein, putative, expressed |
| Pavir.Ca00461 | 6.57124 | Tonoplast intrinsic protein 4;1; aquaporin protein, putative, expressed |
| Pavir.Aa02820 | 5.95073 | NOD26-like intrinsic protein 4;2; aquaporin protein, putative, expressed |
| Pavir.Ab01231 | 5.57162 | NOD26-like intrinsic protein 1;2; aquaporin protein, putative, expressed |
| Pavir.J09715 | 5.01203 | NOD26-like intrinsic protein 1;2; aquaporin protein, putative, expressed |
| Pavir.Ia02110 | 4.89046 | NOD26-like intrinsic protein 5;1; aquaporin protein, putative, expressed |
| Pavir.Aa00868 | 3.44236 | Plasma membrane intrinsic protein 1;5; aquaporin protein, putative, expressed |
| Pavir.J37677 | 3.09067 | Plasma membrane intrinsic protein 1;5; aquaporin protein, putative, expressed |
| Pavir.Ia01421 | 3.08766 | NOD26-like intrinsic protein 5;1; aquaporin protein, putative, expressed |
| Pavir.Db01217 | 3.067 | delta tonoplast integral protein; aquaporin protein, putative, expressed |
| Pavir.Ba02483 | 3.02598 | Plasma membrane intrinsic protein 2; aquaporin protein, putative, expressed |
| Pavir.Bb01841 | -5.23059 | Plasma membrane intrinsic protein 2; aquaporin protein, putative, expressed |
| Pavir.Ba01199 | -5.25504 | Plasma membrane intrinsic protein 3; aquaporin protein, putative, expressed |

**Supplementary Table 6: List of heat-responsive genes identified in VS16 compared to AP13**

| Gene ID | Log2FC | Description |
| --- | --- | --- |
| Pavir.Ib01427 | 5.5287 | Heat shock protein DnaJ with tetratricopeptide repeat; DNAJ heat shock N-terminal domain-containing protein, putative, expressed |
| Pavir.J24721 | 5.48338 | Heat shock cognate protein 70-1; DnaK family protein, putative, expressed |
| Pavir.J41002 | 4.43179 | DNAJ heat shock family protein; dnaJ domain containing protein, expressed |
| Pavir.J40688 | 4.16865 | DNAJ heat shock N-terminal domain-containing protein; dehydrin family protein, expressed |
| Pavir.Bb00967 | 4.03172 | DNAJ heat shock N-terminal domain-containing protein; dnaJ domain containing protein, expressed |
| Pavir.Ia03540 | 3.92154 | Tetratricopeptide repeat (TPR)-like superfamily protein; DNAJ heat shock N-terminal domain-containing protein, putative, expressed |
| Pavir.Ab01923 | 3.62978 | DNAJ heat shock N-terminal domain-containing protein; chaperone protein dnaJ 10, putative, expressed |
| Pavir.J23559 | 3.13939 | Heat shock protein DnaJ with tetratricopeptide repeat; DNAJ heat shock N-terminal domain-containing protein, putative, expressed |
| Pavir.Ab02498 | -3.00292 | Chaperone DnaJ-domain superfamily protein; heat shock protein DnaJ, putative, expressed |
| Pavir.Db00557 | -3.02951 | Chaperone DnaJ-domain superfamily protein; heat shock protein DnaJ, putative, expressed |
| Pavir.J02667 | -3.03513 | Chaperone DnaJ-domain superfamily protein; heat shock protein DnaJ, putative, expressed |
| Pavir.Aa02120 | -3.04971 | Chaperone protein dnaJ-related; drought-induced protein 1, putative, expressed |
| Pavir.J11181 | -3.16628 | DnaJ/Hsp40 cysteine-rich domain superfamily protein; tsi1-interacting protein TSIP1, putative, expressed |
| Pavir.J34808 | -3.18139 | Heat shock protein 70; DnaK family protein, putative, expressed |
| Pavir.J01685 | -3.30285 | Chloroplast heat shock protein 70-1; DnaK family protein, putative, expressed |
| Pavir.J11930 | -3.36232 | Chaperone protein dnaJ-related; drought-induced protein 1, putative, expressed |
| Pavir.J08596 | -3.51 | Chaperone DnaJ-domain superfamily protein; heat shock protein DnaJ, putative, expressed |
| Pavir.Fa00703 | -3.51631 | DnaJ/Hsp40 cysteine-rich domain superfamily protein; expressed protein |
| Pavir.Eb03020 | -3.56244 | DNAJ heat shock N-terminal domain-containing protein; heat shock protein DnaJ, putative, expressed |
| Pavir.Ha01274 | -3.60451 | Chaperone DnaJ-domain superfamily protein; heat shock protein DnaJ, putative, expressed |
| Pavir.Ea02970 | -3.64637 | DNAJ heat shock N-terminal domain-containing protein; heat shock protein DnaJ, putative, expressed |
| Pavir.J12343 | -3.65432 | Heat shock protein 70; DnaK family protein, putative, expressed |
| Pavir.Cb01655 | -3.8408 | Chaperone DnaJ-domain superfamily protein; heat shock protein DnaJ, putative, expressed |
| Pavir.J35718 | -3.84138 | DNAJ heat shock N-terminal domain-containing protein; heat shock protein DnaJ, putative, expressed |
| Pavir.J16614 | -3.85727 | Chaperone DnaJ-domain superfamily protein; heat shock protein DnaJ, putative, expressed |
| Pavir.J13048 | -3.87888 | Chaperone DnaJ-domain superfamily protein; heat shock protein DnaJ, putative, expressed |
| Pavir.Cb00021 | -3.97194 | Chaperone DnaJ-domain superfamily protein; heat shock protein DnaJ, putative, expressed |
| Pavir.Cb00837 | -4.09747 | Chaperone DnaJ-domain superfamily protein; heat shock protein DnaJ, putative, expressed |
| Pavir.Ca02433 | -4.1177 | Chaperone DnaJ-domain superfamily protein; heat shock protein DnaJ, putative, expressed |

**Supplementary Table 7: List of flooding-responsive genes identified in VS16 compared to AP13**

| Gene ID | Log2FC | Description |
| --- | --- | --- |
| Pavir.Da00154 | 8.93114 | Xyloglucan endotransglucosylase/hydrolase 25; glycosyl hydrolases family 16, putative, expressed |
| Pavir.Ga00388 | 7.66341 | Xyloglucan endotransglucosylase/hydrolase 9; glycosyl hydrolases family 16 protein, protein, expressed |
| Pavir.Ab02402 | 7.53719 | Expansin B3; expansin precursor, putative, expressed |
| Pavir.Ca01085 | 7.03993 | Leucine-rich repeat transmembrane protein kinase; leucine-rich repeat receptor protein kinase EXS precursor, putative, expressed |
| Pavir.J05963 | 7.0287 | Leucine-rich repeat transmembrane protein kinase; leucine-rich repeat receptor protein kinase EXS precursor, putative, expressed |
| Pavir.J37973 | 6.79487 | GRF zinc finger family protein |
| Pavir.Ea03672 | 6.52076 | Adenine nucleotide alpha hydrolases-like superfamily protein; universal stress protein domain containing protein, putative, expressed |
| Pavir.Gb00304 | 6.37009 | Xyloglucan endotransglucosylase/hydrolase 9; glycosyl hydrolases family 16 protein, protein, expressed |
| Pavir.Ba00925 | 6.33641 | Leucine-rich repeat transmembrane protein kinase; leucine-rich repeat receptor protein kinase EXS precursor, putative, expressed |
| Pavir.Bb02784 | 6.22624 | Leucine-rich repeat transmembrane protein kinase; leucine-rich repeat receptor protein kinase EXS precursor, putative, expressed |
| Pavir.Cb00432 | 5.92522 | Cyclin D2;1; cyclin, putative, expressed |
| Pavir.Gb01070 | 5.62195 | CYCLIN B2;4; cyclin, putative, expressed |
| Pavir.J03847 | 5.58929 | Cyclin D2;1; cyclin, putative, expressed |
| Pavir.J17645 | 5.58796 | Leucine-rich repeat transmembrane protein kinase; SHR5-receptor-like kinase, putative, expressed |
| Pavir.Ha01388 | 5.57551 | Xyloglucan endotransglucosylase/hydrolase 5; glycosyl hydrolases family 16, putative, expressed |
| Pavir.Da00153 | 5.4003 | Xyloglucan endotransglucosylase/hydrolase family protein; glycosyl hydrolases family 16, putative, expressed |
| Pavir.J13125 | 5.36692 | Xyloglucan endotransglucosylase/hydrolase family protein; glycosyl hydrolases family 16, putative, expressed |
| Pavir.J06478 | 5.36402 | SSXT family protein; GRF-interacting factor 1, putative, expressed |
| Pavir.Ba02217 | 5.35578 | CYCLIN D1;1; cyclin, putative, expressed |
| Pavir.J11763 | 5.2749 | Growth-regulating factor 1; growth-regulating factor, putative, expressed |
| Pavir.Fa01434 | 5.27141 | Xyloglucan endotransglucosylase/hydrolase 25; glycosyl hydrolases family 16, putative, expressed |
| Pavir.Ib03928 | 5.26526 | Cyclin A3;1; cyclin, putative, expressed |
| Pavir.J18599 | 5.25576 | Xyloglucan endotransglucosylase/hydrolase 12; glycosyl hydrolases family 16, putative, expressed |
| Pavir.Ha00073 | 5.23947 | Cyclin-dependent kinase inhibitor family protein; cyclin-dependent kinase inhibitor, putative, expressed |
| Pavir.Aa00711 | 5.21448 | Expansin A1; expansin precursor, putative, expressed |
| Pavir.Ia00282 | 5.16465 | Expansin A4; expansin precursor, putative, expressed |
| Pavir.Gb01066 | 5.13734 | CYCLIN B2;4; cyclin, putative, expressed |
| Pavir.Fb01781 | 5.01742 | Cyclin D2;1; cyclin, putative, expressed |
| Pavir.Hb01326 | 4.94104 | Leucine-rich repeat protein kinase family protein; receptor protein kinase TMK1 precursor, putative, expressed |
| Pavir.Bb01836 | 4.91271 | Expansin B4; expansin precursor, putative, expressed |
| Pavir.J01055 | 4.90119 | Expansin B2; expansin precursor, putative, expressed |
| Pavir.Ia02187 | 4.77781 | Expansin B2; expansin precursor, putative, expressed |
| Pavir.J10079 | 4.76816 | Xyloglucan endotransglucosylase/hydrolase 25; glycosyl hydrolases family 16, putative, expressed |
| Pavir.Gb00512 | 4.74909 | Leucine-rich repeat transmembrane protein kinase; SHR5-receptor-like kinase, putative, expressed |
| Pavir.Ga00724 | 4.68585 | Growth-regulating factor 5; growth-regulating factor, putative, expressed |
| Pavir.Db01201 | 4.64786 | Replicon protein A2; RPA2A - Putative single-stranded DNA binding complex subunit 2, expressed |
| Pavir.J40222 | 4.61562 | Transmembrane kinase 1; receptor protein kinase TMK1 precursor, putative, expressed |
| Pavir.J21922 | 4.60582 | Replication factor-A protein 1-related; RPA1C - Putative single-stranded DNA binding complex subunit 1, expressed |
| Pavir.Db00109 | 4.54726 | Xyloglucan endotransglucosylase/hydrolase 25; glycosyl hydrolases family 16, putative, expressed |
| Pavir.Gb00591 | 4.52919 | Growth-regulating factor, putative, expressed |
| Pavir.Ib02440 | 4.46418 | Expansin B2; expansin precursor, putative, expressed |
| Pavir.Ba02013 | 4.3892 | Cyclin-dependent kinase inhibitor family protein; cyclin-dependent kinase inhibitor, putative, expressed |
| Pavir.J16398 | 4.31757 | Alpha-amylase-like; alpha-amylase precursor, putative, expressed |
| Pavir.Fb02013 | 4.28308 | Cyclin-dependent kinase B2;1; cyclin-dependent kinase B2-1, putative, expressed |
| Pavir.J31534 | 4.26296 | Major facilitator superfamily protein; tetracycline transporter protein, putative, expressed |
| Pavir.Ca02411 | 4.23673 | Cyclin A2;4; cyclin, putative, expressed |
| Pavir.Ib03630 | 4.19594 | Expansin B2; expansin precursor, putative, expressed |
| Pavir.J22550 | 4.16573 | Transmembrane kinase 1; receptor protein kinase TMK1 precursor, putative, expressed |
| Pavir.Aa00782 | 4.10316 | Transducin family protein / WD-40 repeat family protein; WD repeat-containing protein, putative, expressed |
| Pavir.J02642 | 4.09654 | Leucine-rich repeat transmembrane protein kinase; leucine-rich repeat receptor protein kinase EXS precursor, putative, expressed |
| Pavir.J05998 | 4.04025 | Expansin A6; expansin precursor, putative, expressed |
| Pavir.Ib02225 | 4.02962 | Expansin B2; expansin precursor, putative, expressed |
| Pavir.J31818 | 4.02865 | Adenine nucleotide alpha hydrolases-like superfamily protein; universal stress protein domain containing protein, putative, expressed |
| Pavir.Fb01262 | 4.00076 | Adenine nucleotide alpha hydrolases-like superfamily protein; universal stress protein domain containing protein, putative, expressed |
| Pavir.Aa03451 | 3.99195 | Cyclin p4;1; cyclin, putative, expressed |
| Pavir.Ab02792 | 3.95279 | Transducin family protein / WD-40 repeat family protein; WD repeat-containing protein, putative, expressed |
| Pavir.Ia01549 | 3.94025 | Expansin B2; expansin precursor, putative, expressed |
| Pavir.J02319 | 3.93698 | Cyclin A2;4; cyclin, putative, expressed |
| Pavir.J04626 | 3.92257 | Ethylene-forming enzyme; 1-aminocyclopropane-1-carboxylate oxidase protein, putative, expressed |
| Pavir.Fa01284 | 3.89568 | Xyloglucan endotransglucosylase/hydrolase family protein; glycosyl hydrolases family 16, putative, expressed |
| Pavir.Ib02219 | 3.8604 | Expansin B2; expansin precursor, putative, expressed |
| Pavir.J25986 | 3.77908 | Ethylene-forming enzyme; 1-aminocyclopropane-1-carboxylate oxidase protein, putative, expressed |
| Pavir.Ea03943 | 3.73834 | Leucine-rich repeat transmembrane protein kinase; leucine-rich repeat receptor protein kinase EXS precursor, putative, expressed |
| Pavir.Fb01452 | 3.73589 | Xyloglucan endotransglucosylase/hydrolase family protein; glycosyl hydrolases family 16, putative, expressed |
| Pavir.J39558 | 3.71957 | Xyloglucan endotransglucosylase/hydrolase 25; glycosyl hydrolases family 16, putative, expressed |
| Pavir.Ia04504 | 3.7193 | Leucine-rich repeat protein kinase family protein; leucine-rich repeat transmembrane protein kinase, putative, expressed |
| Pavir.J03977 | 3.69671 | Cell division control, Cdc6; CDC6 - Putative DNA replication initiation protein, expressed |
| Pavir.J37501 | 3.69474 | Subtilisin-like serine endopeptidase family protein; OsSub17 - Putative Subtilisin homologue, expressed |
| Pavir.J02482 | 3.65812 | Adenine nucleotide alpha hydrolases-like superfamily protein; universal stress protein domain containing protein, putative, expressed |
| Pavir.Ia04634 | 3.57585 | Cyclin d5;1; cyclin, putative, expressed |
| Pavir.Ba01469 | 3.56885 | Expansin-like A2; expansin precursor, putative, expressed |
| Pavir.Fa00056 | 3.5152 | Transmembrane kinase-like 1; protein Kinase-like protein TMKL1 precursor, putative, expressed |
| Pavir.Ia01043 | 3.51101 | Growth-regulating factor 1; growth regulating factor protein, putative, expressed |
| Pavir.Aa00841 | 3.38735 | Expansin B4; expansin precursor, putative, expressed |
| Pavir.J33353 | 3.38662 | Subtilase family protein; OsSub14 - Putative Subtilisin homologue, expressed |
| Pavir.J05088 | 3.36755 | Leucine-rich repeat transmembrane protein kinase protein; receptor-like protein kinase, putative, expressed |
| Pavir.Ea00772 | 3.35809 | Adenine nucleotide alpha hydrolases-like superfamily protein; universal stress protein domain containing protein, putative, expressed |
| Pavir.Cb02062 | 3.3427 | Replicon protein A2; RPA2A - Putative single-stranded DNA binding complex subunit 2, expressed |
| Pavir.Ia02829 | 3.3344 | Expansin B2; expansin precursor, putative, expressed |
| Pavir.Ib02223 | 3.33149 | Expansin B2; expansin precursor, putative, expressed |
| Pavir.Ca02530 | 3.28907 | Cyclin d5;1; cyclin, putative, expressed |
| Pavir.Ba02414 | 3.24228 | Expansin B4; expansin precursor, putative, expressed |
| Pavir.Ia02182 | 3.22967 | Expansin B2; expansin precursor, putative, expressed |
| Pavir.Ab01313 | 3.22506 | Aacetyl-CoA synthetase; AMP-binding enzyme, putative, expressed |
| Pavir.Ba01006 | 3.17668 | Xyloglucan endotransglucosylase/hydrolase 32; glycosyl hydrolases family 16, putative, expressed |
| Pavir.J21920 | 3.17366 | Cyclin D2;1; cyclin, putative, expressed |
| Pavir.Aa01116 | 3.16398 | Replication protein A, subunit RPA32; RPA2B - Putative single-stranded DNA binding complex subunit 2, expressed |
| Pavir.J14443 | 3.1214 | Adenine nucleotide alpha hydrolases-like superfamily protein; universal stress protein domain containing protein, putative, expressed |
| Pavir.Cb00061 | 3.05414 | Alpha-amylase-like; alpha-amylase precursor, putative, expressed |
| Pavir.Ib01582 | 3.03436 | Uncharacterized protein; cyclin-related protein, putative, expressed |
| Pavir.Ab01626 | 3.01465 | Cyclin family; microtubule-binding protein TANGLED1, putative, expressed |
| Pavir.Ea01068 | 3.0022 | Cyclin family protein; cyclin, putative, expressed |
| Pavir.Ib04474 | -3.02657 | Xyloglucan endotransglucosylase/hydrolase 30; glycosyl hydrolases family 16, putative, expressed |
| Pavir.J01888 | -3.17492 | Adenine nucleotide alpha hydrolases-like superfamily protein; universal stress protein domain containing protein, putative, expressed |
| Pavir.J06926 | -3.20006 | Replication factor-A protein 1-related; RPA1C - Putative single-stranded DNA binding complex subunit 1, expressed |
| Pavir.Ib02411 | -3.25954 | Rhodanese/Cell cycle control phosphatase superfamily protein; expressed protein |
| Pavir.Ia02145 | -3.2887 | Chloroplast beta-amylase; beta-amylase, putative, expressed |
| Pavir.Ea00401 | -3.34131 | Adenine nucleotide alpha hydrolases-like superfamily protein; universal stress protein domain containing protein, putative, expressed |
| Pavir.J04705 | -3.3877 | Leucine-rich repeat transmembrane protein kinase; SHR5-receptor-like kinase, putative, expressed |
| Pavir.Ca02828 | -3.38891 | Nine-cis-epoxycarotenoid dioxygenase 4; carotenoid cleavage dioxygenase, putative, expressed |
| Pavir.Cb00047 | -3.40544 | Nine-cis-epoxycarotenoid dioxygenase 3; 9-cis-epoxycarotenoid dioxygenase 1, chloroplast precursor, putative, expressed |
| Pavir.J38555 | -3.58303 | RPA70-kDa subunit B; expressed protein |
| Pavir.Ga02401 | -3.71698 | O-acetylserine (thiol) lyase B; cysteine synthase, chloroplast/chromoplast precursor, putative, expressed |
| Pavir.Ab02771 | -3.7392 | Nine-cis-epoxycarotenoid dioxygenase 4; 9-cis-epoxycarotenoid dioxygenase 1, chloroplast precursor, putative, expressed |
| Pavir.Aa01044 | -3.7416 | Nine-cis-epoxycarotenoid dioxygenase 4; 9-cis-epoxycarotenoid dioxygenase 1, chloroplast precursor, putative, expressed |
| Pavir.J30088 | -3.75305 | Carotenoid cleavage dioxygenase 1; carotenoid cleavage dioxygenase, putative, expressed |
| Pavir.Ba03436 | -3.85182 | Leucine-rich repeat transmembrane protein kinase; SHR5-receptor-like kinase, putative, expressed |
| Pavir.Eb00451 | -4.11109 | Adenine nucleotide alpha hydrolases-like superfamily protein; universal stress protein domain containing protein, putative, expressed |
| Pavir.J36211 | -4.13895 | Beta-amylase 5; beta-amylase, putative, expressed |
| Pavir.J32180 | -4.96437 | Universal stress protein domain containing protein, putative, expressed |
| Pavir.J08037 | -6.74544 | RPA70-kDa subunit B; expressed protein |
| Pavir.J10011 | -7.2897 | Leucine-rich repeat transmembrane protein kinase; SHR5-receptor-like kinase, putative, expressed |

**Supplementary Table 8: List of salinity-responsive genes identified in VS16 compared to AP13**

| Gene ID | Log2FC | Description |
| --- | --- | --- |
| Pavir.Ea00003 | 7.97093 | Tonoplast intrinsic protein 4;1; aquaporin protein, putative, expressed |
| Pavir.Cb01832 | 6.85293 | Tonoplast intrinsic protein 4;1; aquaporin protein, putative, expressed |
| Pavir.Ca00461 | 6.57124 | Tonoplast intrinsic protein 4;1; aquaporin protein, putative, expressed |
| Pavir.Aa02820 | 5.95073 | NOD26-like intrinsic protein 4;2; aquaporin protein, putative, expressed |
| Pavir.Ab01231 | 5.57162 | NOD26-like intrinsic protein 1;2; aquaporin protein, putative, expressed |
| Pavir.J09715 | 5.01203 | NOD26-like intrinsic protein 1;2; aquaporin protein, putative, expressed |
| Pavir.Ab02135 | 4.98671 | Phenylalanine ammonia-lyase 4; phenylalanine ammonia-lyase, putative, expressed |
| Pavir.Ab02345 | 4.98358 | PHE ammonia lyase 1; phenylalanine ammonia-lyase, putative, expressed |
| Pavir.Ia02110 | 4.89046 | NOD26-like intrinsic protein 5;1; aquaporin protein, putative, expressed |
| Pavir.Aa01274 | 4.82093 | PHE ammonia lyase 1; phenylalanine ammonia-lyase, putative, expressed |
| Pavir.J08362 | 4.77952 | ABC transporter family protein; ABC transporter, ATP-binding protein, putative, expressed |
| Pavir.J10330 | 4.52716 | Aldehyde dehydrogenase 3F1; aldehyde dehydrogenase, putative, expressed |
| Pavir.J35121 | 4.46732 | PHE ammonia lyase 1; phenylalanine ammonia-lyase, putative, expressed |
| Pavir.Ga00677 | 4.46327 | Aldehyde dehydrogenase 3F1; aldehyde dehydrogenase, putative, expressed |
| Pavir.Bb03622 | 4.36119 | Aldehyde dehydrogenase 22A1; aldehyde dehydrogenase, putative, expressed |
| Pavir.Db00464 | 4.32852 | Leucine-rich repeat protein kinase family protein; BRASSINOSTEROID INSENSITIVE 1 precursor, putative, expressed |
| Pavir.J36439 | 4.32258 | ABC2 homolog 6; ABC transporter, ATP-binding protein, putative, expressed |
| Pavir.Eb02933 | 4.28445 | ABC transporter family protein; ABC transporter, ATP-binding protein, putative, expressed |
| Pavir.Ba00556 | 4.17029 | Brassinosteroid signalling positive regulator (BZR1) family protein; BES1/BZR1 homolog protein, putative, expressed |
| Pavir.J40688 | 4.16865 | DNAJ heat shock N-terminal domain-containing protein; dehydrin family protein, expressed |
| Pavir.J25520 | 4.1585 | Phenylalanine ammonia-lyase 4; phenylalanine ammonia-lyase, putative, expressed |
| Pavir.Fa00955 | 3.95946 | Leucine-rich repeat protein kinase family protein; BRASSINOSTEROID INSENSITIVE 1 precursor, putative, expressed |
| Pavir.Ea01974 | 3.76667 | ATP binding cassette subfamily B4; MDR-like ABC transporter, putative, expressed |
| Pavir.Bb00325 | 3.74572 | Multidrug resistance-associated protein 3; ABC transporter family protein, putative, expressed |
| Pavir.Ab02939 | 3.65043 | NSP-interacting kinase 1; BRASSINOSTEROID INSENSITIVE 1-associated receptor kinase 1 precursor, putative, expressed |
| Pavir.Eb02645 | 3.56862 | ABC-2 and Plant PDR ABC-type transporter family protein; Plant PDR ABC transporter associated domain containing protein, expressed |
| Pavir.J25534 | 3.56763 | Phenylalanine ammonia-lyase 2; phenylalanine ammonia-lyase, putative, expressed |
| Pavir.J34371 | 3.49079 | Aldehyde dehydrogenase 2B4; aldehyde dehydrogenase, putative, expressed |
| Pavir.J23069 | 3.47794 | P-glycoprotein 21; MDR-like ABC transporter, putative, expressed |
| Pavir.Aa00868 | 3.44236 | Plasma membrane intrinsic protein 1;5; aquaporin protein, putative, expressed |
| Pavir.Aa00812 | 3.36591 | Aldehyde dehydrogenase 3H1; aldehyde dehydrogenase, putative, expressed |
| Pavir.Eb02835 | 3.29803 | ATP binding cassette subfamily B4; MDR-like ABC transporter, putative, expressed |
| Pavir.J34946 | 3.29287 | Aldehyde dehydrogenase 3F1; aldehyde dehydrogenase, putative, expressed |
| Pavir.Ab00662 | 3.20187 | ABC transporter family protein; multidrug resistance protein, putative, expressed |
| Pavir.Gb00643 | 3.11671 | Phenylalanine ammonia-lyase 2; phenylalanine ammonia-lyase, putative, expressed |
| Pavir.Aa03129 | 3.11318 | Protein kinase superfamily protein; BRASSINOSTEROID INSENSITIVE 1-associated receptor kinase 1 precursor, putative, expressed |
| Pavir.J37677 | 3.09067 | Plasma membrane intrinsic protein 1;5; aquaporin protein, putative, expressed |
| Pavir.Ia01421 | 3.08766 | NOD26-like intrinsic protein 5;1; aquaporin protein, putative, expressed |
| Pavir.Db01217 | 3.067 | Delta tonoplast integral protein; aquaporin protein, putative, expressed |
| Pavir.Aa00457 | 3.06216 | Aldehyde dehydrogenase 2B4; aldehyde dehydrogenase, putative, expressed |
| Pavir.Bb03208 | 3.05055 | Brassinosteroid signalling positive regulator (BZR1) family protein; BES1/BZR1 homolog protein, putative, expressed |
| Pavir.Ba02483 | 3.02598 | Plasma membrane intrinsic protein 2; aquaporin protein, putative, expressed |
| Pavir.Eb00205 | -3.07384 | ABC transporter family protein; ABC transporter, ATP-binding protein, putative, expressed |
| Pavir.J37276 | -3.13095 | PHE ammonia lyase 1; phenylalanine ammonia-lyase, putative, expressed |
| Pavir.Da00784 | -3.25076 | Multidrug resistance-associated protein 6; ABC transporter, ATP-binding protein, putative, expressed |
| Pavir.Db00271 | -3.29579 | Aluminum sensitive 3; ABC transporter, membrane-spanning/permease subunit, putative, expressed |
| Pavir.J34961 | -3.3851 | General control non-repressible 5; ABC transporter, ATP-binding protein, putative, expressed |
| Pavir.Ea01601 | -3.39942 | Aldehyde dehydrogenase 2C4; aldehyde dehydrogenase, putative, expressed |
| Pavir.J31301 | -3.47669 | Phenylalanine ammonia-lyase 2; phenylalanine ammonia-lyase, putative, expressed |
| Pavir.J18886 | -3.63898 | ABC transporter of the mitochondrion 3; ABC transporter, ATP-binding protein, putative, expressed |
| Pavir.Ha00406 | -3.67129 | General control non-repressible 5; ABC transporter, ATP-binding protein, putative, expressed |
| Pavir.Ab03307 | -3.72565 | ABC transporter family protein; white-brown complex homolog protein, putative, expressed |
| Pavir.J06912 | -3.81225 | General control non-repressible 5; ABC transporter, ATP-binding protein, putative, expressed |
| Pavir.Ab01904 | -4.03494 | ABC transporter family protein; multidrug resistance protein, putative, expressed |
| Pavir.Ga02654 | -4.18423 | Multidrug resistance-associated protein 14; ABC transporter family protein, putative, expressed |
| Pavir.Ga02259 | -4.22223 | Multidrug resistance-associated protein 14; ABC transporter, ATP-binding protein, putative, expressed |
| Pavir.J31685 | -5.09275 | general control non-repressible 5; ABC transporter, ATP-binding protein, putative, expressed |
| Pavir.Bb01841 | -5.23059 | Plasma membrane intrinsic protein 2; aquaporin protein, putative, expressed |
| Pavir.Ba01199 | -5.25504 | Plasma membrane intrinsic protein 3; aquaporin protein, putative, expressed |
| Pavir.Gb00641 | -5.30131 | PHE ammonia lyase 1; phenylalanine ammonia-lyase, putative, expressed |

**Supplementary Table 9: List of stress-related transporters identified in VS16 compared to AP13**

| Gene ID | Log2FC | Description |
| --- | --- | --- |
| Pavir.Ia03597 | 8.75484 | Glutamine-dependent asparagine synthase 1; asparagine synthetase, putative, expressed |
| Pavir.J25709 | 7.52548 | ATP binding cassette subfamily B19; multidrug resistance protein, putative, expressed |
| Pavir.Ea00336 | 7.2087 | Pleiotropic drug resistance 4; pleiotropic drug resistance protein, putative, expressed |
| Pavir.Ib03577 | 6.85242 | White-brown complex homolog protein 11; white-brown complex homolog protein 11, putative, expressed |
| Pavir.J25945 | 6.57426 | Pleiotropic drug resistance 4; pleiotropic drug resistance protein, putative, expressed |
| Pavir.Eb00416 | 6.50819 | Pleiotropic drug resistance 4; pleiotropic drug resistance protein, putative, expressed |
| Pavir.Ga01466 | 6.32578 | ATP binding cassette subfamily B19; multidrug resistance protein, putative, expressed |
| Pavir.J34236 | 6.2879 | ATP binding cassette subfamily B19; multidrug resistance protein, putative, expressed |
| Pavir.Fb02214 | 6.03411 | ATP binding cassette subfamily B1; multidrug resistance protein, putative, expressed |
| Pavir.J19576 | 5.83792 | ABC-2 type transporter family protein; white-brown complex homolog protein, putative, expressed |
| Pavir.Ib01359 | 5.76849 | Asparagine synthetase 2; asparagine synthetase, putative, expressed |
| Pavir.Ga00331 | 5.68279 | ATP binding cassette subfamily B19; multidrug resistance protein, putative, expressed |
| Pavir.J31673 | 5.4752 | Natural resistance-associated macrophage protein 1; natural resistance-associated macrophage protein, putative, expressed |
| Pavir.Aa02082 | 5.38676 | Histidine kinase-, DNA gyrase B-, and HSP90-like ATPase family protein; ATP-binding region, ATPase-like domain containing protein, expressed |
| Pavir.Ba02560 | 5.31009 | Terpenoid cyclases/Protein prenyltransferases superfamily protein; ent-kaurene synthase, chloroplast precursor, putative, expressed |
| Pavir.J02266 | 5.2491 | AAA-type ATPase family protein; ATP binding protein, putative, expressed |
| Pavir.Eb03024 | 5.22463 | AAA-type ATPase family protein; ATP binding protein, putative, expressed |
| Pavir.Bb01507 | 5.17293 | Terpenoid cyclases/Protein prenyltransferases superfamily protein; ent-kaurene synthase, chloroplast precursor, putative, expressed |
| Pavir.Ea02182 | 5.08893 | ABC-2 type transporter family protein; ABC-2 type transporter domain containing protein, expressed |
| Pavir.Ib01122 | 5.08414 | P-loop containing nucleoside triphosphate hydrolases superfamily protein; AAA-type ATPase family protein, putative, expressed |
| Pavir.J06968 | 5.03637 | ABC-2 type transporter family protein; white-brown complex homolog protein 16, putative, expressed |
| Pavir.Ga01595 | 4.954 | P-loop containing nucleoside triphosphate hydrolases superfamily protein; AAA-type ATPase family protein, putative, expressed |
| Pavir.J25378 | 4.93494 | RNAse l inhibitor protein 2; ATP-binding cassette sub-family E member 1, putative, expressed |
| Pavir.J19176 | 4.88246 | AAA-type ATPase family protein; ATP binding protein, putative, expressed |
| Pavir.J08362 | 4.77952 | ABC transporter family protein; ABC transporter, ATP-binding protein, putative, expressed |
| Pavir.Ba02652 | 4.75202 | Terpenoid cyclases/Protein prenyltransferases superfamily protein; ent-kaurene synthase, chloroplast precursor, putative, expressed |
| Pavir.Ia03635 | 4.6734 | ABC-2 type transporter family protein; white-brown complex homolog protein, putative, expressed |
| Pavir.Eb03112 | 4.4229 | AAA-type ATPase family protein; vesicle-fusing ATPase, putative, expressed |
| Pavir.J38164 | 4.40568 | ABC-2 type transporter family protein; white-brown complex homolog protein, putative, expressed |
| Pavir.J36439 | 4.32258 | ABC2 homolog 6; ABC transporter, ATP-binding protein, putative, expressed |
| Pavir.Ab01960 | 4.30495 | Terpenoid cyclases/Protein prenyltransferases superfamily protein; ent-kaurene synthase, chloroplast precursor, putative, expressed |
| Pavir.Eb02933 | 4.28445 | ABC transporter family protein; ABC transporter, ATP-binding protein, putative, expressed |
| Pavir.Ib02089 | 4.27345 | AAA-ATPase 1; ATPase, putative, expressed |
| Pavir.Ib04453 | 4.21788 | ABC-2 type transporter family protein; ABC-2 type transporter, putative, expressed |
| Pavir.Ea02891 | 4.12145 | AAA-type ATPase family protein; vesicle-fusing ATPase, putative, expressed |
| Pavir.Gb00010 | 4.09286 | ABC-2 type transporter family protein; ABC-2 type transporter domain containing protein, expressed |
| Pavir.Gb01753 | 3.98685 | ABC2 homolog 12; ABC transmembrane transporter domain containing protein, expressed |
| Pavir.J14828 | 3.81487 | AAA-type ATPase family protein; ATP binding protein, putative, expressed |
| Pavir.Ea01974 | 3.76667 | ATP binding cassette subfamily B4; MDR-like ABC transporter, putative, expressed |
| Pavir.Bb00325 | 3.74572 | Multidrug resistance-associated protein 3; ABC transporter family protein, putative, expressed |
| Pavir.Ea02141 | 3.7294 | Pleiotropic drug resistance 12; pleiotropic drug resistance protein, putative, expressed |
| Pavir.Ea02141 | 3.7294 | Pleiotropic drug resistance 12; pleiotropic drug resistance protein, putative, expressed |
| Pavir.Gb01095 | 3.69454 | Copper-exporting ATPase / responsive-to-antagonist 1 / copper-transporting ATPase (RAN1); copper-transporting ATPase, putative, expressed |
| Pavir.Ib00655 | 3.66269 | PPPDE putative thiol peptidase family protein; ethylene-responsive element-binding protein, putative, expressed |
| Pavir.Ga02117 | 3.64859 | Natural resistance-associated macrophage protein 1; natural resistance-associated macrophage protein, putative, expressed |
| Pavir.Aa02018 | 3.59202 | Histidine kinase-, DNA gyrase B-, and HSP90-like ATPase family protein; ATP-binding region, ATPase-like domain containing protein, expressed |
| Pavir.Eb02645 | 3.56862 | ABC-2 and Plant PDR ABC-type transporter family protein; Plant PDR ABC transporter associated domain containing protein, expressed |
| Pavir.Eb02645 | 3.56862 | ABC-2 and Plant PDR ABC-type transporter family protein; Plant PDR ABC transporter associated domain containing protein, expressed |
| Pavir.J26981 | 3.56597 | AAA-type ATPase family protein; AAA-type ATPase family protein, putative, expressed |
| Pavir.Ba03649 | 3.49194 | DREB2A-interacting protein 1; zinc finger, C3HC4 type domain containing protein, expressed |
| Pavir.J23069 | 3.47794 | P-glycoprotein 21; MDR-like ABC transporter, putative, expressed |
| Pavir.J06526 | 3.37392 | AAA-type ATPase family protein; AAA-type ATPase family protein, putative, expressed |
| Pavir.J19033 | 3.34757 | Pleiotropic drug resistance 12; pleiotropic drug resistance protein, putative, expressed |
| Pavir.J19033 | 3.34757 | Pleiotropic drug resistance 12; pleiotropic drug resistance protein, putative, expressed |
| Pavir.J14370 | 3.319 | AAA-type ATPase family protein; ATP binding protein, putative, expressed |
| Pavir.Eb02835 | 3.29803 | ATP binding cassette subfamily B4; MDR-like ABC transporter, putative, expressed |
| Pavir.J14256 | 3.26686 | P-loop containing nucleoside triphosphate hydrolases superfamily protein; AAA family ATPase, putative, expressed |
| Pavir.J02112 | 3.26667 | AAA-type ATPase family protein; AAA-type ATPase family protein, putative, expressed |
| Pavir.Ga02349 | 3.2633 | Natural resistance-associated macrophage protein 1; natural resistance-associated macrophage protein, putative, expressed |
| Pavir.J34429 | 3.25191 | AAA-type ATPase family protein; ATP binding protein, putative, expressed |
| Pavir.J11655 | 3.23948 | AAA-type ATPase family protein; ATP binding protein, putative, expressed |
| Pavir.Cb00265 | 3.2152 | DREB2A-interacting protein 2; zinc finger family protein, putative, expressed |
| Pavir.Ab00662 | 3.20187 | ABC transporter family protein; multidrug resistance protein, putative, expressed |
| Pavir.Bb02240 | 3.17125 | P-loop containing nucleoside triphosphate hydrolases superfamily protein; AAA-type ATPase family protein, putative, expressed |
| Pavir.Eb02032 | 3.08385 | Pleiotropic drug resistance 12; pleiotropic drug resistance protein, putative, expressed |
| Pavir.Eb02032 | 3.08385 | Pleiotropic drug resistance 12; pleiotropic drug resistance protein, putative, expressed |
| Pavir.Ba00769 | 3.08256 | Pleiotropic drug resistance 11; pleiotropic drug resistance protein, putative, expressed |
| Pavir.Da00356 | 3.02637 | Histidine kinase-, DNA gyrase B-, and HSP90-like ATPase family protein; ATP-binding region, ATPase-like domain containing protein, expressed |
| Pavir.J23797 | 3.02001 | AAA-ATPase 1; ATPase, putative, expressed |
| Pavir.Ib03865 | -3.01086 | Photosystem II reaction center protein G; NADPH-dependent oxidoreductase, putative, expressed |
| Pavir.Fb02020 | -3.06159 | Mog1/PsbP/DUF1795-like photosystem II reaction center PsbP family protein; thylakoid lumenal 19 kDa protein, chloroplast precursor, putative, expressed |
| Pavir.Eb00205 | -3.07384 | ABC transporter family protein; ABC transporter, ATP-binding protein, putative, expressed |
| Pavir.Ea04142 | -3.10483 | Telomeric repeat binding protein 1; MYB family transcription factor, putative, expressed |
| Pavir.J03342 | -3.11285 | Photosystem II stability/assembly factor, chloroplast (HCF136) |
| Pavir.Bb02107 | -3.132 | Chloroplast Ycf2;ATPase, AAA type, core |
| Pavir.Eb03863 | -3.16223 | Photosystem II reaction center PSB28 protein; photosystem II reaction center PSB28 protein, chloroplast precursor, putative, expressed |
| Pavir.Aa01559 | -3.1755 | ABC2 homolog 13; ABC1 family domain containing protein, putative, expressed |
| Pavir.Fa00505 | -3.18611 | Mog1/PsbP/DUF1795-like photosystem II reaction center PsbP family protein; thylakoid lumenal 19 kDa protein, chloroplast precursor, putative, expressed |
| Pavir.Ea03993 | -3.21283 | Photosystem II reaction center PSB28 protein; photosystem II reaction center PSB28 protein, chloroplast precursor, putative, expressed |
| Pavir.Fa02069 | -3.24444 | Photosystem II subunit R; photosystem II 10 kDa polypeptide, chloroplast precursor, putative, expressed |
| Pavir.Da00784 | -3.25076 | Multidrug resistance-associated protein 6; ABC transporter, ATP-binding protein, putative, expressed |
| Pavir.Ea01699 | -3.26944 | Multidrug resistance-associated protein 5; multidrug resistance-associated protein, putative, expressed |
| Pavir.Db00271 | -3.29579 | Aluminum sensitive 3; ABC transporter, membrane-spanning/permease subunit, putative, expressed |
| Pavir.J22363 | -3.33836 | Photosystem II reaction center PsbP family protein; thylakoid lumen protein, chloroplast precursor, putative, expressed |
| Pavir.Fb00535 | -3.34165 | Photosystem II subunit R; photosystem II 10 kDa polypeptide, chloroplast precursor, putative, expressed |
| Pavir.J34961 | -3.3851 | General control non-repressible 5; ABC transporter, ATP-binding protein, putative, expressed |
| Pavir.J18886 | -3.63898 | ABC transporter of the mitochondrion 3; ABC transporter, ATP-binding protein, putative, expressed |
| Pavir.Ha00406 | -3.67129 | General control non-repressible 5; ABC transporter, ATP-binding protein, putative, expressed |
| Pavir.Fa00447 | -3.70653 | Photosystem II reaction center PsbP family protein; thylakoid lumen protein, chloroplast precursor, putative, expressed |
| Pavir.Ab03307 | -3.72565 | ABC transporter family protein; white-brown complex homolog protein, putative, expressed |
| Pavir.J06912 | -3.81225 | General control non-repressible 5; ABC transporter, ATP-binding protein, putative, expressed |
| Pavir.J07858 | -3.84509 | Photosystem II reaction center W; photosystem II reaction center W protein, chloroplast precursor, putative, expressed |
| Pavir.Ca00274 | -3.84892 | Mog1/PsbP/DUF1795-like photosystem II reaction center PsbP family protein; thylakoid lumenal 29.8 kDa protein, putative, expressed |
| Pavir.Ia04263 | -3.964 | ABC-2 type transporter family protein; ABC-2 type transporter domain containing protein, expressed |
| Pavir.J37487 | -4.00025 | Mog1/PsbP/DUF1795-like photosystem II reaction center PsbP family protein; thylakoid lumenal 29.8 kDa protein, putative, expressed |
| Pavir.Ab01904 | -4.03494 | ABC transporter family protein; multidrug resistance protein, putative, expressed |
| Pavir.Ib03038 | -4.10955 | Pleiotropic drug resistance 3; pleiotropic drug resistance protein, putative, expressed |
| Pavir.J19584 | -4.13721 | Photosystem II reaction center PsbP family protein; thylakoid lumen protein, chloroplast precursor, putative, expressed |
| Pavir.Ga02654 | -4.18423 | Multidrug resistance-associated protein 14; ABC transporter family protein, putative, expressed |
| Pavir.Ia02555 | -4.18919 | Photosystem II reaction center protein D; photosystem II D2 protein, putative, expressed |
| Pavir.Ha01582 | -4.20679 | Photosystem II reaction center protein J; maturase K, putative, expressed |
| Pavir.Ga02259 | -4.22223 | Multidrug resistance-associated protein 14; ABC transporter, ATP-binding protein, putative, expressed |
| Pavir.Gb00905 | -4.28301 | Photosystem-II repair protein, putative, expressed |
| Pavir.J09513 | -4.33546 | Photosystem II reaction center protein D; photosystem II D2 protein, putative, expressed |
| Pavir.Ba01280 | -4.38003 | Dehydration response element B1A; dehydration-responsive element-binding protein, putative, expressed |
| Pavir.Fb02012 | -4.46046 | Photosystem II reaction center PsbP family protein; thylakoid lumen protein, chloroplast precursor, putative, expressed |
| Pavir.Ga01233 | -4.49565 | Photosystem-II repair protein, putative, expressed |
| Pavir.J36530 | -4.5507 | Cation efflux family protein; cation efflux family protein, putative, expressed |
| Pavir.Ca01606 | -4.59766 | Photosystem I, PsaA/PsaB protein; photosystem I P700 chlorophyll a apoprotein A2, putative, expressed |
| Pavir.Eb03560 | -4.66096 | Photosystem II reaction center protein C; photosystem II 44 kDa reaction center protein, putative, expressed |
| Pavir.Bb01530 | -4.67758 | White-brown complex homolog protein 11; white-brown complex homolog protein 11, putative, expressed |
| Pavir.Ca01396 | -4.68342 | Photosystem II reaction center W; photosystem II reaction center W protein, chloroplast precursor, putative, expressed |
| Pavir.Ia03759 | -4.84532 | RNApolymerase sigma subunit 2; RNA polymerase sigma factor, putative, expressed |
| Pavir.Ib00360 | -4.94843 | ABC-2 type transporter family protein; ABC-2 type transporter domain containing protein, expressed |
| Pavir.J31685 | -5.09275 | General control non-repressible 5; ABC transporter, ATP-binding protein, putative, expressed |
| Pavir.J13221 | -5.24601 | Pleiotropic drug resistance 12; pleiotropic drug resistance protein, putative, expressed |
| Pavir.J13221 | -5.24601 | Pleiotropic drug resistance 12; pleiotropic drug resistance protein, putative, expressed |
| Pavir.J06421 | -5.57434 | ABC-2 type transporter family protein; ABC-2 type transporter domain containing protein, expressed |
| Pavir.Fb01115 | -5.66159 | ABC-2 type transporter family protein; ABC-2 type transporter domain containing protein, expressed |
| Pavir.Ib00245 | -5.74326 | ABC-2 type transporter family protein; ABC-2 type transporter domain containing protein, expressed |
| Pavir.Aa00159 | -6.37424 | Non-intrinsic ABC protein 12; white-brown complex homolog protein, putative, expressed |
| Pavir.Bb02969 | -6.86662 | Pleiotropic drug resistance 11; pleiotropic drug resistance protein, putative, expressed |
| Pavir.J09830 | -6.87181 | Photosystem II reaction center PSB28 protein; photosystem II reaction center PSB28 protein, chloroplast precursor, putative, expressed |

**Supplementary Table 10: List of disease-resistant genes identified in VS16 compared to AP13**

| Gene ID | Log2FC | Description |
| --- | --- | --- |
| Pavir.Ga01060 | 8.02562 | Extensin-like protein; LTPL121 - Protease inhibitor/seed storage/LTP family protein precursor, putative, expressed |
| Pavir.Ga01059 | 7.94306 | Bifunctional inhibitor/lipid-transfer protein/seed storage 2S albumin superfamily protein; LTPL122 - Protease inhibitor/seed storage/LTP family protein precursor, expressed |
| Pavir.J14567 | 7.88782 | Bifunctional inhibitor/lipid-transfer protein/seed storage 2S albumin superfamily protein; LTPL128 - Protease inhibitor/seed storage/LTP family protein precursor, expressed |
| Pavir.Ba03729 | 7.46549 | Bifunctional inhibitor/lipid-transfer protein/seed storage 2S albumin superfamily protein; LTPL100 - Protease inhibitor/seed storage/LTP family protein precursor, expressed |
| Pavir.Ib02449 | 6.84531 | Azelaic acid induced 1; LTPL138 - Protease inhibitor/seed storage/LTP family protein precursor, expressed |
| Pavir.J15893 | 6.81939 | Bifunctional inhibitor/lipid-transfer protein/seed storage 2S albumin superfamily protein; LTPL86 - Protease inhibitor/seed storage/LTP family protein precursor, expressed |
| Pavir.Bb00406 | 6.7012 | Bifunctional inhibitor/lipid-transfer protein/seed storage 2S albumin superfamily protein; LTPL100 - Protease inhibitor/seed storage/LTP family protein precursor, expressed |
| Pavir.J35671 | 6.23849 | Bifunctional inhibitor/lipid-transfer protein/seed storage 2S albumin superfamily protein; LTPL73 - Protease inhibitor/seed storage/LTP family protein precursor, expressed |
| Pavir.J38474 | 6.11874 | Bifunctional inhibitor/lipid-transfer protein/seed storage 2S albumin superfamily protein; LTPL140 - Protease inhibitor/seed storage/LTP family protein precursor, expressed |
| Pavir.J13834 | 6.07591 | Bifunctional inhibitor/lipid-transfer protein/seed storage 2S albumin superfamily protein; LTPL113 - Protease inhibitor/seed storage/LTP family protein precursor, expressed |
| Pavir.Ga00323 | 5.94453 | Bifunctional inhibitor/lipid-transfer protein/seed storage 2S albumin superfamily protein; LTPL128 - Protease inhibitor/seed storage/LTP family protein precursor, expressed |
| Pavir.J04379 | 5.87409 | Bifunctional inhibitor/lipid-transfer protein/seed storage 2S albumin superfamily protein; LTPL73 - Protease inhibitor/seed storage/LTP family protein precursor, expressed |
| Pavir.J00057 | 5.68937 | Bifunctional inhibitor/lipid-transfer protein/seed storage 2S albumin superfamily protein; LTPL99 - Protease inhibitor/seed storage/LTP family protein precursor, expressed |
| Pavir.J14421 | 5.63881 | Bifunctional inhibitor/lipid-transfer protein/seed storage 2S albumin superfamily protein; LTPL78 - Protease inhibitor/seed storage/LTP family protein precursor, expressed |
| Pavir.J00056 | 5.49081 | Bifunctional inhibitor/lipid-transfer protein/seed storage 2S albumin superfamily protein; LTPL73 - Protease inhibitor/seed storage/LTP family protein precursor, expressed |
| Pavir.Bb01123 | 5.27264 | Bifunctional inhibitor/lipid-transfer protein/seed storage 2S albumin superfamily protein; LTPL40 - Protease inhibitor/seed storage/LTP family protein precursor, expressed |
| Pavir.Ia00787 | 5.23334 | Bifunctional inhibitor/lipid-transfer protein/seed storage 2S albumin superfamily protein; LTPL73 - Protease inhibitor/seed storage/LTP family protein precursor, expressed |
| Pavir.Bb00998 | 5.07024 | Bifunctional inhibitor/lipid-transfer protein/seed storage 2S albumin superfamily protein; LTPL42 - Protease inhibitor/seed storage/LTP family protein precursor, expressed |
| Pavir.Aa01992 | 4.87754 | Bifunctional inhibitor/lipid-transfer protein/seed storage 2S albumin superfamily protein; LTPL140 - Protease inhibitor/seed storage/LTP family protein precursor, expressed |
| Pavir.Ha01739 | 4.79333 | Syntaxin of plants 61; syntaxin 6, putative, expressed |
| Pavir.J27460 | 4.78876 | Bifunctional inhibitor/lipid-transfer protein/seed storage 2S albumin superfamily protein; LTPL99 - Protease inhibitor/seed storage/LTP family protein precursor, expressed |
| Pavir.Bb00407 | 4.40717 | Bifunctional inhibitor/lipid-transfer protein/seed storage 2S albumin superfamily protein; LTPL100 - Protease inhibitor/seed storage/LTP family protein precursor, expressed |
| Pavir.Hb01787 | 4.38901 | Syntaxin of plants 61; syntaxin 6, putative, expressed |
| Pavir.Ca01052 | 4.34187 | Syntaxin of plants 32; syntaxin, putative, expressed |
| Pavir.J21900 | 3.90469 | Bifunctional inhibitor/lipid-transfer protein/seed storage 2S albumin superfamily protein; LTPL83 - Protease inhibitor/seed storage/LTP family protein precursor, putative, expressed |
| Pavir.J12462 | 3.85412 | Bifunctional inhibitor/lipid-transfer protein/seed storage 2S albumin superfamily protein; LTPL118 - Protease inhibitor/seed storage/LTP family protein precursor, expressed |
| Pavir.J14741 | 3.78598 | Bifunctional inhibitor/lipid-transfer protein/seed storage 2S albumin superfamily protein; LTPL118 - Protease inhibitor/seed storage/LTP family protein precursor, expressed |
| Pavir.Ia01035 | 3.74568 | Bifunctional inhibitor/lipid-transfer protein/seed storage 2S albumin superfamily protein; LTPL71 - Protease inhibitor/seed storage/LTP family protein precursor, expressed |
| Pavir.Ga01775 | 3.35414 | Glycolipid transfer protein (GLTP) family protein; GLTP domain containing protein, putative, expressed |
| Pavir.J25894 | 3.35178 | Bifunctional inhibitor/lipid-transfer protein/seed storage 2S albumin superfamily protein; LTPL85 - Protease inhibitor/seed storage/LTP family protein precursor, expressed |
| Pavir.Db00333 | 3.27109 | Bifunctional inhibitor/lipid-transfer protein/seed storage 2S albumin superfamily protein; LTPL85 - Protease inhibitor/seed storage/LTP family protein precursor, expressed |
| Pavir.Ea03220 | 3.16637 | Bifunctional inhibitor/lipid-transfer protein/seed storage 2S albumin superfamily protein; LTPL65 - Protease inhibitor/seed storage/LTP family protein precursor, expressed |
| Pavir.J14088 | 3.11515 | Lipid transfer protein 3; LTPL14 - Protease inhibitor/seed storage/LTP family protein precursor, expressed |
| Pavir.Aa01606 | -3.28972 | Azelaic acid induced 1; LTPL114 - Protease inhibitor/seed storage/LTP family protein precursor, expressed |
| Pavir.J12674 | -3.95316 | RING/U-box superfamily protein; LTPL9 - Protease inhibitor/seed storage/LTP family protein precursor, expressed |

**Supplementary Table 11: Other important stress-related genes identified in VS16 compared to AP13**

| Gene ID | Log2FC | Description |
| --- | --- | --- |
| Pavir.J06588 | 8.42896 | Cytochrome P450, family 71, subfamily B, polypeptide 37; cytochrome P450, putative, expressed |
| Pavir.Ib03890 | 7.81 | Cytochrome P450, family 71, subfamily B, polypeptide 2; cytochrome P450, putative, expressed |
| Pavir.Db00359 | 7.77268 | Cytochrome P450, family 77, subfamily B, polypeptide 1; cytochrome P450, putative, expressed |
| Pavir.Ea02184 | 7.01089 | Tetratricopetide-repeat thioredoxin-like 1; TTL1, putative, expressed |
| Pavir.Ga01037 | 6.96059 | Cytochrome P450, family 86, subfamily A, polypeptide 4; cytochrome P450, putative, expressed |
| Pavir.Eb01893 | 6.4813 | Tetratricopetide-repeat thioredoxin-like 1; TTL1, putative, expressed |
| Pavir.J23416 | 6.38279 | Tetratricopetide-repeat thioredoxin-like 1; TTL1, putative, expressed |
| Pavir.Ab01476 | 6.34185 | Cytochrome P450, family 76, subfamily C, polypeptide 4; cytochrome P450, putative, expressed |
| Pavir.J09753 | 6.33701 | Cytochrome P450, family 72, subfamily A, polypeptide 15; cytochrome P450 72A1, putative, expressed |
| Pavir.Ga01931 | 6.1496 | Cytochrome P450, family 77, subfamily A, polypeptide 4; cytochrome P450, putative, expressed |
| Pavir.Ib03534 | 6.07324 | Cytochrome P450, family 86, subfamily B, polypeptide 1; cytochrome P450, putative, expressed |
| Pavir.Ab01784 | 6.07018 | Plant calmodulin-binding protein-related; expressed protein |
| Pavir.Bb03711 | 6.04474 | CBL-interacting protein kinase 3; CAMK_KIN1/SNF1/Nim1_like.32 - CAMK includes calcium/calmodulin depedent protein kinases, expressed |
| Pavir.Fa01775 | 6.00733 | Cytochrome P450, family 71, subfamily A, polypeptide 12; cytochrome P450, putative, expressed |
| Pavir.J02052 | 5.81384 | Cytochrome P450, family 90, subfamily D, polypeptide 1; cytochrome P450, putative, expressed |
| Pavir.Ia04278 | 5.80553 | IQ-domain 19; IQ calmodulin-binding motif family protein, putative, expressed |
| Pavir.Cb00638 | 5.79211 | Cytochrome P450, family 716, subfamily A, polypeptide 1; cytochrome P450, putative, expressed |
| Pavir.J09001 | 5.74315 | Cytochrome P450 superfamily protein; cytochrome P450 93A2, putative, expressed |
| Pavir.Ia04862 | 5.63984 | Cytochrome P450, family 96, subfamily A, polypeptide 10; cytochrome P450 protein, putative, expressed |
| Pavir.J09329 | 5.55971 | Cytochrome P450, family 71, subfamily B, polypeptide 36; cytochrome P450, putative, expressed |
| Pavir.Fa01826 | 5.24414 | Cytochrome P450, family 71, subfamily A, polypeptide 12; cytochrome P450, putative, expressed |
| Pavir.J10420 | 5.20575 | Cytochrome P450 superfamily protein; cytochrome P450, putative, expressed |
| Pavir.Gb01082 | 5.19228 | Cytochrome P450, family 86, subfamily A, polypeptide 4; cytochrome P450, putative, expressed |
| Pavir.Ib01757 | 5.18353 | Cytochrome P450 superfamily protein; cytochrome P450, putative, expressed |
| Pavir.Eb02597 | 5.1613 | IQ-domain 28; IQ calmodulin-binding motif family protein, putative, expressed |
| Pavir.Ea02223 | 5.14169 | Cytochrome P450, family 72, subfamily A, polypeptide 14; cytochrome P450 72A1, putative, expressed |
| Pavir.Aa01650 | 5.10052 | Cytochrome P450, family 716, subfamily A, polypeptide 1; cytochrome P450, putative, expressed |
| Pavir.J25822 | 5.09205 | IQ-domain 31; IQ calmodulin-binding motif family protein, putative, expressed |
| Pavir.J22648 | 4.99529 | Allene oxide synthase; cytochrome P450, putative, expressed |
| Pavir.J38842 | 4.90151 | Cytochrome P450, family 716, subfamily A, polypeptide 1; cytochrome P450, putative, expressed |
| Pavir.J14851 | 4.90141 | IQ-domain 14; IQ calmodulin-binding motif domain containing protein, expressed |
| Pavir.J06070 | 4.88962 | Cytochrome P450, family 71, subfamily B, polypeptide 2; cytochrome P450 71A1, putative, expressed |
| Pavir.Ea03767 | 4.86799 | Cytochrome P450, family 94, subfamily C, polypeptide 1; cytochrome P450, putative, expressed |
| Pavir.J11098 | 4.84887 | Cytochrome P450, family 71, subfamily A, polypeptide 12; cytochrome P450, putative, expressed |
| Pavir.J04484 | 4.72644 | BCL-2-associated athanogene 7; IQ calmodulin-binding motif family protein, putative, expressed |
| Pavir.J26956 | 4.71558 | Cytochrome P450 superfamily protein; cytochrome P450, putative, expressed |
| Pavir.Eb00275 | 4.71317 | Cytochrome P450, family 90, subfamily D, polypeptide 1; cytochrome P450, putative, expressed |
| Pavir.J26298 | 4.68736 | Protein kinase superfamily protein; CAMK_CAMK_like.25 - CAMK includes calcium/calmodulin depedent protein kinases, expressed |
| Pavir.J16649 | 4.6646 | Cytochrome P450 superfamily protein; cytochrome P450, putative, expressed |
| Pavir.Ib02986 | 4.64206 | Cytochrome P450 superfamily protein; cytochrome P450, putative, expressed |
| Pavir.Ia04117 | 4.63081 | Cytochrome P450 superfamily protein; cytochrome P450, putative, expressed |
| Pavir.Ea00543 | 4.62861 | Cytochrome P450, family 77, subfamily A, polypeptide 5 pseudogene; muconate cycloisomerase, putative, expressed |
| Pavir.J30784 | 4.62782 | Calcium-binding EF-hand family protein; calcineurin B, putative, expressed |
| Pavir.J32469 | 4.61812 | Cytochrome P450, family 94, subfamily D, polypeptide 1; cytochrome P450, putative, expressed |
| Pavir.Ab01559 | 4.61579 | IQ-domain 6; IQ calmodulin-binding motif family protein, putative, expressed |
| Pavir.Eb00202 | 4.60844 | Tetratricopetide-repeat thioredoxin-like 3; TTL1, putative, expressed |
| Pavir.Ea00158 | 4.5998 | Tetratricopetide-repeat thioredoxin-like 3; TTL1, putative, expressed |
| Pavir.Ba03190 | 4.58266 | Cytochrome p450, family 71, subfamily B, polypeptide 11; cytochrome P450, putative, expressed |
| Pavir.J14829 | 4.5678 | Cytochrome p450, family 71, subfamily B, polypeptide 11; cytochrome P450, putative, expressed |
| Pavir.J28870 | 4.47206 | Cytochrome P450, family 86, subfamily A, polypeptide 2; cytochrome P450, putative, expressed |
| Pavir.J20853 | 4.47128 | Cytochrome P450, family 93, subfamily D, polypeptide 1; cytochrome P450 93A2, putative, expressed |
| Pavir.Ia01141 | 4.46808 | IQ-domain 5; IQ calmodulin-binding motif domain containing protein, expressed |
| Pavir.Ca02650 | 4.44792 | Calmodulin-binding protein; calmodulin binding protein, putative, expressed |
| Pavir.Ab01974 | 4.44658 | Cytochrome P450, family 716, subfamily A, polypeptide 1; cytochrome P450, putative, expressed |
| Pavir.J03601 | 4.44487 | Ataurora3; CAMK_CAMK_like_Aur_like.2 - CAMK includes calcium/calmodulin depedent protein kinases, expressed |
| Pavir.Ea03756 | 4.422 | Chtinase A |
| Pavir.J10570 | 4.37553 | IQ-domain 13; IQ calmodulin-binding motif domain containing protein, expressed |
| Pavir.Ba00257 | 4.3716 | CBL-interacting protein kinase 3; CAMK_KIN1/SNF1/Nim1_like.32 - CAMK includes calcium/calmodulin depedent protein kinases, expressed |
| Pavir.Ib00193 | 4.36714 | Chitinase 16 |
| Pavir.Ga00939 | 4.36161 | IQ-domain 24; IQ calmodulin-binding motif family protein, putative, expressed |
| Pavir.J16453 | 4.35637 | Protein kinase superfamily protein; CAMK_CAMK_like.11 - CAMK includes calcium/calmodulin depedent protein kinases, expressed |
| Pavir.Ab02079 | 4.32777 | Cytochrome P450, family 71, subfamily B, polypeptide 13; cytochrome P450, putative, expressed |
| Pavir.J08910 | 4.2765 | Cytochrome P450, family 93, subfamily D, polypeptide 1; cytochrome P450 93A2, putative, expressed |
| Pavir.J34564 | 4.25235 | Basic Chitinase |
| Pavir.J35469 | 4.22841 | Cytochrome p450, family 71, subfamily B, polypeptide 11; cytochrome P450, putative, expressed |
| Pavir.J04073 | 4.18228 | Cytochrome P450, family 714, subfamily A, polypeptide 1; cytochrome P450, putative, expressed |
| Pavir.J03289 | 4.18129 | Cytochrome P450, family 71, subfamily B, polypeptide 2; cytochrome P450, putative, expressed |
| Pavir.J07533 | 4.07247 | Cytochrome P450, family 716, subfamily A, polypeptide 1; cytochrome P450, putative, expressed |
| Pavir.J02710 | 4.06495 | GA requiring 3; cytochrome P450, putative, expressed |
| Pavir.Ia02865 | 4.00007 | Calcium-dependent protein kinase family protein; CAMK_CAMK_like.8 - CAMK includes calcium/calmodulin depedent protein kinases, expressed |
| Pavir.Gb00888 | 3.98186 | IQ-domain 24; IQ calmodulin-binding motif family protein, putative, expressed |
| Pavir.Da00084 | 3.97737 | Cytochrome P450, family 77, subfamily B, polypeptide 1; cytochrome P450, putative, expressed |
| Pavir.Ia01427 | 3.94662 | Ferulic acid 5-hydroxylase 1; cytochrome P450, putative, expressed |
| Pavir.J14575 | 3.94276 | Cytochrome P450 superfamily protein; cytochrome P450 72A1, putative, expressed |
| Pavir.Ca02742 | 3.9211 | CBL-interacting protein kinase 4; CAMK_KIN1/SNF1/Nim1_like.17 - CAMK includes calcium/calmodulin depedent protein kinases, expressed |
| Pavir.J31916 | 3.88207 | Tetratricopetide-repeat thioredoxin-like 1; TTL1, putative, expressed |
| Pavir.J07908 | 3.87431 | Cytochrome P450 superfamily protein; cytochrome P450, putative, expressed |
| Pavir.Fa02214 | 3.8607 | IQ-domain 17; IQ calmodulin-binding motif domain containing protein, expressed |
| Pavir.J03288 | 3.85583 | Cytochrome P450, family 71, subfamily B, polypeptide 2; cytochrome P450, putative, expressed |
| Pavir.Ib04127 | 3.78762 | IQ-domain 5; IQ calmodulin-binding motif domain containing protein, expressed |
| Pavir.Hb02062 | 3.76652 | Calcium-binding EF-hand family protein; calmodulin-like protein 1, putative, expressed |
| Pavir.Ca02524 | 3.7476 | Protein kinase superfamily protein; CAMK_CAMK_like.49 - CAMK includes calcium/calmodulin depedent protein kinases, expressed |
| Pavir.Ib04305 | 3.73229 | Cytochrome P450, family 71, subfamily B, polypeptide 34; cytochrome P450 71E1, putative, expressed |
| Pavir.J38029 | 3.72873 | Cytochrome P450, family 704, subfamily A, polypeptide 2; cytochrome P450, putative, expressed |
| Pavir.Gb02505 | 3.72319 | Cytochrome P450, family 87, subfamily A, polypeptide 6; cytochrome P450, putative, expressed |
| Pavir.J12708 | 3.6852 | Cytochrome P450, family 703, subfamily A, polypeptide 2; cytochrome P450, putative, expressed |
| Pavir.J34995 | 3.68498 | Cytochrome P450, family 94, subfamily C, polypeptide 1; cytochrome P450, putative, expressed |
| Pavir.Bb03282 | 3.64276 | Cytochrome P450, family 78, subfamily A, polypeptide 10; cytochrome P450, putative, expressed |
| Pavir.Db00475 | 3.56515 | Thioredoxin O1; thioredoxin, putative, expressed |
| Pavir.Cb01264 | 3.56022 | SOS3-interacting protein 1; CAMK_KIN1/SNF1/Nim1_like.20 - CAMK includes calcium/calmodulin depedent protein kinases, expressed |
| Pavir.Ab02051 | 3.55389 | Calmodulin-binding protein; calmodulin binding protein, putative, expressed |
| Pavir.Aa02387 | 3.54891 | IQ-domain 5; IQ calmodulin-binding motif family protein, putative, expressed |
| Pavir.Ga00145 | 3.54526 | IQ-domain 32; IQ calmodulin-binding motif family protein, putative, expressed |
| Pavir.J14918 | 3.49593 | Cytochrome p450, family 71, subfamily B, polypeptide 11; cytochrome P450, putative, expressed |
| Pavir.Ha01053 | 3.45324 | Cytochrome P450, family 71, subfamily A, polypeptide 22; cytochrome P450, putative, expressed |
| Pavir.Ca00154 | 3.43144 | Chloroplast signal recognition particle 54 kDa subunit; signal recognition particle 54 kDa protein, putative, expressed |
| Pavir.J17089 | 3.42627 | Kinesin-like calmodulin-binding protein (ZWICHEL); kinesin motor domain containing protein, putative, expressed |
| Pavir.J31594 | 3.40782 | IQ calmodulin-binding motif family protein, expressed |
| Pavir.J21273 | 3.4075 | Tetratricopetide-repeat thioredoxin-like 1; TTL1, putative, expressed |
| Pavir.Bb03351 | 3.40652 | CDPK-related kinase; CAMK_CAMK_like.35 - CAMK includes calcium/calmodulin depedent protein kinases, expressed |
| Pavir.J13981 | 3.39197 | Cytochrome P450, family 94, subfamily C, polypeptide 1; cytochrome P450, putative, expressed |
| Pavir.Cb00440 | 3.31194 | Protein kinase superfamily protein; CAMK_CAMK_like.49 - CAMK includes calcium/calmodulin depedent protein kinases, expressed |
| Pavir.Ib00724 | 3.29404 | Plant calmodulin-binding protein-related; expressed protein |
| Pavir.J30837 | 3.26699 | Thioredoxin superfamily protein; DSBA-like thioredoxin domain containing protein, expressed |
| Pavir.Gb00253 | 3.16857 | IQ-domain 32; IQ calmodulin-binding motif family protein, putative, expressed |
| Pavir.Ab03214 | 3.14573 | BCL-2-associated athanogene 7; IQ calmodulin-binding motif family protein, putative, expressed |
| Pavir.Eb00692 | 3.12029 | Cytochrome P450, family 77, subfamily A, polypeptide 5 pseudogene; muconate cycloisomerase, putative, expressed |
| Pavir.J34435 | 3.10906 | Cytochrome P450 superfamily protein; cytochrome P450, putative, expressed |
| Pavir.J12237 | 3.10627 | Cytochrome P450, family 77, subfamily A, polypeptide 5 pseudogene; muconate cycloisomerase, putative, expressed |
| Pavir.Ca01558 | 3.09965 | SOS3-interacting protein 1; CAMK_KIN1/SNF1/Nim1_like.20 - CAMK includes calcium/calmodulin depedent protein kinases, expressed |
| Pavir.J02261 | 3.07648 | Cytochrome P450, family 98, subfamily A, polypeptide 3; cytochrome P450, putative, expressed |
| Pavir.J15227 | 3.06057 | Cytochrome P450 72A1, putative, expressed |
| Pavir.J38188 | 3.03687 | IQ-domain 14; IQ calmodulin-binding motif domain containing protein, expressed |
| Pavir.Ea00257 | 3.03261 | Ataurora1; CAMK_CAMK_like_Aur_like.1 - CAMK includes calcium/calmodulin depedent protein kinases, expressed |
| Pavir.Da00560 | 3.02105 | IQ-domain 33; IQ calmodulin-binding motif family protein, putative, expressed |
| 2 Pavir.Da00242 | -3.00206 | Cytochrome P450, family 714, subfamily A, polypeptide 1; cytochrome P450 72A1, putative, expressed |
| Pavir.Ba02133 | -3.02382 | Chloroplastic drought-induced stress protein of 32 kD; thioredoxin, putative, expressed |
| Pavir.J27308 | -3.07459 | Cytochrome P450, family 93, subfamily D, polypeptide 1; cytochrome P450, putative, expressed |
| Pavir.J03503 | -3.08803 | mRNA splicing factor, thioredoxin-like U5 snRNP; mitosis protein dim1, putative, expressed |
| Pavir.Ia04505 | -3.09752 | CBL-interacting protein kinase 9; CAMK_KIN1/SNF1/Nim1_like.15 - CAMK includes calcium/calmodulin depedent protein kinases, expressed |
| Pavir.Hb00190 | -3.10181 | Calmodulin binding protein-like; NB-ARC domain containing protein, expressed |
| Pavir.J07094 | -3.10814 | Ferredoxin thioredoxin reductase catalytic beta chain family protein; ferredoxin-thioredoxin reductase catalytic chain, chloroplast precursor, putative, expressed |
| Pavir.J02213 | -3.13121 | Cytochrome P450, family 81, subfamily D, polypeptide 5; cytochrome P450, putative, expressed |
| Pavir.Db02051 | -3.13414 | Ferredoxin thioredoxin reductase catalytic beta chain family protein; ferredoxin-thioredoxin reductase catalytic chain, chloroplast precursor, putative, expressed |
| Pavir.Gb01955 | -3.1466 | Calmodulin-binding family protein; calmodulin-binding protein, putative, expressed |
| Pavir.Ea03575 | -3.16862 | BTB and TAZ domain protein 3; BTBZ1 - Bric-a-Brac, Tramtrack, and Broad Complex BTB domain with TAZ zinc finger and Calmodulin-binding domains, expressed |
| Pavir.J04572 | -3.20805 | Alpha/beta-Hydrolases superfamily protein; calmodulin-binding heat-shock protein, putative, expressed |
| Pavir.J23270 | -3.22148 | Calmodulin binding protein, putative, expressed |
| Pavir.J24751 | -3.25932 | Phosphoenolpyruvate carboxylase kinase 1; CAMK_CAMK_like.14 - CAMK includes calcium/calmodulin depedent protein kinases, expressed |
| Pavir.J33567 | -3.26382 | Calmodulin-binding protein; calmodulin binding protein, putative, expressed |
| Pavir.Aa01275 | -3.30529 | Phosphoenolpyruvate carboxylase kinase 1; CAMK_CAMK_like.14 - CAMK includes calcium/calmodulin depedent protein kinases, expressed |
| Pavir.Eb00003 | -3.36002 | Cytochrome P450, family 71, subfamily A, polypeptide 21; cytochrome P450, putative, expressed |
| Pavir.Ea01032 | -3.36232 | CBL-interacting protein kinase 1; CAMK_KIN1/SNF1/Nim1_like.9 - CAMK includes calcium/calmodulin depedent protein kinases, expressed |
| Pavir.J14234 | -3.36468 | Thioredoxin superfamily protein; peroxiredoxin, putative, expressed |
| Pavir.J17625 | -3.39161 | Cytochrome P450 superfamily protein; cytochrome P450, putative, expressed |
| Pavir.Db01969 | -3.39462 | Thioredoxin superfamily protein; peroxiredoxin, putative, expressed |
| Pavir.Ea02316 | -3.4228 | Chtinase A |
| Pavir.J34046 | -3.45353 | Cytochrome P450, family 72, subfamily A, polypeptide 15; cytochrome P450 72A1, putative, expressed |
| Pavir.J36303 | -3.45486 | Thioredoxin F2; thioredoxin, putative, expressed |
| Pavir.Ia03619 | -3.45855 | Thioredoxin superfamily protein; expressed protein |
| Pavir.J01689 | -3.47848 | BTB and TAZ domain protein 3; BTBZ1 - Bric-a-Brac, Tramtrack, and Broad Complex BTB domain with TAZ zinc finger and Calmodulin-binding domains, expressed |
| Pavir.J03127 | -3.4898 | Cytochrome P450, family 714, subfamily A, polypeptide 1; cytochrome P450, putative, expressed |
| Pavir.J05746 | -3.50655 | Thioredoxin F2; thioredoxin, putative, expressed |
| Pavir.J05516 | -3.51725 | Cytochrome P450, family 72, subfamily A, polypeptide 14; cytochrome P450 72A1, putative, expressed |
| Pavir.J27800 | -3.54605 | Calmodulin-binding family protein; calmodulin-binding protein, putative, expressed |
| Pavir.J12506 | -3.55825 | IQ-domain 5; IQ calmodulin-binding motif domain containing protein, expressed |
| Pavir.Ha00641 | -3.56008 | IQ calmodulin-binding and BAG domain containing protein, putative, expressed |
| Pavir.Ia01083 | -3.57918 | Cytochrome P450, family 87, subfamily A, polypeptide 2; cytochrome P450, putative, expressed |
| Pavir.Ba01889 | -3.77273 | Cytochrome P450 superfamily protein; cytochrome P450, putative, expressed |
| Pavir.Eb00289 | -3.81145 | Thioredoxin superfamily protein; OsGrx_A2 - glutaredoxin subgroup III, expressed |
| Pavir.J28170 | -3.84293 | Thioredoxin H-type 7; thioredoxin, putative, expressed |
| Pavir.Fa00832 | -3.88633 | Cytochrome P450, family 76, subfamily C, polypeptide 2; cytochrome P450, putative, expressed |
| Pavir.J26953 | -3.97474 | Atypical CYS HIS rich thioredoxin 2; thioredoxin, putative, expressed |
| Pavir.Ga01283 | -4.03764 | Cytochrome P450, family 71, subfamily B, polypeptide 2; cytochrome P450, putative, expressed |
| Pavir.Ab00798 | -4.0587 | Chitnase 4 |
| Pavir.Ab00798 | -4.0587 | Basic Chitinase |
| Pavir.J38813 | -4.06868 | Cytochrome P450, family 707, subfamily A, polypeptide 4; cytochrome P450, putative, expressed |
| Pavir.J01260 | -4.0956 | Thioredoxin superfamily protein; expressed protein |
| Pavir.J35865 | -4.10333 | Thioredoxin superfamily protein; expressed protein |
| Pavir.Gb00065 | -4.11389 | Thioredoxin X; thioredoxin, putative, expressed |
| Pavir.J20582 | -4.12078 | Phosphoenolpyruvate carboxylase kinase 1; CAMK_CAMK_like.14 - CAMK includes calcium/calmodulin depedent protein kinases, expressed |
| Pavir.J24864 | -4.16364 | Chitinase 2 |
| Pavir.J26358 | -4.21919 | Thioredoxin superfamily protein; expressed protein |
| Pavir.Da00734 | -4.26965 | Cytochrome P450, family 87, subfamily A, polypeptide 9; cytochrome P450, putative, expressed |
| Pavir.Ha01884 | -4.27393 | CBL-interacting protein kinase 3; CAMK_KIN1/SNF1/Nim1_like.37 - CAMK includes calcium/calmodulin depedent protein kinases, expressed |
| Pavir.J40091 | -4.28229 | Cytochrome P450, family 714, subfamily A, polypeptide 1; cytochrome P450 72A1, putative, expressed |
| Pavir.Ib00871 | -4.30914 | Cytochrome P450, family 76, subfamily C, polypeptide 1; cytochrome P450, putative, expressed |
| Pavir.J28406 | -4.31923 | Thioredoxin superfamily protein; expressed protein |
| Pavir.J10796 | -4.59569 | Cytochrome P450, family 709, subfamily B, polypeptide 2; cytochrome P450 72A1, putative, expressed |
| Pavir.J32909 | -4.62636 | Ferredoxin/thioredoxin reductase subunit A (variable subunit) 2; ferredoxin-thioredoxin reductase, variable chain, putative, expressed |
| Pavir.Ia00714 | -4.69108 | Thioredoxin superfamily protein; expressed protein |
| Pavir.Ha00715 | -4.81133 | Cytochrome P450, family 716, subfamily A, polypeptide 1; cytochrome P450, putative, expressed |
| Pavir.Da01689 | -4.90659 | Cytochrome P450 superfamily protein; cytochrome P450, putative, expressed |
| Pavir.Ia00715 | -5.18689 | Thioredoxin superfamily protein; expressed protein |
| Pavir.Hb00116 | -5.18783 | Calmodulin-binding protein; calmodulin binding protein, putative, expressed |
| Pavir.Aa02817 | -5.32122 | NADPH-dependent thioredoxin reductase C; bifunctional thioredoxin reductase/thioredoxin, putative, expressed |
| Pavir.Ib03354 | -5.35319 | Cytochrome P450 superfamily protein; cytochrome P450, putative, expressed |
| Pavir.Ia03284 | -5.41344 | Cytochrome P450, family 709, subfamily B, polypeptide 3; cytochrome P450 72A1, putative, expressed |
| Pavir.Cb01800 | -5.42569 | Thioredoxin superfamily protein; thioredoxin, putative, expressed |
| Pavir.J01458 | -5.81263 | Ataurora3; CAMK_CAMK_like_Aur_like.2 - CAMK includes calcium/calmodulin depedent protein kinases, expressed |
| Pavir.J40555 | -7.06753 | Cytochrome P450, family 71, subfamily B, polypeptide 23; cytochrome P450, putative, expressed |
| Pavir.Ia01888 | -7.24124 | Cytochrome P450 superfamily protein; cytochrome P450, putative, expressed |
| Pavir.J36245 | -9.84065 | Thioredoxin superfamily protein; thioredoxin, putative, expressed |
